# Full Dynamic Range Quantification using Loop-mediated Amplification (LAMP) by Combining Analysis of Amplification Timing and Variance between Replicates at Low Copy Number

**DOI:** 10.1038/s41598-020-57473-1

**Published:** 2020-01-22

**Authors:** Patrick Hardinge, James A. H. Murray

**Affiliations:** Cardiff School of Biosciences, Biomedical Science Building, Museum Avenue, Cardiff, CF10 3AX UK

**Keywords:** DNA synthesis, DNA, Biochemical assays

## Abstract

Quantification of nucleic acid targets at low copy number is problematic with the limit of detection at 95 percent confidence predicted to be 3 molecules or higher for quantitative PCR. Conversely the accuracy of digital PCR is diminished at higher concentrations of template approaching 100 percent positive partitions, with the Poisson distribution showing that an average of only 3 molecules per partition represents an amplification frequency of greater than 95 percent. Therefore a full range of template concentrations cannot be quantified accurately with these methods alone without dilution. Here we report the development of quantification metrics for use with loop-mediated amplification (LAMP) as a bridge between concentrated and dilute template concentrations. The basis for this is that real-time monitoring of LAMP reactions either by bioluminescent reporting (BART) or by fluorescent dye binding shows increasing variation in timings between replicates at low copy number due to the LAMP amplification mechanism. This effect increases with decreasing copy number, closely associated with the amplification frequency. The use of an artificial template showed that the increasing variation is not linked to the use of displacement primers during the initiation of amplification and is therefore a fundamental feature of the LAMP initiation event. Quantification between 1 and 10 copies of a template was successfully achieved with a number of methods with a low number of replicates with the strongest correlation to timing variance. These ultra-quantification methods for LAMP amplification either singularly or in combination have potential in a full dynamic range quantification strategy based on LAMP, in a closed tube, undiluted sample molecular diagnostic.

## Introduction

Full dynamic range quantification in a closed tube format using an isothermal amplification technology with bioluminescent or fluorescent detection would be highly desirable, but is hampered by low copy number quantification in the range lying between concentrations suitable for digital and traditional DNA quantification techniques. We herein propose a method to bridge this gap.

The benchmark quantification method for nucleic acid targets in molecular diagnostics is the quantitative real-time polymerase chain reaction (qPCR). For qPCR, Forootan et al (2017)^1^ showed that the theoretical limit of detection at 95 percent confidence is 3 molecules, and Ståhlberg et al (2014)^2^ showed that this can be improved to 2.5, but in practical terms such limits of detection are hard to achieve due to such factors as number of replicates, sampling noise, failure to amplify and template absorption to surfaces.

DNA target amplification with digital PCR (dPCR) quantifies using the Poisson distribution of positive and negative partitions to provide absolute quantification without the need for calibration. The dynamic range for dPCR is limited because the target concentration is required to be sufficiently dilute to be in range between 10 percent positives to 97 percent positives, and the oligonucleotide primers are assumed to amplify with 100 percent efficiency. The expected percentage positive partitions for an average of 1 copy per partition is 63 percent, for two copies 86 percent, three copies 95 percent and for four copies per partition 98 percent. Therefore differentiating 3 to 4 copies requires multiple partitions for accurate quantification. For highly dilute samples increased precision comes from very large numbers of partitions, for example the Quant Studio 3D from Applied Biosystems uses chips with a maximum of 20 thousand partitions and the RainDrop Plus Digital PCR System from RainDance Technologies can generate up to 10 million droplet partitions. However the use of the dilutions required itself introduces potential errors.

**Fig. 1:**
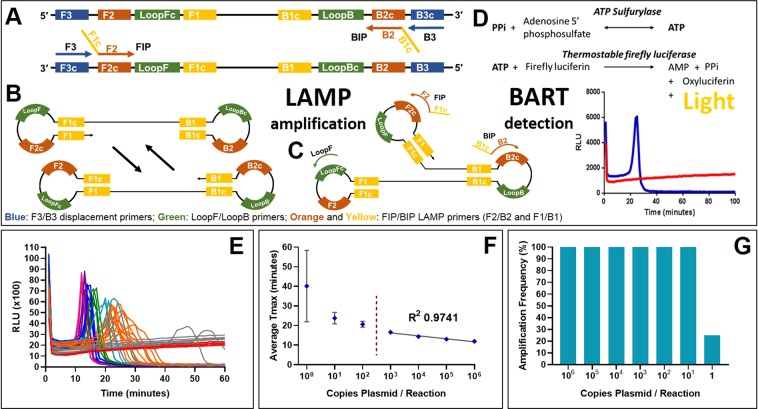
LAMP mechanism, BART detection and variation between replicates at low copy number. (**A**) The LAMP and displacement primers invade double stranded DNA to initiate amplification with a displacement polymerase. (**B**) Looped structures are formed which are elongated with further LAMP primers and the addition of Loop primers (**C**). The pyrophosphate by-product of DNA amplification is converted into ATP which is utilised by a thermostable luciferase to produce detectable bioluminescence in the BART reaction (**D**). For the positive sample in blue, the light increases until the inhibition of luciferase by high concentrations of pyrophosphate. The negative sample in red maintains a background level of bioluminescence. The time-to-peak is directly proportional to the original DNA concentration of the positive sample. (**E**) A ten-fold dilution series from 10^6^ to 1 copy per reaction (pink 10^6^ copies, purple 10^5^, dark blue 10^4^, green 10^3^, light blue 100, orange 10, grey 1, red for no template control) shows the linear relationship between average time-to-peak and log template concentration between 10^6^ and 10^3^ (**F**). Lower copy numbers show increased variation between replicates. Below 10 copies the amplification frequency reduces from 100 percent (**G**).

PCR amplification requires thermocycling to denature, anneal and extend the nascent strands. Over the last decade a number of new DNA amplification methods have been developed that do not require thermocycling. These isothermal amplification methods^3^^4^^5^ have attracted particular interest in molecular biology due to reduced equipment costs associated with maintaining a single temperature, as well as high specificity and sensitivity. One such method is Loop-mediated amplification (LAMP)^6^ which uses loop forming and displacement oligonucleotide primers and a strand displacing polymerase to initiate and cycle amplification (Fig. 1 panel **A to C**). Further loop^7^ or stem primers^8^ can be incorporated into the reaction to increase the rate of DNA formation.

The progress of LAMP amplification can be determined by both end point and real time methods that measure either the increase in double stranded DNA or inorganic pyrophosphate produced. In the work reported here, we have used both a real time fluorescent method with an intercalating SYTO dye^9^^10^^11^ and a bioluminescent reporter BART (bioluminescent assay in real time)^12^. BART couples ATP generation from inorganic pyrophosphate to light output from a thermostable luciferase in a closed tube assay. The light output increases with increasing pyrophosphate from DNA amplification until the concentration of pyrophosphate inhibits the luciferase causing a rapid decline in light output. The assay time to this peak is strongly proportional to the concentration of the DNA template (Fig. 1 panel **D**), providing a method for effective quantification based on time to peak light output.

We have previously observed that the timing of amplification with LAMP of multiple replicates at low copy number shows a marked increase in standard deviation when compared to higher copy numbers^12^. Here we show this increase of variance is a universal feature of LAMP and that the variance increases with decreasing copy number. We show that the inverse relationship between variance and copy number is maintained in the absence of LAMP displacement primers. We further investigate the minimum number of replicates required to clearly separate the variance values from different copy numbers and show the effect of denaturation on genomic DNA. We also explore the reproducibility of the variance metric and propose other metrics based on the timings of the LAMP replicates. The association of the variance metric with amplification frequency suggests a methodology to bridge between digital LAMP and higher copy number quantification creating the possibility of full dynamic range quantification within a closed tube format without requirement for sample dilution.

## Results

### LAMP and PCR variation between replicates at low copy number

**Fig. 2:**
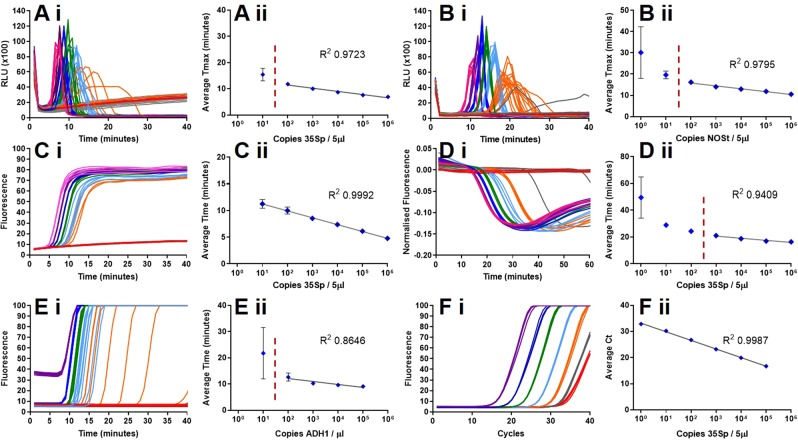
Variance at low copy number is a property of LAMP. Output fluorescent or bioluminescent results for DNA template dilution series (**i**), no template control in red, calculated average of 1 copy per partition in grey, 10 copies in orange, 10^2^ copies in light blue, 10^3^ copies in green, 10^4^ in dark blue, 10^5^ in purple and 10^6^ in pink. Average Ct or Tmax against DNA template concentration (logarithmic scale) (**ii**) with trendline and error bars. (**A**) LAMP amplification with 35Sp primers, artificial template and detection with BART; (**B**) LAMP amplification with NOSt primers, artificial template and BART detection; (**C**) LAMP amplification with 35Sp primers, artificial template and SYTO9 dye detection; (**D**) LAMP amplification with 35Sp primers, linearised plasmid and JOE-FIP quenched fluorescence detection; (**E**) LAMP with ADH1 primers, genomic DNA template and SYTO9 detection; (**F**) PCR with 35Sp primers, genomic template and SYBR green detection.

The timing of LAMP amplification measured by fluorescence (Fig. 2C i,C ii) or bioluminescence (Fig. 2A i,A ii) detection is proportional to target copy number over a broad range, but we have previously reported that variance increased at low copy number. Investigation confirmed that variation between replicates with loop-mediated amplification (LAMP) and BART bioluminescent detection using 35S promoter primers and linearised plasmid DNA template increased with decreasing template concentration (Fig. 1E,F). This observation was confirmed with different primers, templates, detection strategies and an alternative nucleic acid amplification technology (Fig. 2).

Increased variation between replicates was consistently observed at low copy number with all LAMP assays with primers targeting two independent sequences (NOSt or 35Sp), genomic, plasmid and artificial DNA templates and fluorescent and bioluminescent detection strategies. PCR of the same genomic template showed no increase in variation between replicates at low copy number which leads us to conclude that the variation is an inherent property of LAMP amplification at low copy number.

We investigated a number of methods to distinguish between low copy numbers based on the timings of the LAMP reaction from multiple partitions, including the average of the Time (minutes)/Tmax values, the median, the mode and the fastest of the results (Supplementary Information Fig. S3). Although linearity was shown with these methods the reliance on reaction timings can be affected by slight changes in LAMP reaction conditions.

### LAMP amplification at low copy number

**Fig. 3:**
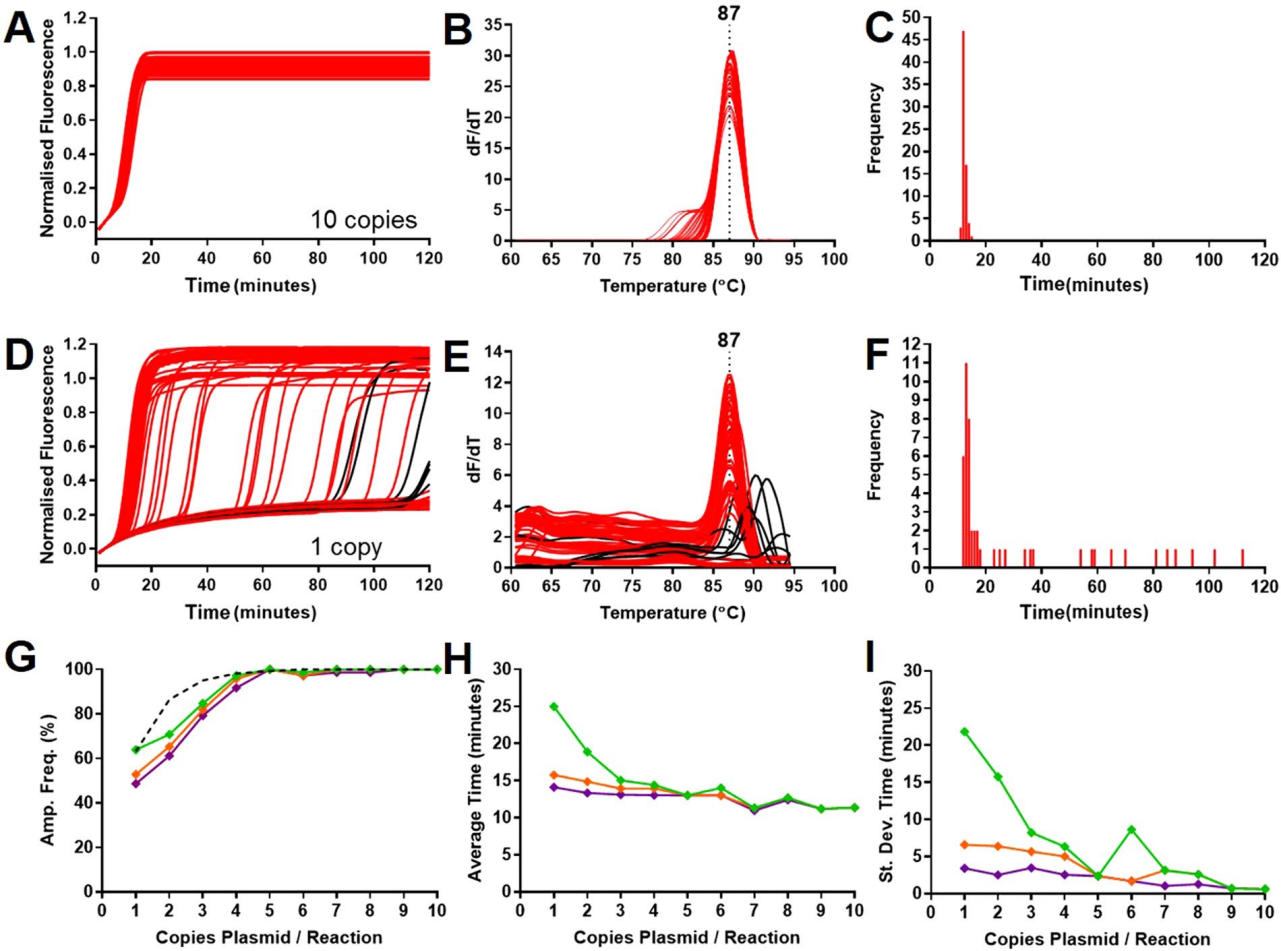
Variation between replicates and amplification frequency at low copy number. (**A**) 10 copies of linearised plasmid per reaction shows low variation between 72 replicates, (**B**) melt curves centred on 87 degrees C and (**C**) Time frequency distribution. (**D**) Increased variation between replicates observed at 1 copy with possible false positives (in black) corresponding to higher melt temperatures (**E**) corresponding to positive results after 80 minutes. The frequency distribution (**F**) shows the increased variation of positive results at low copy number. The amplification frequency from the 1 to 10 copies per partition data are compared to the predicted amplification frequency (dotted line) based on digital PCR calculations. (**G**) Amplification frequencies for positive results less than 90 minutes (green), 40 minutes (orange) and 30 minutes (purple) with (**H**) average Time values at each copy number for results less than 90, 40 and 30 minutes (error bars removed for simplicity) and (**I**) variance for assay results less than 90, 40 and 30 minutes.

Since the variance increased below an average of 10 copies, LAMP amplification with 35Sp primers and the linearised plasmid template was used to investigate multiple replicates at calculated averages of 10 copies per partition incrementally to 1 copy per partition. SYTO9 fluorescence detection was used to track the amplification and amplicons were analysed from melt curve data (Fig. 3).

The LAMP amplification of 35Sp linearised plasmid template at 10 copies per partition showed a low variation between 72 replicates with all results obtained in less than 20 minutes (Fig. 3A). All the positive results (100 percent amplification frequency) produced melt curves centred on 87 degrees C. The amplification frequency shows that over half the positive replicates are at the same Time (minutes) value. In contrast at 1 copy per partition, high variation between the 72 replicates is observed with only 11 of the positive replicates at the same Time (minutes) (mode). The melt curve analysis showed the majority of positive results centred on 87 degrees C, but also others with higher temperature peaks (marked in black). These peaks correspond with positive results observed at times greater than 80 minutes and could be indicative of ’false positives’ from non specific primer interactions. Analysis of data from 1 copy to 10 copies per partition showed that the amplification frequency was similar to the predicted percentage of positive partitions from digital PCR calculations. Both the average Time (minutes) and standard deviation (variance) analysis showed an increase with decreasing copy number. (Results and analysis of data for the 1 to 10 copies per partition are in Supplementary Information; Figs. S1, S2, S3).

### Quenched fluorescent primer LAMP

**Fig. 4:**
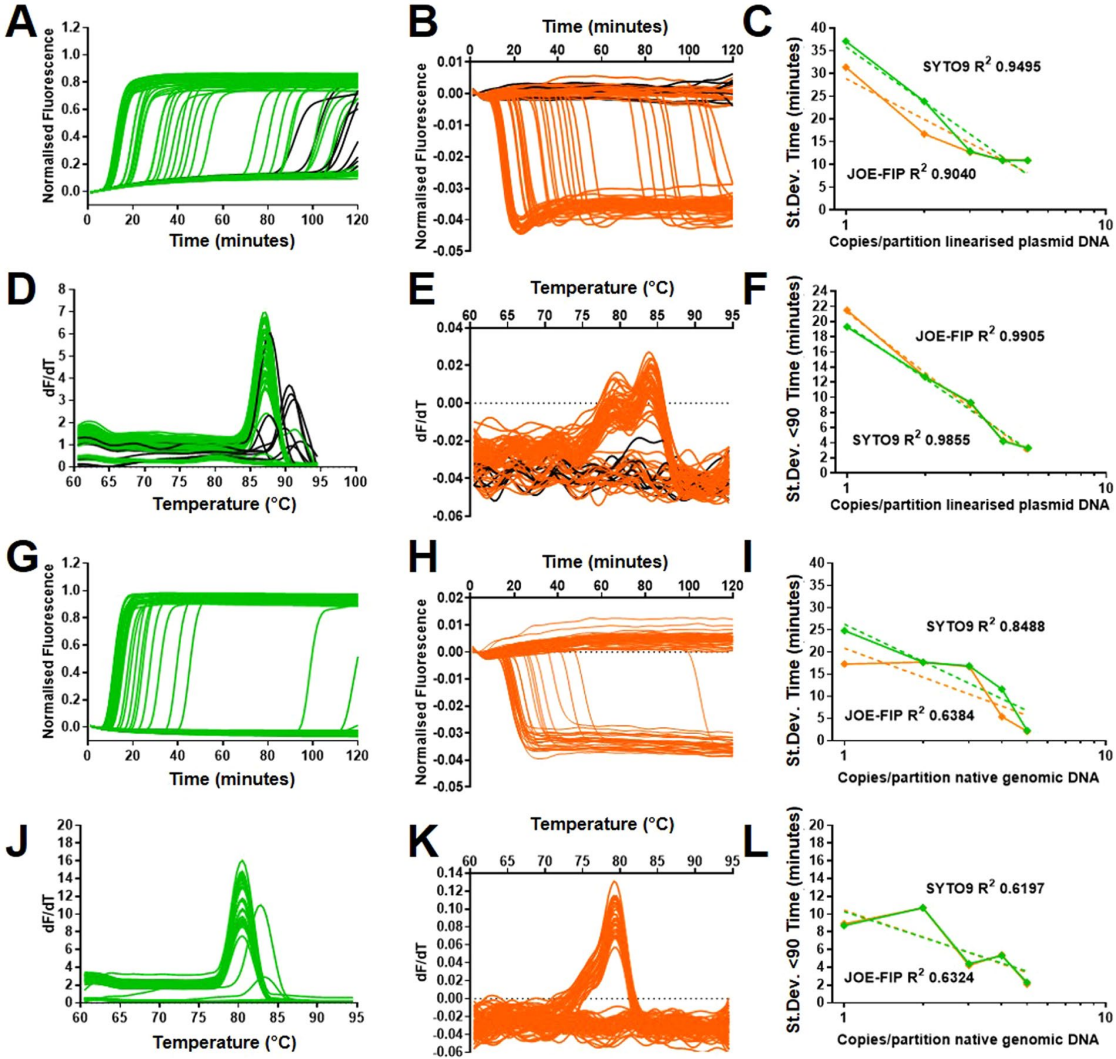
SYTO9/JOE-FIP dual detection for false positives from 35Sp and NOSt LAMP primers. (**A,B,D,E**) 35Sp LAMP amplification of calculated average of 1 copy of linearised plasmid DNA with JOE labelled FIP primer for quenched fluorescence detection with SYTO9 detection. (**A**) SYTO9 results in green with associated melt curve analysis (**D**). (**C**) JOE-FIP results in orange with positive results not detected by JOE-FIP in black and melt curve (**E**). (**G,H,J,K**) NOSt LAMP amplification of average 1 copy of native genomic (maize Bt11) DNA with quenched fluorescence and SYTO9 detections. (**G**) SYTO9 results in green with associated melt curve analysis (**J**). (**H**) JOE-FIP results in orange with positive results not detected by JOE-FIP in black and melt curve (**K**). Variance for 120 minute assay (**C** and **I**) and truncated to 90 minutes (**F** and **L**) with difference between SYTO9 (green) and JOE-FIP (orange) detected results at 1 to 5 copies per reaction on a logarithmic scale with trendlines and best fit values (results for 2, 3, 4 and 5 copies SYTO9/JOE-FIP for 35Sp and NOSt in Supplementary Information Figs. S4, S5, S6 and S7).

The possibility of ’false positives’ late in the assays may influence the use of variance as a metric for low copy number LAMP quantification and was therefore investigated. The 35Sp and NOSt LAMP primer sets where used to amplify linearised plasmid and genomic DNA templates (Fig. 4) with the quenched fluorescent primer method^13^.This involves the use of fluorescent dyes linked to FIP or BIP primers that are quenched during LAMP amplification with concomitant reduction in the fluorescent signal. The decreased fluorescence is associated with LAMP amplification and does not occur with interactions between the displacement and loop primers that can cause false positives. Assays were therefore carried out using the fluorophore JOE, attached to the FIP primers together with SYTO9 fluorescence for amplicon detection, to identify potential non-specific LAMP primer interactions.

The 35Sp LAMP assay of linearised plasmid DNA showed a number of positives with SYTO9 that were not confirmed with the quenched fluorescent primer method. These are highlighted in black and show their late assay timings above 80 Time (minutes) in the experiment with an average of 1 copy per partition (Fig. 4A,B). The SYTO9 melt curve analysis shows that the majority of these highlighted partitions contained amplicons with melt temperatures above 87 degrees C (Fig. 4D). This data, combined with results for calculated average 2, 3, 4 and 5 copy per partition for SYTO9 and JOE-FIP quenched fluorescence detection are plotted to show the effect on the variance metric by potential false positives partitions (Fig. 4C,F). Using all the data for the 120 minute assays, the SYTO9 results had higher values at 1 and 2 copies than excluding those assays not positive with JOE-FIP due to the additional late results. Truncating the results to 90 minutes reduced the separation between the two methods due to the exclusion of the majority of false positives. The fit to a linear trendline of the data points on a semi-logarithmic graph of copy number against the standard deviation for both datasets is high with R^2^ values above 0.98 for both.

Additional experiments were carried out with a different primer set targeting NOSt. Genomic DNA was amplified with LAMP and NOSt primers with SYTO9 and JOE-FIP detection diluted to average 1 copy per partition (Fig. 4G,H). No additional positive partitions with SYTO9 were observed compared to the quenched fluorescence in the 120 minute assay that could be attributable to non-specific primer interaction. However a late peak nearing 120 minutes was seen only with SYTO9 and not with JOE-FIP possibly due to the slight lag between the two methods. The late results for SYTO9 in the 1 copy and 4 copies per partition have an impact on the variation between replicates giving slightly higher results when all the data is used and parity with JOE-FIP detection with the truncated to 90 Time (minutes) results. Although this NOSt LAMP assay was unaffected by false positives, the linearity of the relationship between target copy number and standard deviation was reduced. The NOSt LAMP assay targetted genomic DNA template whereas the 35Sp assay targeted linearised plasmid DNA which could introduce additional variability.

### Number of required replicates

**Fig. 5:**
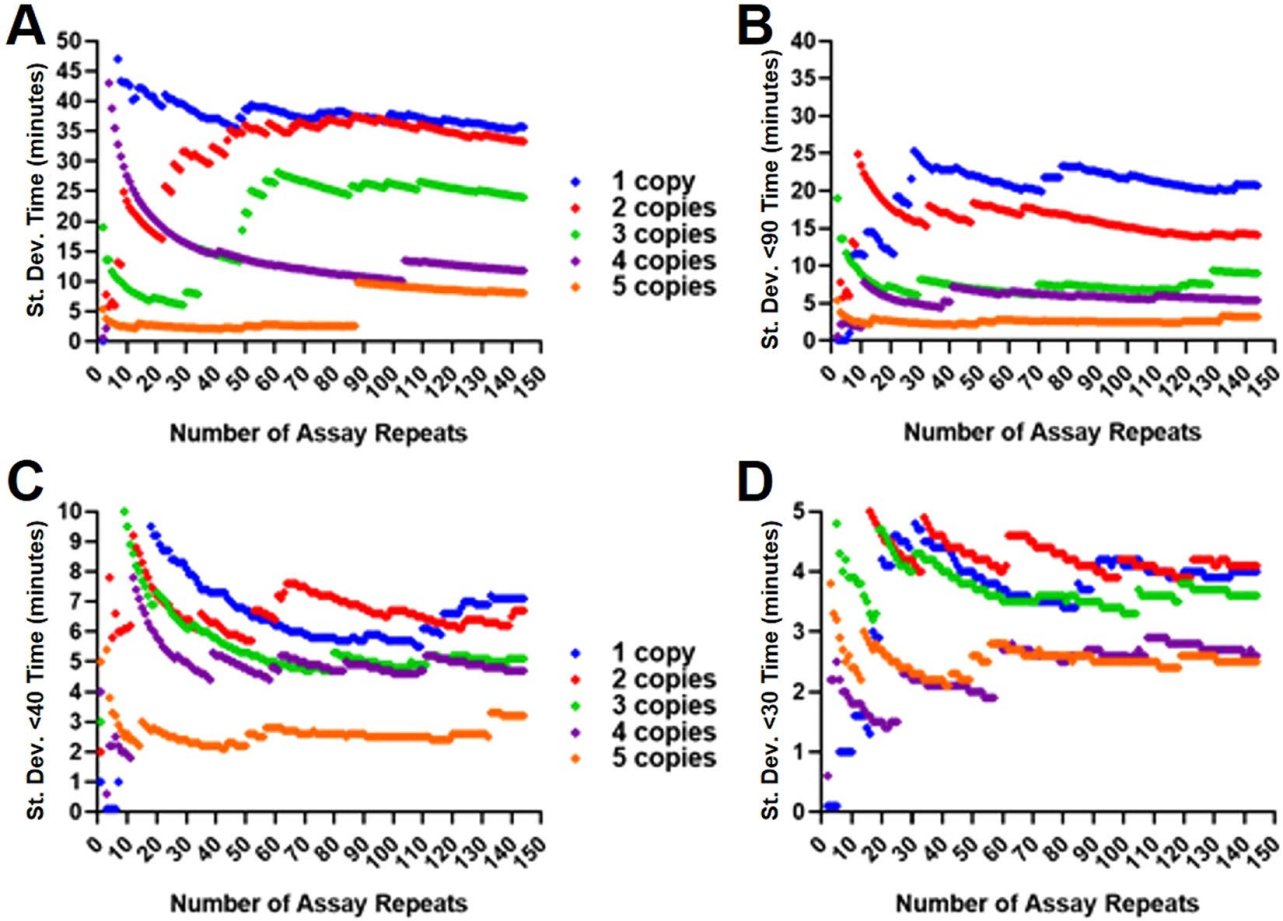
Randomised replicate Time values to show separation between 1 and 5 copies with increasing number of replicates. The order in which the data was analysed was randomised and the variance calculated from increasing number of assay replicates for the linearised plasmid 35Sp LAMP results from calculated average 1 to 5 copies per reaction. Total assay replicates of 144 from two repeat assays, with total assay time of 120 minutes (**A**), truncated to 90 (**B**), 40 (**C**) and 30 minutes (**D**). Blue: 1 copy; Red: 2 copies; Light Green: 3 copies; Purple: 4 copies; Orange: 5 copies (the random numbers are listed in the methods).

To investigate the variation between replicates at low copy number in more detail, and to explore both the required number of replicates and the length of assay needed to obtain an accurate reflection of average copy number, an average of 1 to 5 copies per partition of the 35Sp linearised plasmid template were LAMP assayed with 144 replicates. Truncating the data would potentially reduce the impact of late false positive and the assays would be quicker to complete. The order of the position on the plates of each partition was randomised (see methods) to eliminate any positional bias. The standard deviation for each copy number was calculated and plotted against the number of replicates used for the calculation (Fig. 5).

Separation between the calculated variance of the five copy number levels was clear and in the correct order from 98 replicates using the untruncated randomised data (Fig. 5A). The experiment with average of 5 copies per partition showed a consistent trace until an outlying partition with Time (minutes) value of 103.3 minutes at 88 replicates increased the variance from 2.6 to 9.8 minutes. The 5 copy trace however maintains lower variance than the 4 copy . All copy number traces appear to become more robust after approximately 110 replicates with a more gradual change in variance. The data truncated to a maximum of 90 minutes (Fig. 5B) shows a consistent trace from the first calculations through to 144 replicates. There is clear separation and in the correct order from 70 replicates and the separation between 1, 2 and 3 copies per partition is improved. Further truncation to 40 and 30 minutes reduced the values achievable for variance and consequently the traces were close together. For the 40 minute data (Fig. 5C) the correct order from 5 to 1 copies was achieved from 119 replicates onward, but the correct order was not achieved for data truncated at 30 minute (Fig. 5D). (Alternative template and LAMP primer combinations are shown in Supplementary Information; Fig. S8). We conclude that truncation of the data can help eliminate false positives and improve assay speed and reliabililty, but the appropriate choice of truncation time needs to be optimised for the assay in question.

### LAMP replicate variation with denatured genomic DNA

**Fig. 6:**
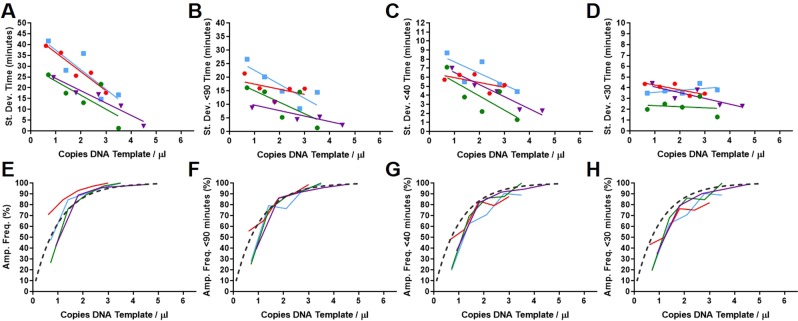
Variance for native and denatured genomic DNA template. Maize event Bt11 (5 percent transgenic to wild type) with 35Sp and NOSt LAMP amplification and SYTO9 detection with calculated copy numbers based on the amplification frequency of the assays (**E to H**). Native and denatured genomic DNA template before LAMP amplification. Red: 35Sp native DNA; Light blue: 35Sp denatured DNA; Purple: NOSt native DNA and Green: NOSt denatured DNA. Variance between replicates calculated from the full 120 minute assay time (**A**) and truncated assay time to 90 minutes (**B**), 40 minutes (**C**) and 30 minutes (**D**) with corresponding amplification frequencies (**E to H**). The black dotted line represents the predicted amplification frequency at each copy number.

In Fig. 4 the variation between replicates at low copy number of native genomic DNA assayed with NOSt LAMP, showed a lower R^2^ than the results with linearised plasmid DNA and 35Sp LAMP. The comparative complexity of native genomic DNA in LAMP amplification initiation may impact to a greater extent on variation between replicates. We investigated the effect of denaturation of genomic DNA before LAMP amplification on variance (Fig. 6). The copy numbers for the native and denatured template LAMP assay results have been normalised to the amplification frequencies.

The standard deviation data for both denatured and native maize event Bt11 LAMP amplified with 35Sp and NOSt primers are shown in Fig. 6A-D with copy numbers calculated based on amplification frequency (Fig. 6E-H). The full data for all datasets shows a close fit between variance values between native and denatured for the 35Sp and for the NOSt LAMP assays. However, there is separation between the variance derived from 35Sp and NOSt LAMP primers (Fig. 6A) regardless of the presentation of the genomic DNA template. Further data truncation of Time (minutes) values from each partition to 40 minutes reduces the range of variance to between 1 and 9 Time (minutes) but there remains an increase in variance with reducing copy number (Fig. 6C). However at the 30 minute cut off, only the native genomic DNA 35Sp and NOSt LAMP assays show an increase in variance with decreasing copy number (Fig. 6D), indicating break down of this approach with excessive truncation.

### LAMP replicate variation without displacement primers

**Fig. 7:**
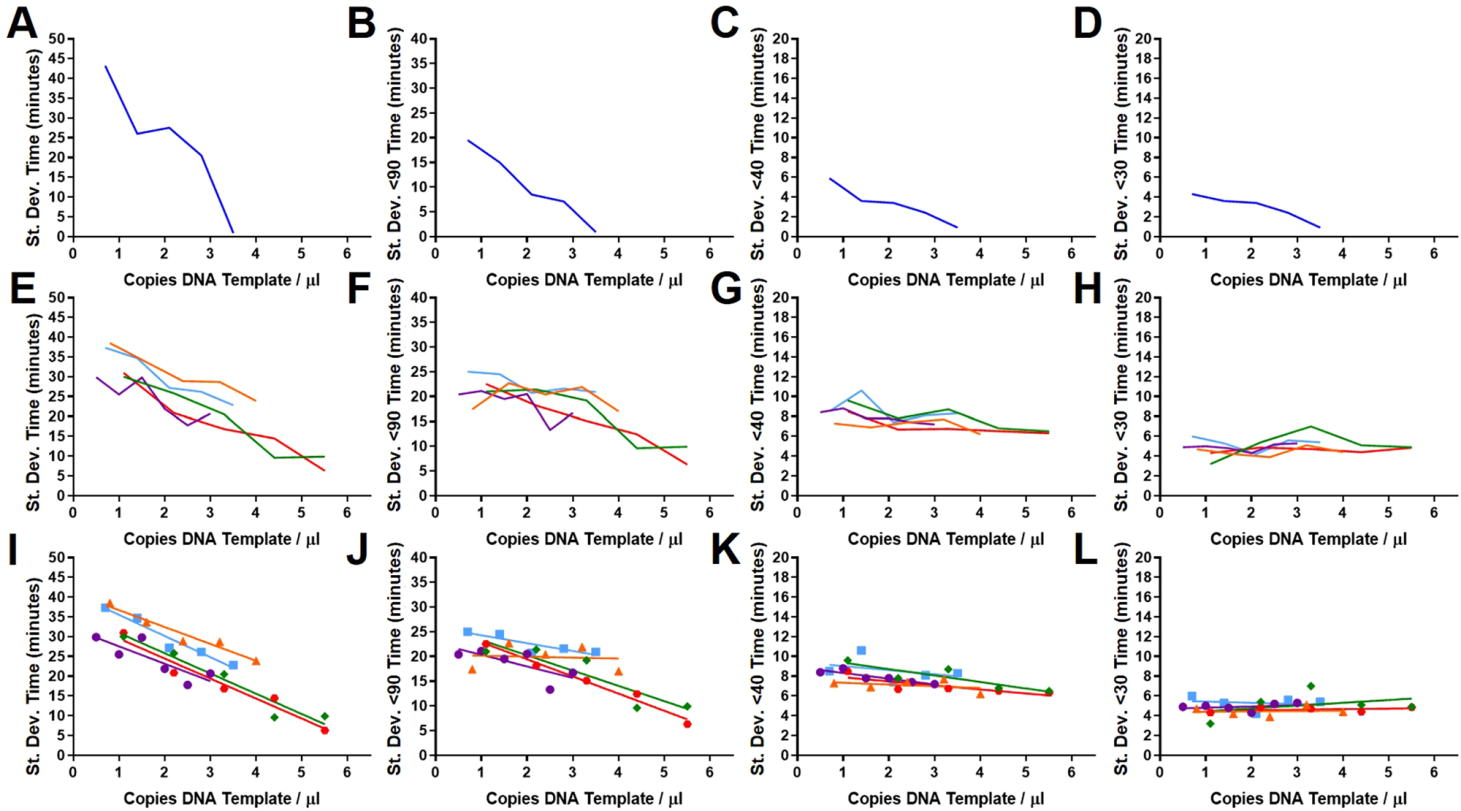
Variance between replicates from LAMP assays without displacement primers. (**A to D**) The variance against normalised copy numbers for the 35Sp LAMP amplification of the 35Sp panhandle artificial template (Table 1) without displacement primers at 120 minutes total assay time (**A**) and truncated to 90 (**B**), 40 (**C**) and 30 minutes (**D**). (**E** to **H**) The variance against normalised copy numbers for 35Sp and NOSt LAMP amplification of linearised plasmid and genomic DNA templates with trendlines below (**I** to **L**) for 120 minutes assays without displacement primers (**E** and **I**, truncated to 90 minutes (**F** and **J**, 40 minutes (**G** and **K** and 30 minutes (**H** and **L**. Dark blue: 35Sp panhandle artificial template; Orange: 35Sp linearised plasmid template; Red: 35Sp native genomic; Light blue: 35Sp denatured genomic; Purple: NOSt native genomic and Green: NOSt denatured genomic DNA.

The initiation of the LAMP reaction requires the invasion of the double stranded DNA target sequence by the LAMP primers and the displacement of the extended strand by the displacement primers. The variation between replicates could therefore be a result of either of these potential time critical steps. This was investigated by using LAMP primers with an oligonucleotide template (Table 1) designed to be initiated for LAMP amplification by one of the LAMP primers without the requirement for displacement primers. This artificial template is similar in structure to the looped amplicons in the cycling stage of LAMP amplification (Fig. 1B). The LAMP amplification should therefore be immediately initiated in the presence of polymerase, artificial template and primers without a possible lag time for the otherwise essential ’breathing’ of double stranded DNA. In Fig. 7, the variance from low copy number artificial template, linearised plasmid and genomic DNA with NOSt and 35Sp without displacement primers is displayed.

**Table 1:** Sequence information for artificial 35Sp and NOSt LAMP templates.

Target, Type, Notation	Template Sequence (5’ to 3’)
35Sp, LAMP, BIP Dumbbell	CCACGTCTTCAAAGCAAGTGGGGATAGTGGGATTGTGCGTCCCCTTACGTCAGTGGACCACTTGCTTTGAAGACGTGGTCTAGATTCCACGATGCTCCTCGGGGGTCCATCTTTGGGCCACTGTCGGCAGAGGCGAGGAGCATCGTGGAA
NOSt, LAMP, FIP Dumbell	CATGCTTAACGTTAATTCAACATGAATCCTGTTGCCGGTCTTGCGATGATTATCATATAATTTTGTTGAATTAACGTTAAGCATGTCTAGAGCATGACGTTATTTATGAGATGATTAGAGTCCCGCAATTATACTAGAAAACAAAATATAGCGCGATCTCATAAATAACGTCATGC

35Sp and NOSt templates synthesised and PAGE purified by IDT (Coralville, Iowa, USA). Both oligonucleotides were diluted to 10 micromolar and stored at minus 20 degrees C. The 35Sp oligonucleotide is 150 bases and the NOSt is 176 bases.

Variation between replicates was still observed with the LAMP amplification of the artificial template (Fig. 7A,B,C,D) for the full data sets and those truncated to 90, 40 and 30 minutes. The variation between replicates reduced rapidly to a low level between 3 and 4 calculated average copies per partition for the amplification of the artificial template. In contrast, standard deviation values above four were observed or extrapolated from the 5 copies per partition (Fig. 7E-H) for the other templates. Although truncation of the results would potentially reduce the impact of late false positives and assays would be quicker, the gradient of the trendlines (Fig. 7I-L) becomes less steep and consequentially less quantifiable using this method.

### Reproducibility of LAMP replicate variation results

**Fig. 8:**
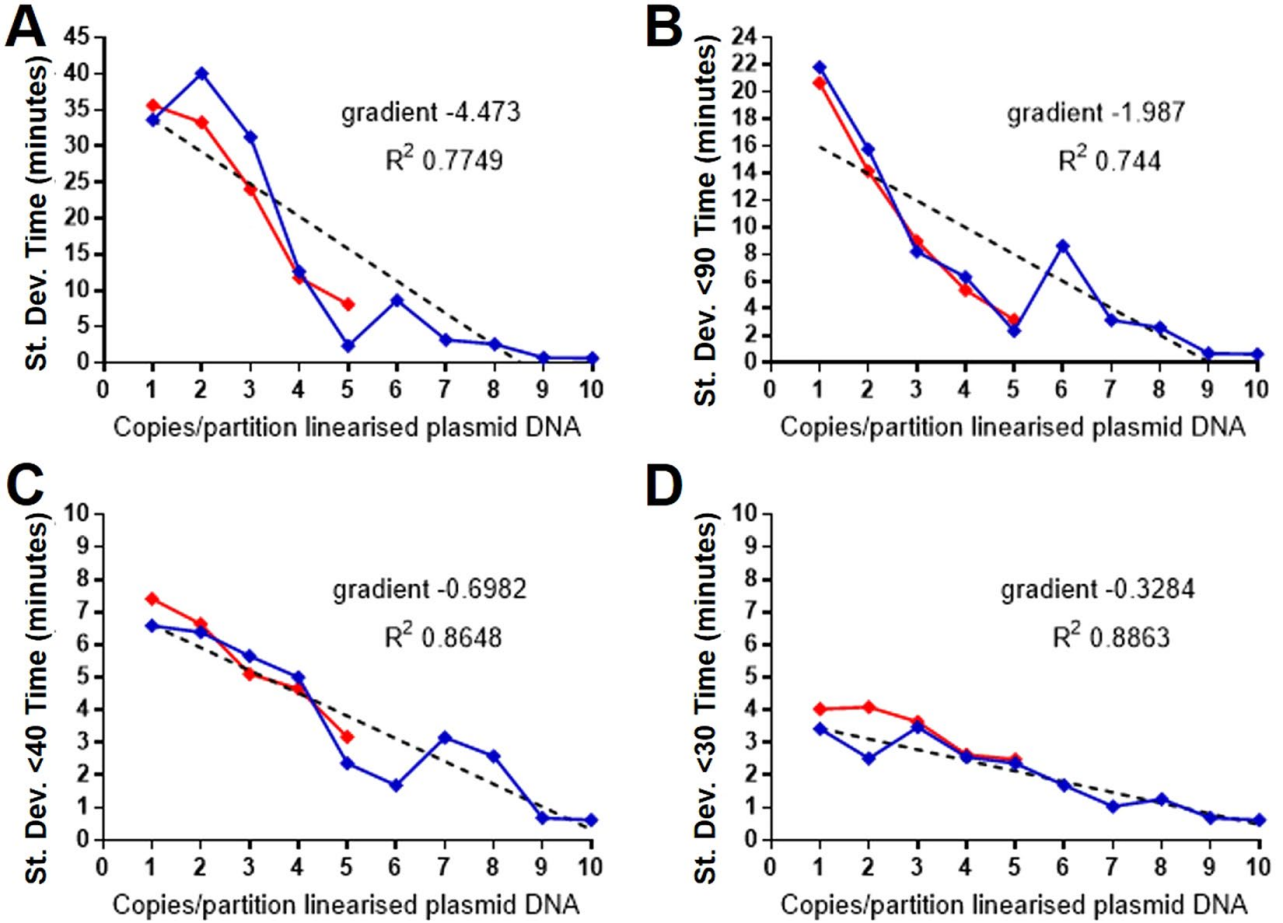
Reproducibility of results with linearised plasmid template. 35Sp LAMP amplification with SYTO9 detection of 1 to 10 copies of linearised plasmid DNA in blue with additional repeat data for 1 to 5 copies in red. Variance for 120 minute assay time and truncated to 90, 40 and 30 minutes with trendline for 1 to 10 copies (dotted line). Full assay time of 120 minutes (**A**), truncated to 90 minutes (**B**), 40 minutes (**C**) and 30 minutes (**D**).

We investigated the reproducibility of the results by combining two sets of 35Sp LAMP data for the same linearised plasmid template. The first set contains individual assays from 1 to 10 copies per partition and the second set contains data from 1 to 5 copies per partition, both detected with the SYTO9 fluorescence method (Fig. 8). The datasets were obtained at different times but used the same primers and template.

The variance from the two datasets show similar data points for the 1 to 5 copies per partition indicating the reproducibility of this method. For the full assay time of 120 minutes and truncated to 90, 40 and 30 minutes this relationship holds. (Alternative metrics are shown for the combined datasets in Supplementary Information: Figs. S32 and S33).

### Multiple templates, LAMP primers and primer combinations

**Fig. 9:**
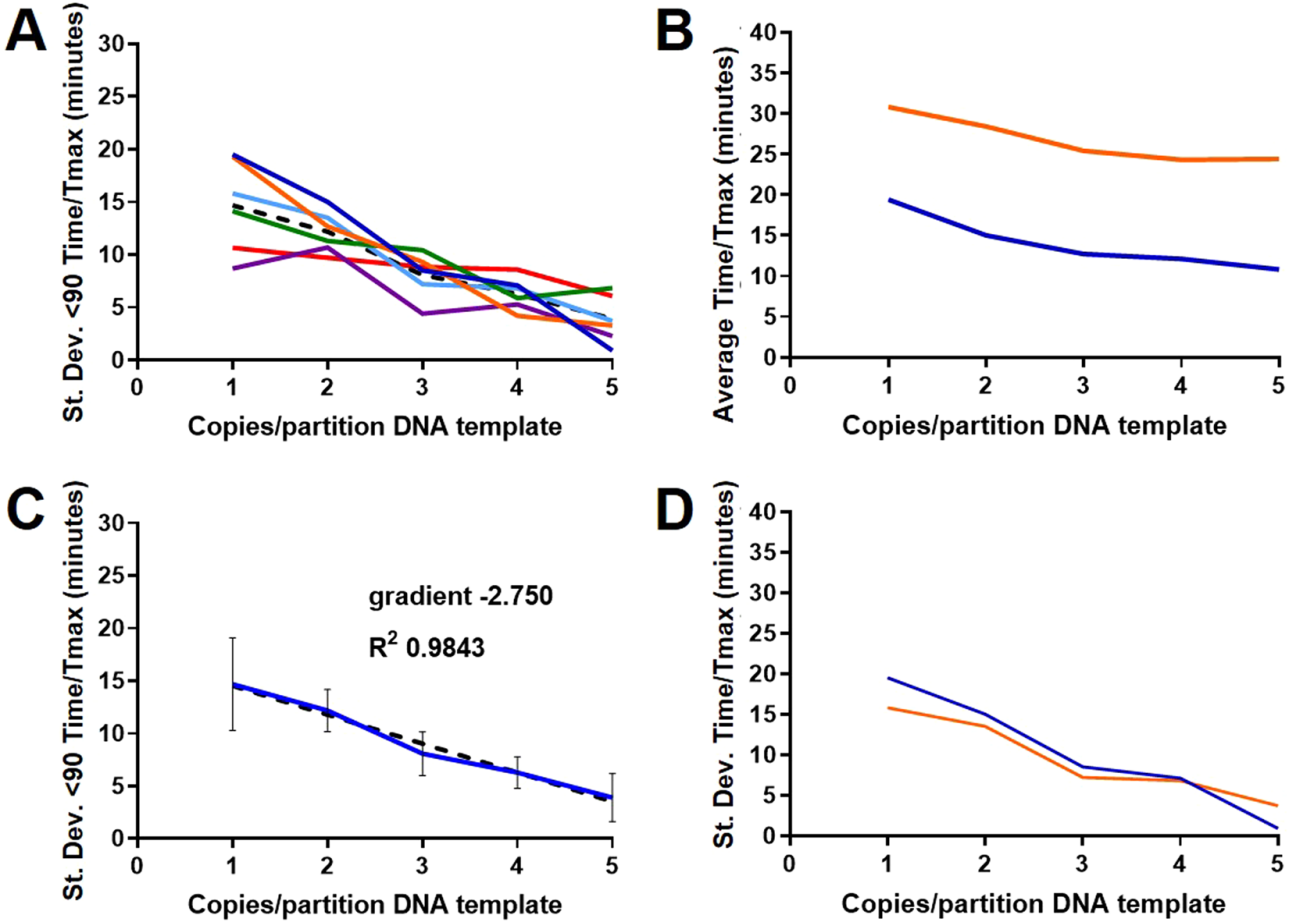
Combined low copy number variation between replicate results from multiple templates, LAMP primer sets and combination of primers. **A** Dark blue: 35Sp artificial template, no displacement primers, SYTO9 detection; Light blue: 35Sp artificial template, no displacement or loop primers, BART detection; Orange: 35Sp plasmid pART7, full primer set, SYTO9 detection; Purple: NOSt native genomic maize, full primer set, SYTO9 detection; Green: Cd28S *Calicophoron daubneyi* genomic DNA, full primer set, BART detection; Red: ADH1 native genomic maize, no displacement or loop primers, BART detection; Black dotted line: mean from the data sets. (**C**) Mean from the data sets with the trendline. (**B** and **D**) Orange: 35Sp LAMP amplification of artificial template without displacement and loop primers, BART detection; Blue: 35S LAMP amplification without displacement primers only, SYTO9 detection. (**B**) Average Ct/Tmax at low copy number and (**D**) Variance between replicates at low copy number.

Finally, we compared variation between replicate results for multiple DNA templates and LAMP assays at low copy number with the omission of displacement and loop primers for selected assays (Fig. 9). LAMP primer sets (Table 2) for 35S promoter, NOS terminator, ADH1 and the 28S ribosomal DNA gene of *Calicophoron daubneyi* were compared for the amplification of genomic, plasmid and oligonucleotide DNA templates. The oligonucleotide artificial template was designed to be amplified from the LAMP cycling stage without requiring displacement primers and was tested with or without loop primers for comparison. The ADH1 LAMP amplification of maize genomic DNA was also conducted in the absence of displacement and loop primers. The calculation of average one copy per partition used the amplification frequency method for all assays with the exception of the linearised plasmid which was diluted from defined copy number stock.

**Table 2:** Oligonucleotide primers for LAMP Primers targetting the 35S promoter, NOS terminator and the ADH1 reference gene have been described previously and are attributed to Guy Kiddle (GK)^19^ and David Lee (DL)^20^.

**Target, Type, Notation**	**Lgth**	**Primer Sequence (5’ to 3’)**
35Sp, Displacer, F3	20	CTTATATAAGAGGAAGGGTCT
35Sp, Displacer, B3(P)	21	GATAAAGGAAAGGC**T**ATC**A**TT
35Sp, Displacer, B3(G)	20	ATAAAGGAAAGGCCATCGTT
35Sp, LAMP, FIP	45	CCACGTCTTCAAAGCAAGTGG-TTTT-GGATAGTGGGATTGTGCGTC
35Sp, LAMP, BIP	37	TTCCACGATGCTCCTCG-TTTT-CCTCTGCCGACAGTGG
35Sp, Loop, LoopF	16	TCCACTGACGTAAGGG
35Sp, Loop, LoopB	16	GGGGTCCATCTTTGGG
NOSt, Displacer, F3	22	CGCGATAATTTATCCTAGTTTG
NOSt, Displacer, B3	19	CGTTCAAACATTTGGCAAT
NOSt, LAMP, FIP	46	GCATGACGTTATTTATGAGA-TTTT-TCGCGCTATATTTTGTTTTCTA
NOSt, LAMP, BIP	43	CATGCTTAACGTAATTCAACA-TTTT-TGAATCCTGTTGCCGGTC
NOSt, Loop, LoopF	22	GATTAGAGTCCCGCAATTATAC
NOSt, Loop, LoopB	23	AAATTATATGATAATCATCGCAA
ADH1, Displacer, F3	19	CTTTGGATCGATTGGTTTC
ADH1, Displacer, B3	17	CCCAAAATTACTCAACG
ADH1, LAMP, FIP	48	CCCTCCGCAAATCTTCGAAC-TTTT-GTAACTGGTGAGGGACTGAG
ADH1, LAMP, BIP	50	GGTGATCAAGTGCAAAGGTC-TTTT-CATAAACCAAGATTAGTCAGATCAAG
ADH1, Loop, LoopF	20	CGCCTTGTTTCTCCTCTGTC
ADH1, Loop, LoopB	16	CAATCCACTCCGAGAC
Cd28S, Displacement, F3	20	CCAAAAAGTCGTGGCTTGGA
Cd28S, Displacement, B3	20	CGGACAGCAATAGCATCTCA
Cd28S, LAMP, FIP	45	GCGGTTGTGTCCGGAGACATTATTTTCAGCTGGCGTGATTTCCTC
Cd28S, LAMP, BIP	44	CTTGCTGGTAGCACAGACGAGGTTTTGCACAACAGCAGAGCGTG
Cd28S, Loop, LoopF	17	TCACGTGGCAACCACA

Cd28S LAMP primers are described here for the first time to target the 28S ribosomal gene of *Calicophoron daubneyi*. All primers are HPLC purified and synthesised by Sigma Aldrich (Poole, UK). The notations (P) and (G) represent primers designed specifically for the pART7 35Sp variant and the CaMV 35Sp sequence in transgenic maize DNA, respectively.

The combination of six low copy number LAMP assays, half detected fluorescently with SYTO9 and the other half detected with the bioluminescent reporter BART, showed remarkable similarities of standard deviation values (Fig. 9A). The average of these values was plotted in Fig. 9C) which shows a coefficient of determination value of 0.9843 and provides the following equation for the relationship between DNA target copy number and the variation between replicates.

$${\rm{Copies}}\ {\rm{of}}\ {\rm{DNA}}\ {\rm{target}}=\frac{{\rm{variance}}\ {\rm{of}}\ {\rm{replicates}}-17.3}{-2.75}$$

The removal of loop primers from the maize genomic DNA assay with ADH1 primers and the amplification of the 35Sp artificial template both continued to show increasing standard deviation with decreasing copy number. Furthermore the comparison between the artificial template assays with and without loop primers showed that the loop primers in this assay are important for faster assay times (Fig. 9B) and that variance is unaffected by the use of loop primers although the speed of the assay is significantly increased in their presence.

## Discussion

The quantification at extremely low concentrations of nucleic acid molecules or molecule counting is challenging. The theoretical limit of detection with the benchmark quantification method qPCR at 95 percent confidence is only 3 copies. Digital PCR requires very large numbers of replicates to differentiate between 3 and 4 copies for accurate quantification. LAMP is a much used isothermal method for quantification, but the linearity of real-time LAMP methods based on reaction times breaks down at extremely low concentrations of target molecules. We observed this with average Time (minutes)/Tmax values below a calculated average of 10 copies per partition with increasing standard deviation with decreasing copy number. Variation in LAMP reaction conditions such as temperature, can have an affect on the rate of the amplification reaction and therefore this is a limitation of metrics that are reliant on timings.

We show that the variation between replicates is a reproducible and inherent feature of LAMP amplification at low copy number. This variation was observed with a range of templates from genomic DNA extracts to synthesised oligonucleotides, LAMP primer sets, amplification detection strategies and template presentation. The variation between replicates was not observed with PCR confirming it as specific to LAMP. Increasing variation was observed with decreasing copy number and the method could be used to to separate individual average copy numbers for example 4 copies per partition from 5 copies per partition which would require a large number of replicates for digital PCR.

The 35S promoter LAMP primer set had a greater propensity for false positives than the NOS terminator primers and we showed that the low copy number variance could be used with either detection of true amplicons using a fluorescence quenching assay or time truncated assay results, but overly truncated results reduce this relationship. Interestingly from the denatured/native genomic DNA template experiments there was separation between the results for 35Sp and NOSt LAMP primers with both presentations of the same template, indicating that the degree of variation between replicates is dependent on LAMP primers. The strand invasion to initiate LAMP amplification may be less favourable for the 35Sp LAMP primers adding to the variation observed. Alternatively the 35Sp LAMP primers may amplify less efficiently. It was clear that the displacement primers could be omitted and variance would still be observed at low copy number. Together these results indicate that the initial invasion and/or extension by the looped FIP and BIP LAMP primers is a rate limiting variable.

The removal of both displacement primers and loop primers leaving only the hairpin forming FIP and BIP primers resulted in similar variance between replicates at low copy number compard to assays with a full complement of primers. This indicates that the variation observed is due to the FIP and BIP primers and suggests that time delays in the initiation of amplification by these primers results in a wide range of positive partition Time (minutes)/Tmax times, which is more evident at low copy number. The assays at one copy per partition describes an average of a single copy per partition, the Poisson distribution of these copy numbers shows that statistically an individual partition could contain between zero and four or more copies but with the highest frequency at one copy in a partition. A delay in the initiation of the single copy in a partition will produce a more varied result than multiple copies in a partition, for example a small number of rapidly initiated copies could mask the effect of slowly initiated copies in the same partition. The Poisson distributions of four and five average copies per partition are likely to have a large number of partitions with the same number of copies in them, for examples three, four, five and six copies, but remarkably this slight shift in distribution is sufficient to enable the variance between replicates to be used to separate these template concentrations. At an average of ten copies per partition it becomes less likely that there will be zero copies in a partition and hence the amplification frequency will increase to 100 percent, but single or low copy number partitions could provide variation between replicates from delayed initiation of amplification. Higher template concentrations will result in the minimum number of copies in a partition increasing further and the variation from these partitions will reduce resulting in average Time (minutes)/Tmax values with low standard deviation.

The variance between replicates method bridges the gap between the linearity of time to peak LAMP quantification for low to high template concentrations with digital LAMP for dilute samples. This combination of methods provides the possibility of full dynamic range quantification in a closed tube format without the need for sample dilution.

## Methods

### DNA templates

#### Linearised plasmid

Linearised plasmid DNA was re-suspended from aliquots of a stock of quantified pART7^14^ lyophilised with carrier DNA as previously described^15^. The defined copy number concentration was calculated from initial quantification by NanoDrop spectrophotometry and Agilent Bioanalyzer. The plasmid contains a variant of the 35S promoter sequence to which LAMP primers have been adapted. For calculations of copy number, plasmid length was assumed to be 4.9kb. Multiple aliquots of the same stock were stored at minus 20 degrees C and for each experiment an aliquot was reconstituted.

#### Artificial templates

The artificial LAMP templates were designed to be targeted initially by the FIP or BIP primers to start the cycling phase of LAMP amplification without the requirement of target sequences for displacement primers. The templates were synthesised by Integrated DNA Technologies (IDT, Iowa, United States) with PAGE purification (Table 1). Artificial templates were designed for 35Sp and NOSt LAMP primers including target sequences for the Loop primers.

#### Genomic

Two *Zea mays* genomic DNA templates were extracted for assay use. A 5 percent defined fraction certified reference material of maize transgenic event Bt11 (Fluka GmbH, Buchs, Switzerland) and maize event Mon810, were extracted with the Promega Wizard genomic DNA purification kit (Madison, United States) following the manufacturer’s instructions for plant tissue with 50 microlitres of rehydration buffer and storage at 4 degrees C. Bt11 contains two copies of the 35S promoter sequence from the cauliflower mosaic virus (35Sp) and nopaline synthase terminator (NOSt) from *Agrobacterium tumefaciens*^16^. Mon810 contains one copy of the 35S promoter sequence and the NOSt sequence is absent^17,18^. A further DNA template was extracted from the rumen fluke *Calicophoron daubneyi* by phenol:chloroform:isoamyl alcohol and purified with a NucleoSpin gDNA clean-up kit (Macherey-Nagel, Düren, Germany).

### Denaturing genomic DNA

The genomic DNA template was heat treated at 95 degrees C for 5 minutes before transferring to ice for 5 minutes for experiments using denatured genomic DNA.

### Copy number calculations

#### Calculator

Calculating copy number of target sequences from concentrations using the formula previously described in Hardinge et al 2018^15^:

$${\rm{Copies}}\,{\rm{of}}\,{\rm{target}}=\frac{{\rm{ng}}\,{\rm{of}}\,{\rm{double}}\,{\rm{stranded}}\,{\rm{DNA}}\times {\rm{Avogadro}}\mbox{'}{\rm{s}}\,{\rm{constant}}(6.022\times 1{0}^{23})}{{\rm{Length}}\,{\rm{in}}\,{\rm{base}}\,{\rm{pairs}}\times 1{0}^{9}\times 650\,{\rm{Daltons}}}$$

The size of the linearised plasmid pART7 was assumed to be 4900 base pairs and 2.4 × 10^9^ base pairs was used in calculations for the maize genome.

The online calculator was used at https://cels.uri.edu/gsc/cndna.html.

#### Transgenic genomic template

Calculations for genomic DNA and transgenic GM maize reference materials follow the guidelines described in Hardinge et al 2018^15^.

#### Amplification frequency

Using digital PCR statistics to calculate copy numbers based on amplification frequency from multiple replicates, for example 63 percent positive partitions quantified as 1 copy per partition. Therefore normalisation of variation between replicates and other metrics was to the amplification frequency calculation of copy number. The dilution of template based on normalisation was confirmed with LAMP-BART assays (Supplementary Information; Figs. S34 and S35).

### DNA quantification

#### Spectrophotometry

Initial quantification of the template DNA by NanoDrop 1000 (Fisher Scientific, UK) spectrophotometer followed the manufacturer’s guidelines for double stranded DNA as described^15^. The extracts were assessed for purity using the NanoDrop using the wavelength ratios 260:280 nm and 260:230 nm. Values between 1.7 and 1.9 for the ratio 260:280 were deemed acceptable; as were those between 2.0 and 2.2 for the ratio 260:230.

#### Fluorimetry

DNA concentration results were achieved using Qubit (Life Technologies, UK) fluorometry. The operation of the Qubit version 2.0 fluorometer and use of the dsDNA BR assay kit followed the manufacturer’s guidelines for quantification of double stranded DNA.

#### Agilent quantification

The Agilent Bioanalyser (Agilent; California, USA) was used to initially quantify the linearised plasmid pART7. Agilent Tapestation genomic tapes were used to quantify maize genomic DNA from the events Bt11 and Mon810, following the protocol for genomic tapes as described^15^. Briefly 1 microlitre of either genomic DNA or ladder was combined with 10 microlitres of genomic DNA sample buffer, vortexed and loaded into the instrument. The genomic tapes were robotically loaded and the resulting bands sized between 200 and greater than 60000 base pairs.

### Oligonucleotide primers

#### LAMP primers

All oligonucleotide primers were synthesised by Sigma with HPLC level purification. The LAMP primer set targeting the 35S promoter has an alternative displacement primer designed for the plasmid pART7. LAMP primer set for NOS terminator sequences and for ADH1 reference gene were also used (Table 2). Furthermore, LAMP primers targeting the 28S ribosomal gene of *Calicophoron daubneyi* designed using Primer Explorer v5 (Eiken, Japan) were tested.

#### LAMP quenched fluorescent primers

Fluorescently labelled quenched primers^13^ were synthesised by Sigma with the fluorophore attached to the 5’ end of the oligonucleotide without further adaptation. JOE (6-carboxy-4’,5’-dichloro-2’,7’-dimethoxyfluorescein) selected for detection on the yellow channel of a Qiagen (Hilden, Germany) RotorGene thermal cycler. Labelled FIP LAMP primers for 35Sp and NOSt purified to the HPLC level (Table 3).

**Table 3:** LAMP quenched fluorescent primers Modified 35S promoter and NOS terminator FIP primers with JOE (6-carboxy-4’,5’-dichloro-2’,7’-dimethoxyfluorescein) attached to the 5’ end^13^.

Target, Type, Notation	Lgth	Primer Sequence (5’ to 3’)
35Sp, LAMP, **JOE**-FIP	45	[**JOE**]CCACGTCTTCAAAGCAAGTGG-TTTT-GGATAGTGGGATTGTGCGTC
NOSt, LAMP, **JOE**-FIP	46	[**JOE**]GCATGACGTTATTTATGAGA-TTTT-TCGCGCTATATTTTGTTTTCTA

The quenched fluorescent LAMP primer was synthesised and HPLC purified by Sigma Aldrich (Poole, UK).

#### PCR primers

PCR oligonucleotides were synthesised by Sigma with HPLC purification (Table 4).

**Table 4:** PCR Primers.

Target, Type, Notation, Version	Length	Primer Sequence (5’ to 3’)
35Sp, PCR, Forward, **M3**	21	CGTCTTCAAAGCAAGTGGATT
35Sp, PCR, Reverse, **M3**	22	TCTTGCGAAGGATAGTGGGATT

Quantitative PCR primers which target the CaMV 35S promoter sequence. Version M3 denotes primers designed by Fernandez et al. 2005^21^. HPLC grade purified primers were used unless specified.

### LAMP amplification and bioluminescence detection

All reagents were supplied by Sigma (Missouri, United States) unless otherwise stated. The LAMP with bioluminescent assay in real-time (BART) reaction chemistry has previously been described by Gandelman et al 2010^12^. LAMP BART reactions contained 1X isothermal buffer (New England Biolabs Inc, Massachusetts, United States), 100 nanograms per partition salmon sperm carrier DNA, 60 millimolar potassium chloride (KCl), 0.4 milligrams per millilitre polyvinylpyrrolidone (PVP), 10 millimolar dithiothrietol (DTT), 300 micromolar of each deoxynucleotide triphosphate (dNTP), 100 micrograms per millilitre D-luciferin (Europa, Ipswich, United Kingdom), 250 micromolar adenosine-5’-O-phosphosulfate (APS; Biolog, Bremen, Germany), 5.5 micrograms per millilitre Ultra-Glo luciferase (Promega, Madison, United States), 0.32 units per microlitre Bst polymerase v2.0 warm start (NEB), 0.375 units per microlitre ATP sulfurylase (NEB), 0.2 micromolar each displacement primer, 0.4 micromolar each Loop primer, 0.8 micromolar each LAMP primer and molecular grade water for a reaction volume of 10 microlitres. For the reactions without displacement primers molecular grade water was substituted. Mineral oil was added to each LAMP-BART partition with a clear adhesive film to seal. The reaction was set for 90 minutes at 60 degrees C with 1 minute acquisition integrals unless otherwise stated. Bioluminescence detection and analysis used React IVD software (Synoptics, Cambridge, United Kingdom) in a ‘Lucy’ device developed by ERBA MDX (Ely, United Kingdom).

### LAMP amplification with fluorescence detection

LAMP amplification of DNA samples was detected in real time using the dye SYTO 9 Green (Life Technologies, Eugene, United States) on a Qiagen (Hilden, Germany) RotorGene thermal cycler as previously described by Hardinge et al 2018^15^. Reagents, unless otherwise stated, were supplied by Sigma Aldrich (Poole, UK). A total reaction volume of 10 microlitres contained 1X isothermal buffer (NEB), 0.2 micromolar each displacement primer, 0.4 micromolar each Loop primer, 0.8 micromolar each LAMP primer, 300 micromolar each dNTP, 0.8 micromolar Betaine, 0.32 units per microlitre Bst polymerase v2.0 warm start (NEB), 0.5 micromolar SYTO 9 Green (ThermoFisher Scientific, Massachusetts, United States) and molecular grade water. For the reactions without displacement primers molecular grade water was substituted. The reaction conditions on the thermocycler were 90 cycles of 1 minute at 60 degrees C unless otherwise stated (i.e. a constant 60 degrees C for 90 minutes). Acquisition of fluorescence was on the green channel and melt temperature analysis was derived from data between 60 and 95 degrees C. The results were analysed with RotorGene 6000 software v1.7, TeeChart Office and Microsoft Excel.

### LAMP amplification with quenched fluorescence LAMP primer detection

The LAMP detection method using quenched fluorescent primers was previously described by Hardinge et al 2019^13^. Briefly, oligonucleotide LAMP FIP primers with the JOE fluorophore attached to the 5 prime end were incorporated into the fluorescent LAMP amplification method with the inclusion of SYTO9 for dual detection SYTO9^13^. The LAMP reaction was monitored in real time on the RotorGene thermocycler according to the emission wavelength of the fluorescence, SYTO9 using the green channel (source 470 +/- 10 nanometers, detection 510 +/- 5 nanometers) and JOE on the yellow channel (source 530 +/- 5 nanometers, detection 557 +/- 5 nanometers) in the same reaction. Melt temperature analysis followed amplification between 60 and 95 degrees C. Results were analysed with RotorGene 6000 software v1.7 and Microsoft Excel.

### Random number generation and data analysis

Number sequence generated at RANDOM.ORG with timestamp: 2019-05-29 14:43:59 UTC for integers up to 144.

105, 100, 59, 68, 136, 35, 34, 131, 97, 67, 84, 50, 99, 37, 118, 2, 15, 89, 73, 82, 92, 134, 103, 32, 39, 102, 31, 144, 79, 17, 36, 122, 98, 54, 85, 112, 16, 140, 104, 66, 90, 95, 12, 139, 137, 64, 113, 46, 143, 88, 23, 76, 110, 123, 63, 53, 114, 83, 117, 28, 94, 43, 51, 121, 125, 141, 132, 9, 93, 142, 135, 18, 120, 80, 42, 38, 77, 116, 78, 126, 26, 10, 24, 52, 57, 129, 4, 41, 3, 119, 127, 62, 74, 96, 71, 109, 21, 72, 13, 81, 128, 101, 11, 133, 20, 86, 61, 65, 91, 19, 30, 8, 108, 115, 40, 49, 33, 111, 130, 7, 29, 25, 69, 55, 5, 44, 87, 58, 70, 1, 75, 48, 14, 47, 60, 45, 6, 27, 56, 138, 106, 107, 22, 124.

Partitions were labelled from 1 to 144 and partition number 1 was analysed first. For the randomised data analysis partition number 105 was analysed first followed by number 100 and so on.

Data was analysed with Microsoft Excel and graphically represented using Graphpad Prism 8.1.2.

## Supplementary information

Supplementary Information.
