# Supplementary material for: Full Dynamic Range Quantification using Loop-mediated Amplification (LAMP) by Combining Analysis of Amplification Timing and Variance between Replicates at Low Copy Number
Source: Sci Rep. 2020 Jan 22;10:916. doi: 10.1038/s41598-020-57473-1 (PMC6976701; doi:10.1038/s41598-020-57473-1)
Supplement: Supplementary file 1 — Supplementary Information. [file 41598_2020_57473_MOESM1_ESM.docx]

Supplementary Information

**Full Dynamic Range Quantification using Loop-mediated Amplification (LAMP) By Combining Analysis of Amplification Timing and Variance between Replicates at Low Copy Number**

**Patrick Hardinge^1,*^, James A. H. Murray^1^**

^1^Cardiff School of Biosciences, Biomedical Sciences Building, Museum Avenue, Cardiff CF10 3AX, UK

[*hardingep@cardiff.ac.uk](mailto:*hardingep@cardiff.ac.uk)


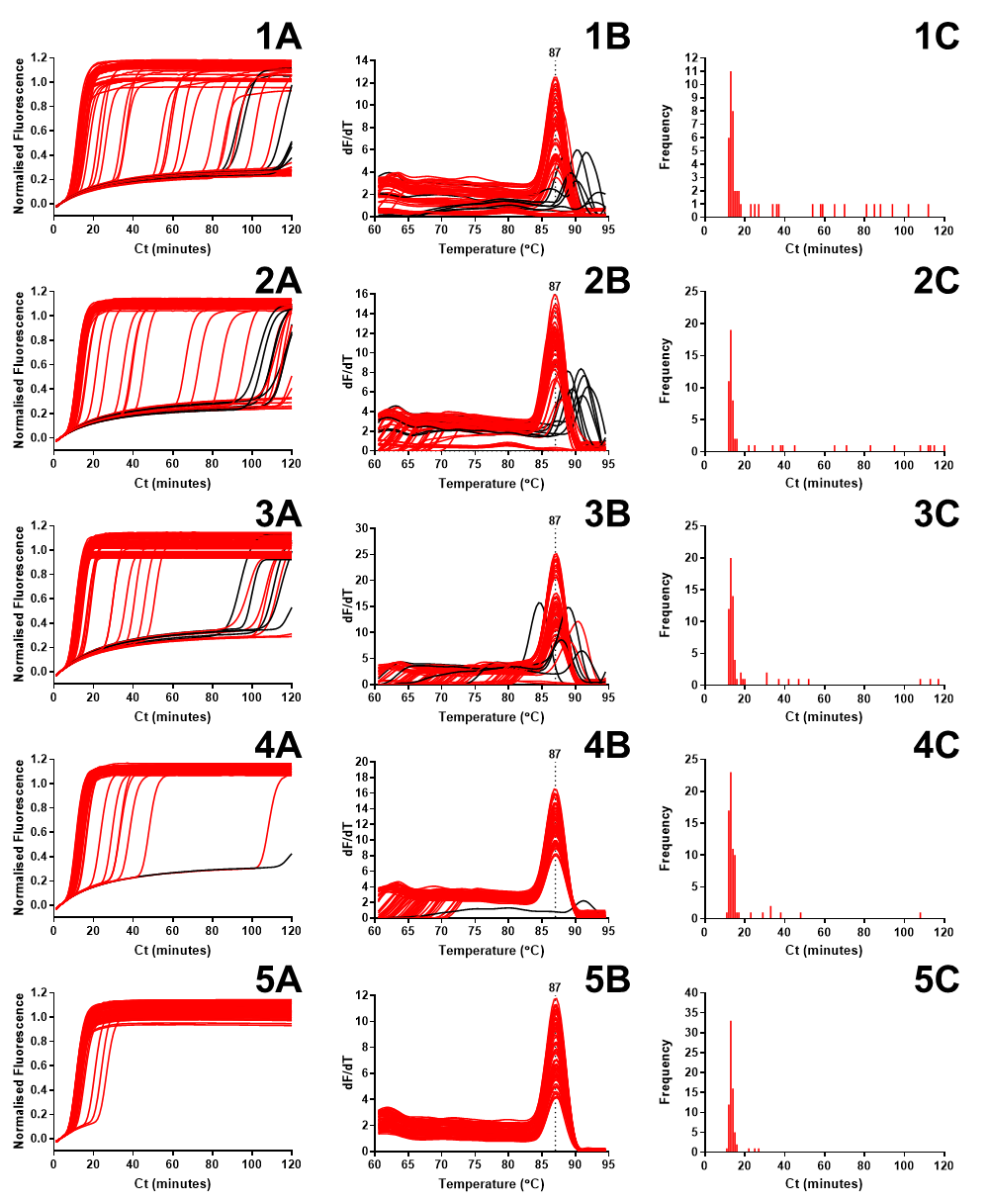


**Figure S1: Variation between replicates and amplification frequency at low copy number #1.** 35Sp primers, pART7 plasmid DNA template, 72 replicates per assay, fluorescent detection with SYTO9 **(1)** 1 copy of linearised plasmid per reaction, **(2)** 2 copies per reaction, **(3)** 3 copies per reaction, **(4)** 4 copies per reaction and **(5)** 5 copies per reaction. **(A)** cycles of 1 minute at 60 degrees C against normalised fluorescence for 72 replicates, **(B)** melt curve analysis and **(C)** frequency distribution of positive reactions. Possible false positive results in black.


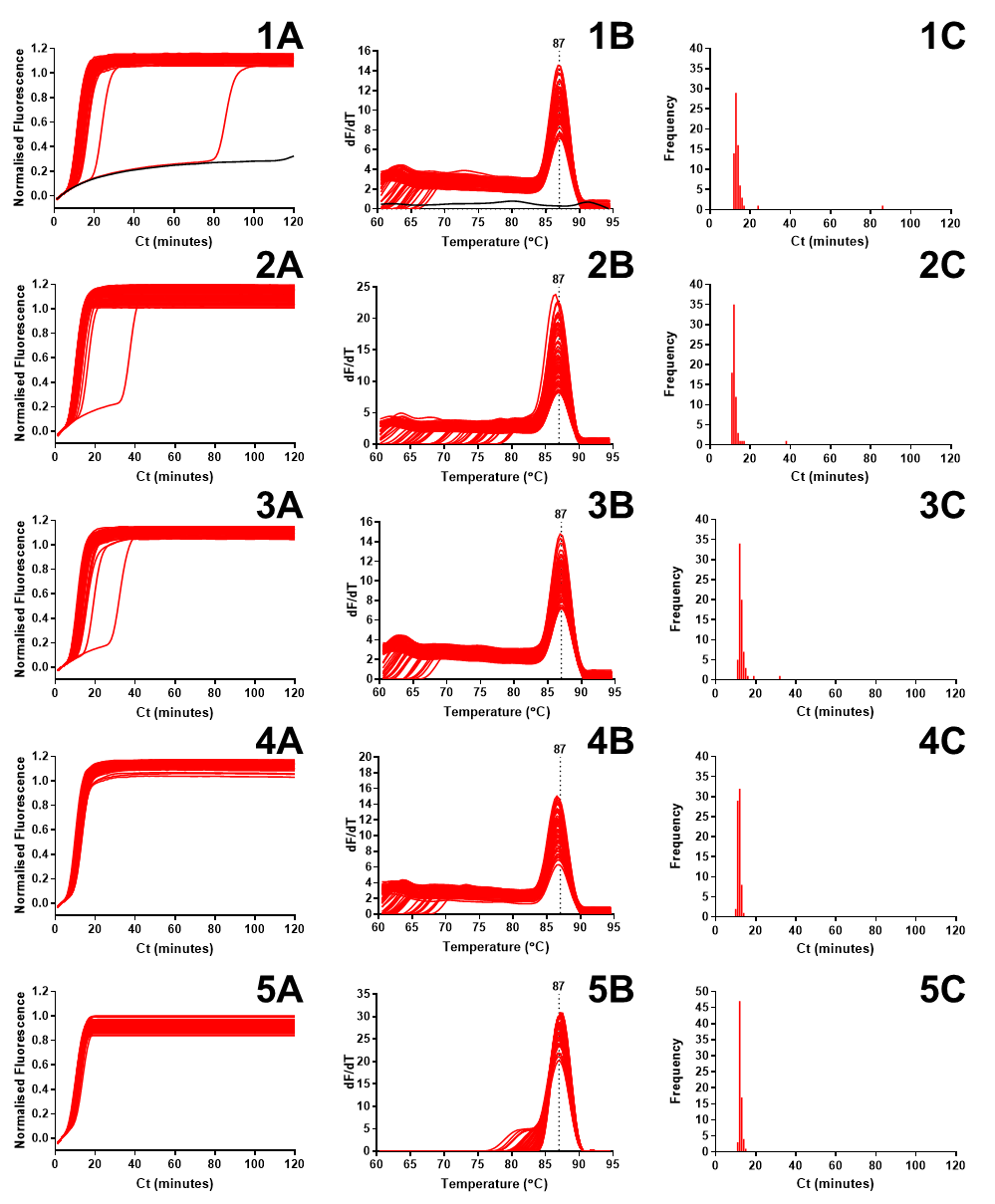


**Figure S2: Variation between replicates and amplification frequency at low copy number #2.** 35Sp primers, pART7 plasmid DNA template, 72 replicates per assay, fluorescent detection with SYTO9 **(1)** 6 copies of linearised plasmid per reaction, **(2)** 7 copies per reaction, **(3)** 8 copies per reaction, **(4)** 9 copies per reaction and **(5)** 10 copies per reaction. **(A)** cycles of 1 minute at 60 degrees C against normalised fluorescence for 72 replicates, **(B)** melt curve analysis and **(C)** frequency distribution of positive reactions. Possible false positive results in black.


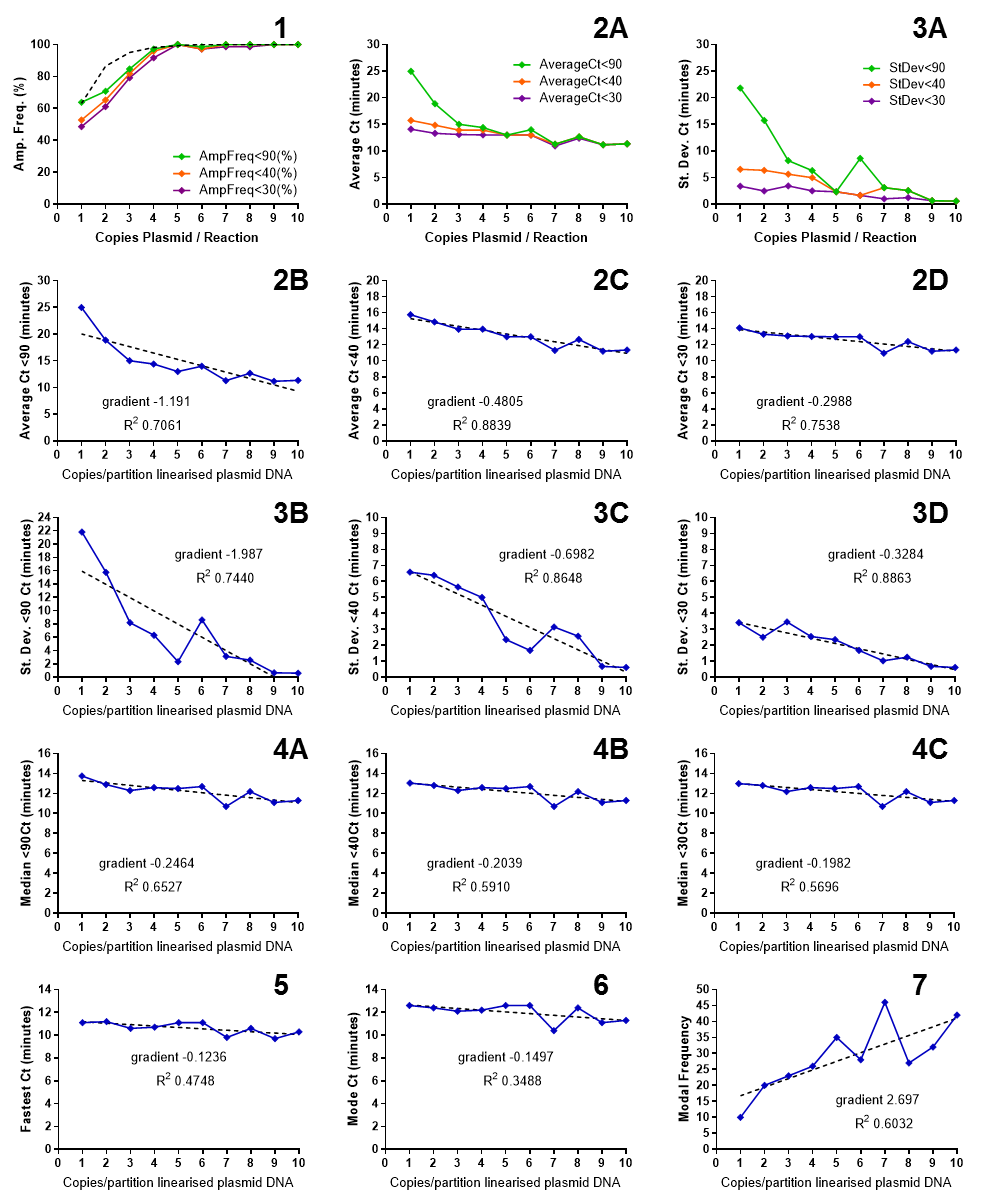


**Figure S3: Variation between replicates and amplification frequency at low copy number #3.** LAMP 35Sp amplification of linearised plasmid. **(1)** amplification frequency from 1 to 10 copies per partition for truncated data compared to the predicted amplification frequency (dotted line), **(2)** average Ct for 90, 40 and 30 minute assay time, **(3)** variance for assay results less than 90, 40 and 30 Ct, **(2B)** average Ct <90 minutes, **(2C)** average Ct <40 minutes, **(2D)** average Ct <30 minutes, **(3B)** variance <90 minutes, **(3C)** variance <40 minutes, **(3D)** variance <30 minutes, **(4A)** median Ct <90 minutes, **(4B)** median Ct <40 minutes, **(4C)** median Ct <30 minutes, **(5)** fastest Ct, **(6)** mode Ct and **(7)** modal frequency (the number of replicates comprising the mode Ct).


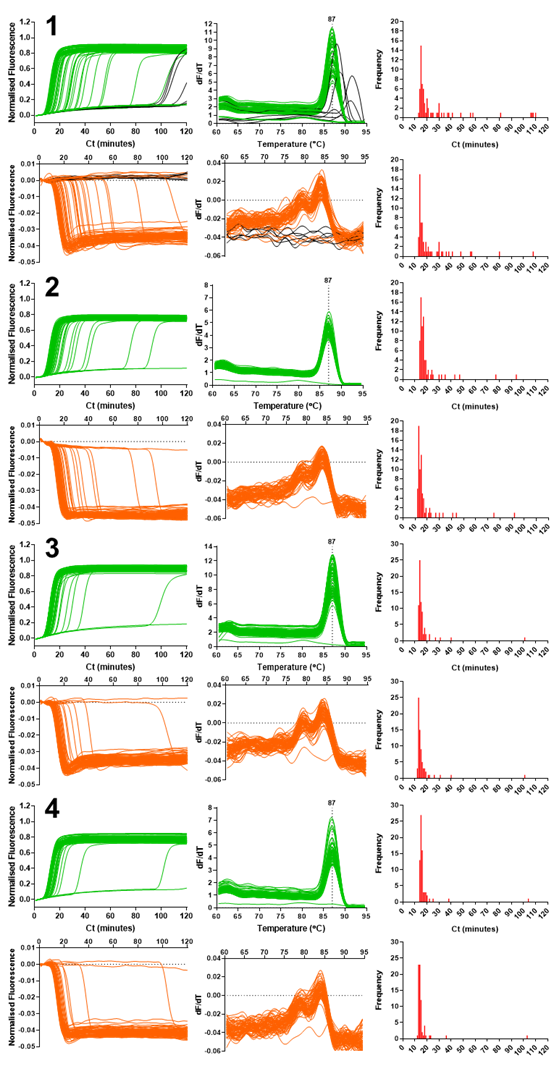


**Figure S4: SYTO9/JOE-FIP dual detection for false positives from 35Sp and NOSt LAMP primers #1.** LAMP 35Sp amplification of linearised plasmid, 72 replicates per assay. **(1)** 2 copies per partition, **(2)** 3 copies per partition, **(3)** 4 copies per partition and **(4)** 5 copies per partition. SYTO9 detection in green, JOE-FIP detection of the same reaction in orange. Frequency distribution of results in red.


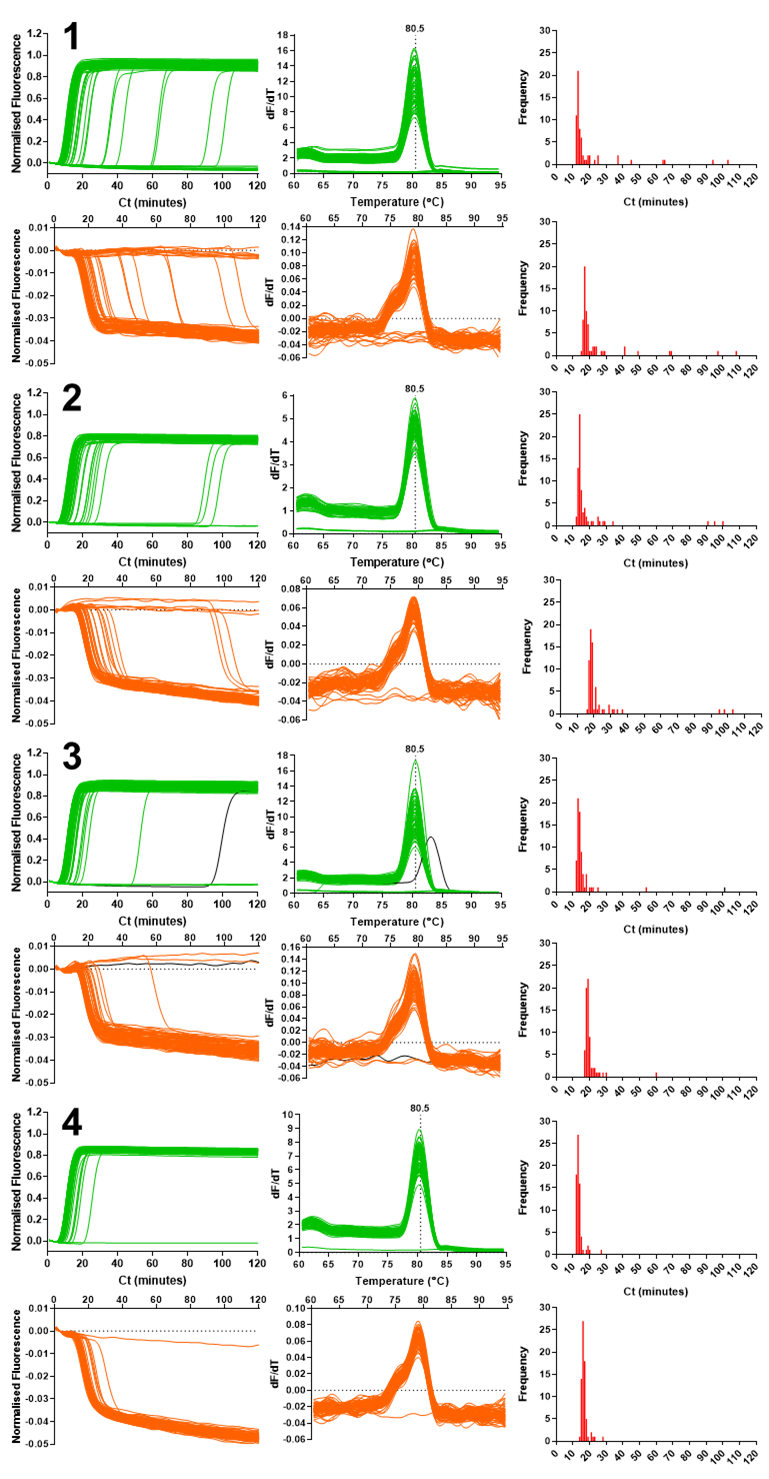


**Figure S5: SYTO9/JOE-FIP dual detection for false positives from 35Sp and NOSt LAMP primers #2.** LAMP NOSt amplification of 5%Bt11 genomic maize DNA, 72 replicates per assay. **(1)** 2 copies per partition, **(2)** 3 copies per partition, **(3)** 4 copies per partition and **(4)** 5 copies per partition. SYTO9 detection in green, JOE-FIP detection of same reaction in orange. Frequency distribution of results in red.


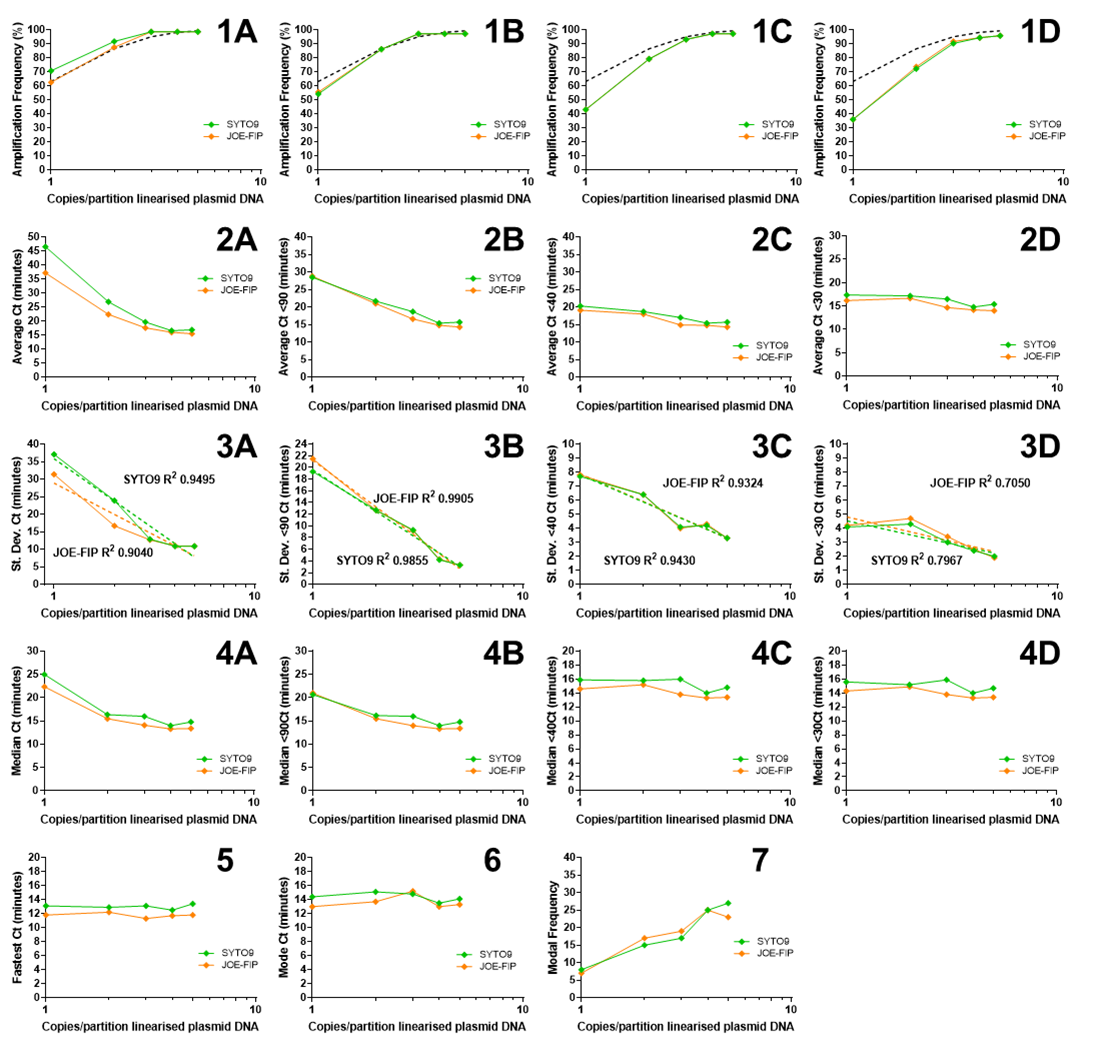


**Figure S6: SYTO9/JOE-FIP dual detection for false positives from 35Sp and NOSt LAMP primers #3.** LAMP 35Sp amplification of linearised plasmid. SYTO9 results in green and JOE-FIP in orange. **(A,B,C,D)** Full assay time and truncated to 90, 40 and 30 minutes. **(1)** Amplification frequency, **(2)** average Ct, **(3)** standard deviation, **(4)** median Ct, **(5)** fastest Ct, **(6)** mode Ct and **(7)** frequency of the mode (the number of replicates comprising the mode Ct).


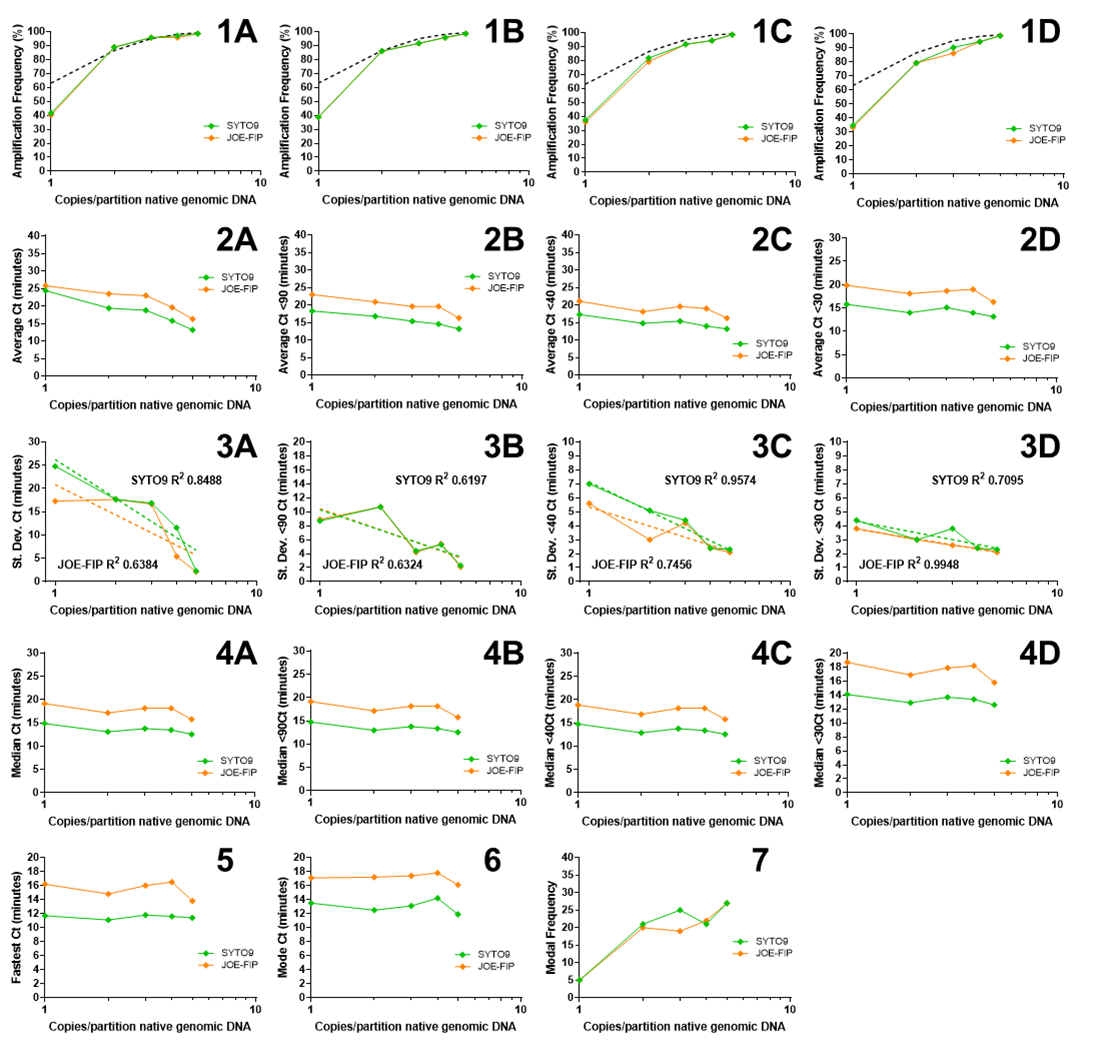


**Figure S7: SYTO9/JOE-FIP dual detection for false positives from 35Sp and NOSt LAMP primers #4.** LAMP NOSt amplification of 5%Bt11 genomic maize DNA. SYTO9 results in green and JOE-FIP in orange. **(A,B,C,D)** Full assay time and truncated to 90, 40 and 30 minutes. **(1)** Amplification frequency, **(2)** average Ct, **(3)** standard deviation, **(4)** median Ct, **(5)** fastest Ct, **(6)** mode Ct and **(7)** frequency of the mode (the number of replicates comprising the mode Ct).


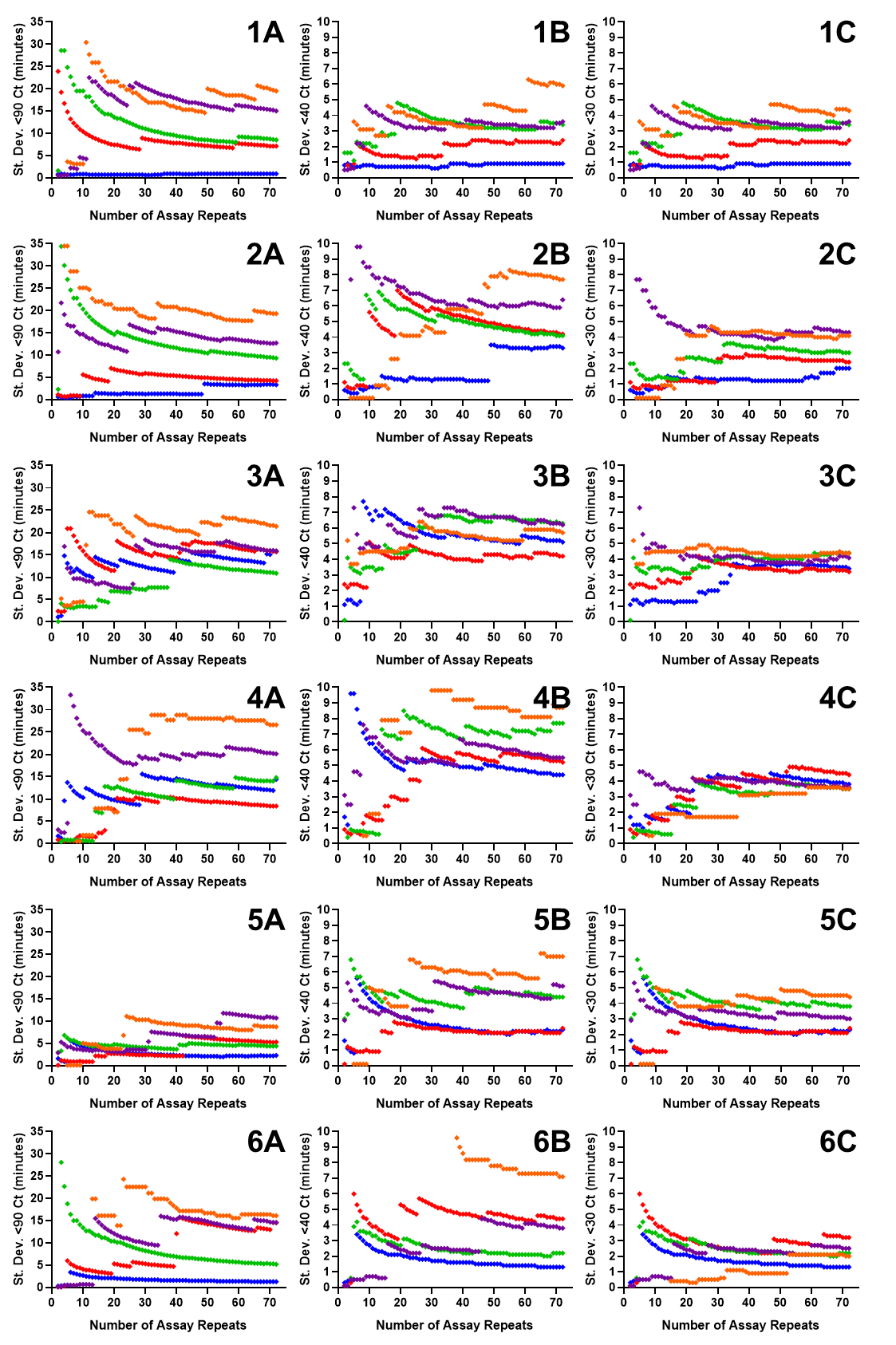


**Figure S8. Randomised replicate Ct values to show separation between 1 and 5 copies with increasing number of replicates #1.** The order in which the data was analysed was randomised by using the new order of partitions, and the variance calculated from increasing number of assay replicates for various templates and LAMP primer assays for 1 to 5 copies per reaction. Total assay replicates of 72, assay time truncated to 90 minutes **(A)**, 40 minutes **(B)** and 30 minutes **(C)**. Orange: 1 copy; Purple: 2 copies; Green: 3 copies; Red: 4 copies and Blue: 5 copies. **(1)** 35Sp artificial template with 35Sp LAMP primers, **(2)** pART7 linearised plasmid with 35Sp primers, **(3)** native genomic DNA (5% Bt11) with 35Sp primers, **(4)** denatured genomic DNA with 35Sp primers, **(5)** native genomic DNA with NOSt LAMP primers and **(6)** denatured genomic DNA (5% Bt11) with NOSt primers.

**RANDOM.ORG Random Sequence Generator** with timestamp: 2019-03-11 10:37:33 UTC

31,43,70,64,56,50,42,68,30,24,39,16,27,52,55,44,21,38,19,36,17,63,47,13,46,66,62,57,35,65,23,20,22,6,25,4,9,60,41,58,32,51,26,40,2,59,49,8,48,67,29,69,15,1,34,7,5,12,72,11,54,33,37,10,18,14,61,53,45,3,71,28


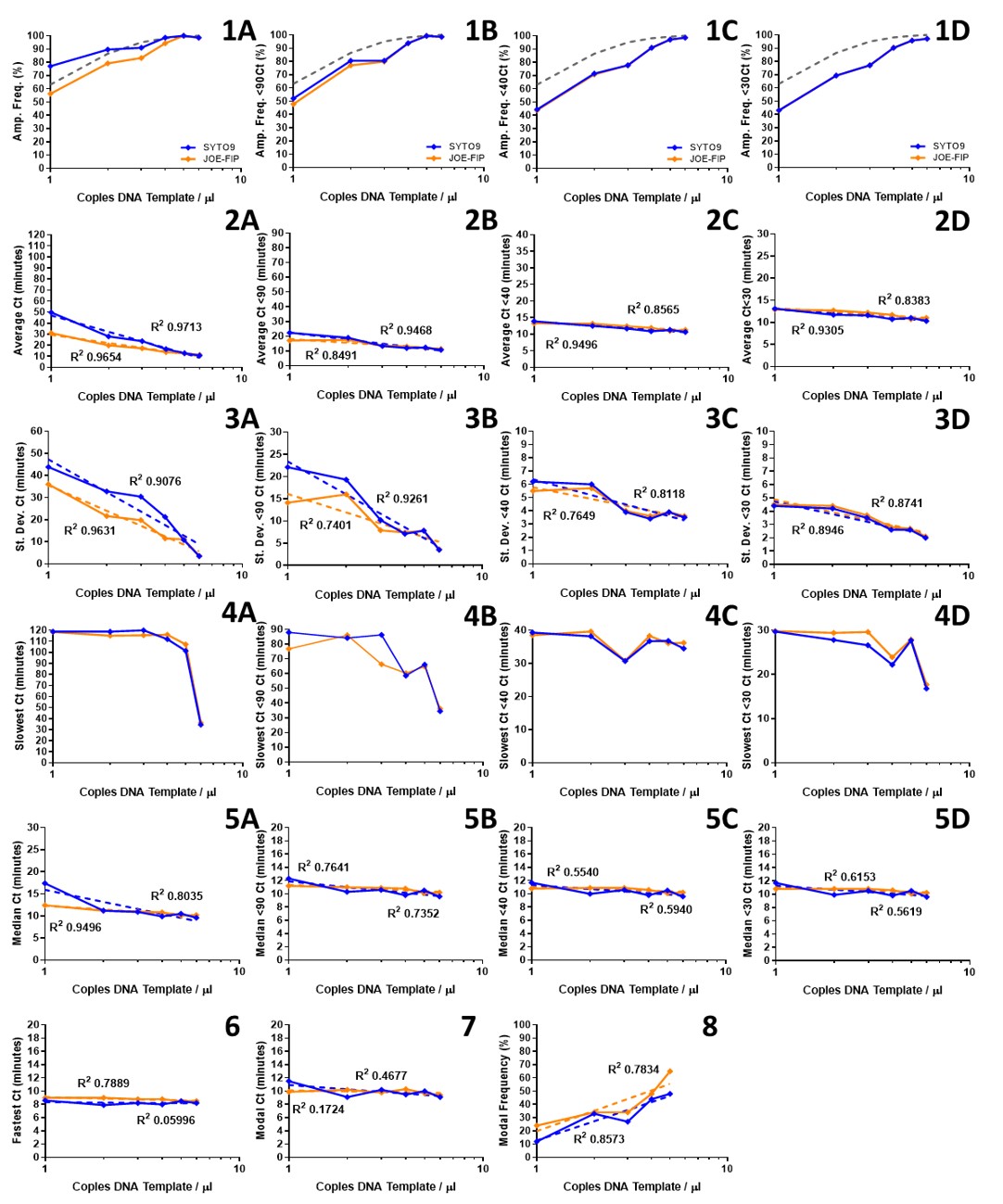


**Figure S9: Summary of normalised data for 35Sp BIP DB native noF3B3.** 35Sp primers, artificial template, 72 replicates per assay and dual detection with JOE labelled FIP and SYTO9. JOE FIP results in orange, SYTO9 results in blue. **(A,B,C,D)** Full assay time and truncated to 90, 40 and 30 minutes. **(1)** Amplification frequency, **(2)** average Ct, **(3)** standard deviation, **(4)** slowest Ct, **(5)** median Ct, **(6)** fastest Ct, **(7)** mode Ct and **(8)** the percentage modal frequency (the percentage of replicates comprising the mode Ct against the total number of replicates).


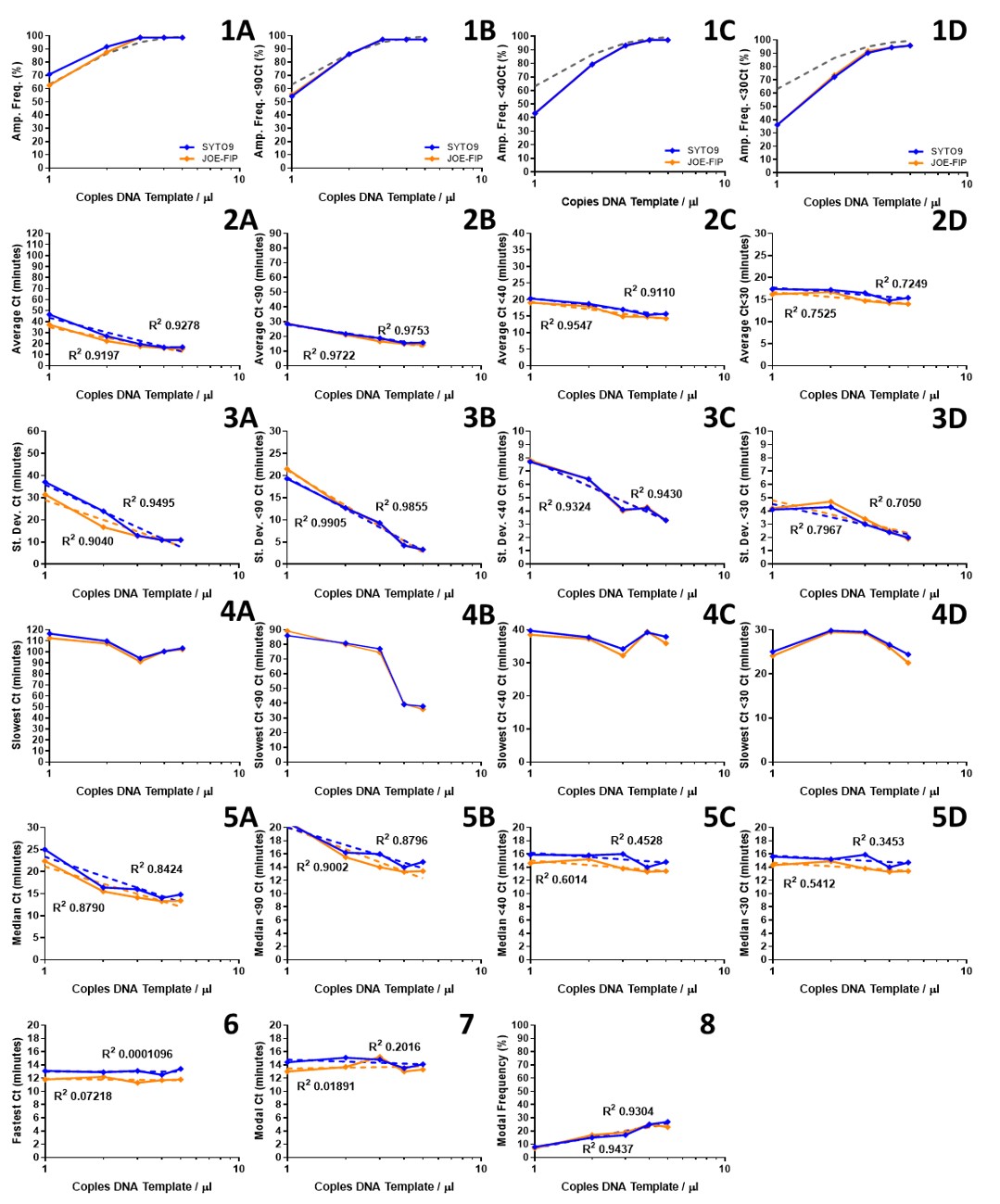


**Figure S10: Summary of normalised data for 35Sp pART7 native F3B3.** 35Sp primers, pART7 plasmid template, 72 replicates per assay and dual detection with JOE labelled FIP and SYTO9. JOE FIP results in orange, SYTO9 results in blue. **(A,B,C,D)** Full assay time and truncated to 90, 40 and 30 minutes. **(1)** Amplification frequency, **(2)** average Ct, **(3)** standard deviation, **(4)** slowest Ct, **(5)** median Ct, **(6)** fastest Ct, **(7)** mode Ct and **(8)** the percentage modal frequency (the percentage of replicates comprising the mode Ct against the total number of replicates).


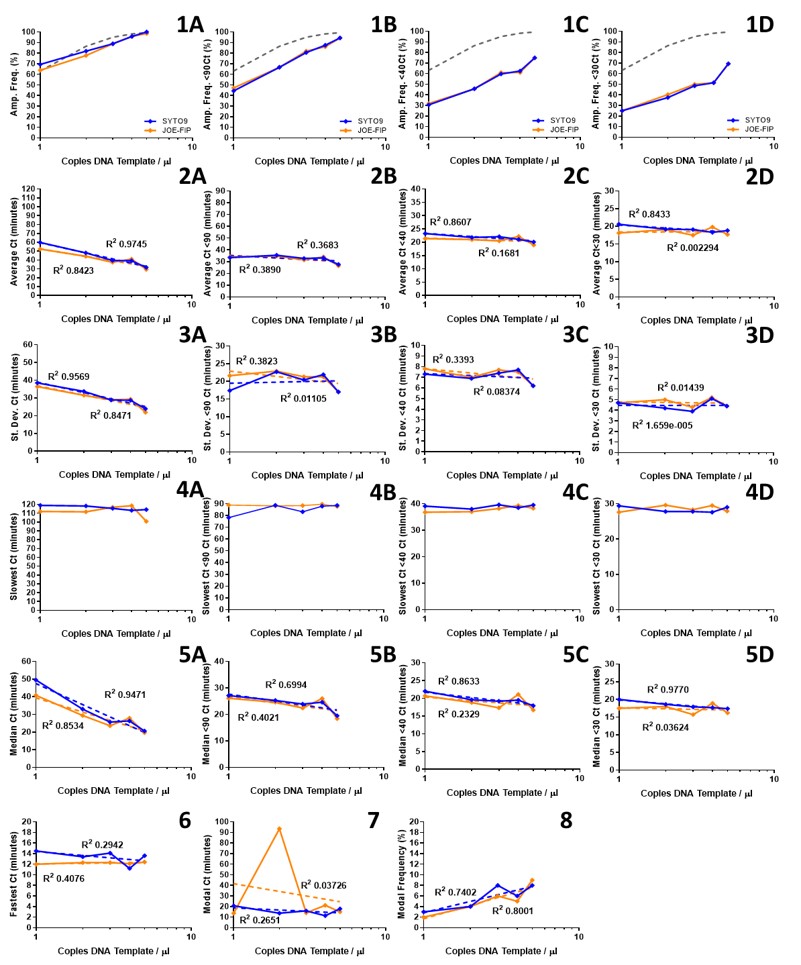


**Figure S11: Summary of normalised data for 35Sp pART7 native without F3B3.** 35Sp primers, pART7 plasmid template, 72 replicates per assay and dual detection with JOE labelled FIP and SYTO9. JOE FIP results in orange, SYTO9 results in blue. **(A,B,C,D)** Full assay time and truncated to 90, 40 and 30 minutes. **(1)** Amplification frequency, **(2)** average Ct, **(3)** standard deviation, **(4)** slowest Ct, **(5)** median Ct, **(6)** fastest Ct, **(7)** mode Ct and **(8)** the percentage modal frequency (the percentage of replicates comprising the mode Ct against the total number of replicates).


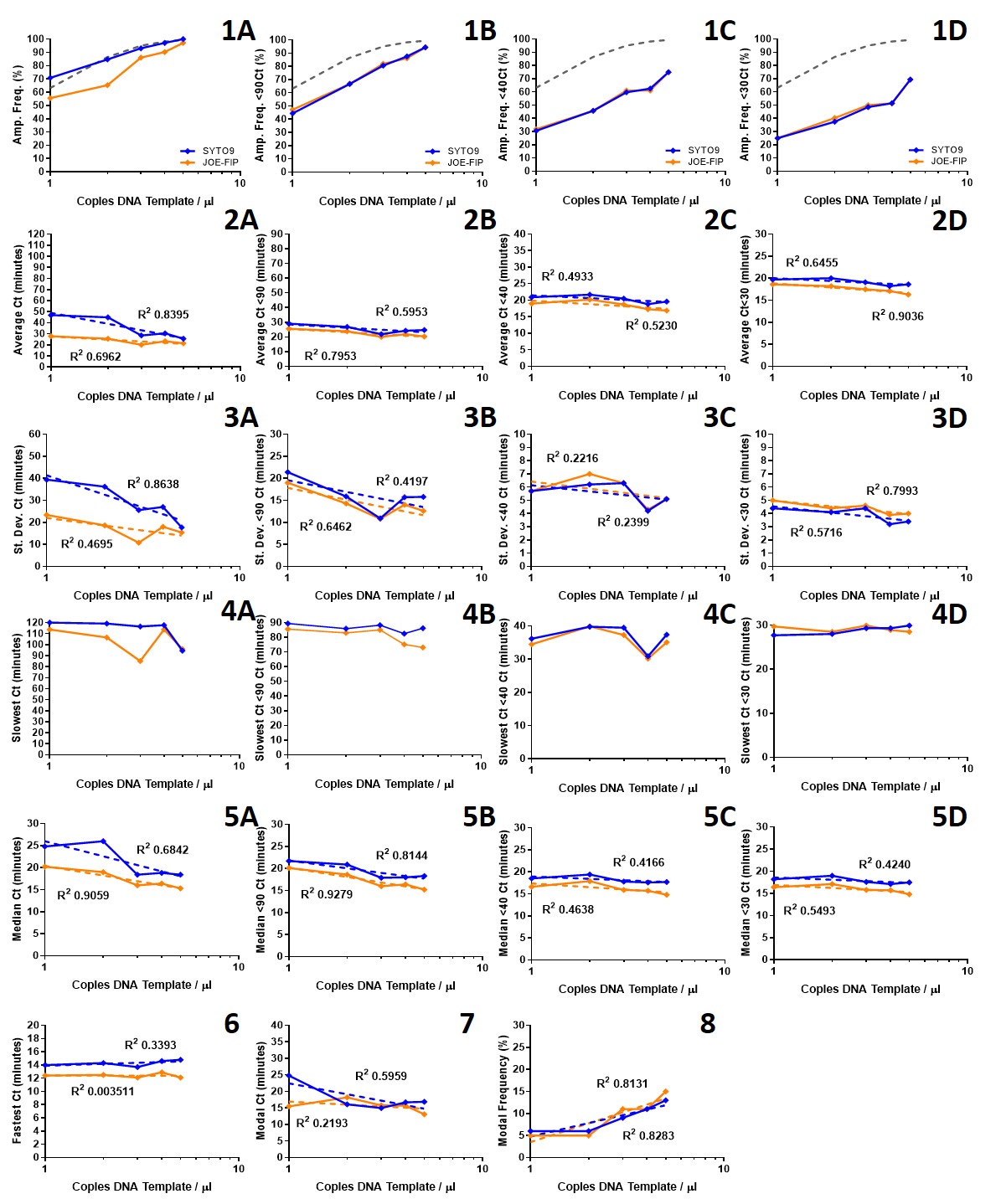


**Figure S12 Summary of normalised data for 35Sp 5%Bt11 native F3B3.** 35Sp primers, 5% Bt11 native genomic DNA template, 72 replicates per assay and dual detection with JOE labelled FIP and SYTO9. JOE FIP results in orange, SYTO9 results in blue. **(A,B,C,D)** Full assay time and truncated to 90, 40 and 30 minutes. **(1)** Amplification frequency, **(2)** average Ct, **(3)** standard deviation, **(4)** slowest Ct, **(5)** median Ct, **(6)** fastest Ct, **(7)** mode Ct and **(8)** the percentage modal frequency (the percentage of replicates comprising the mode Ct against the total number of replicates).


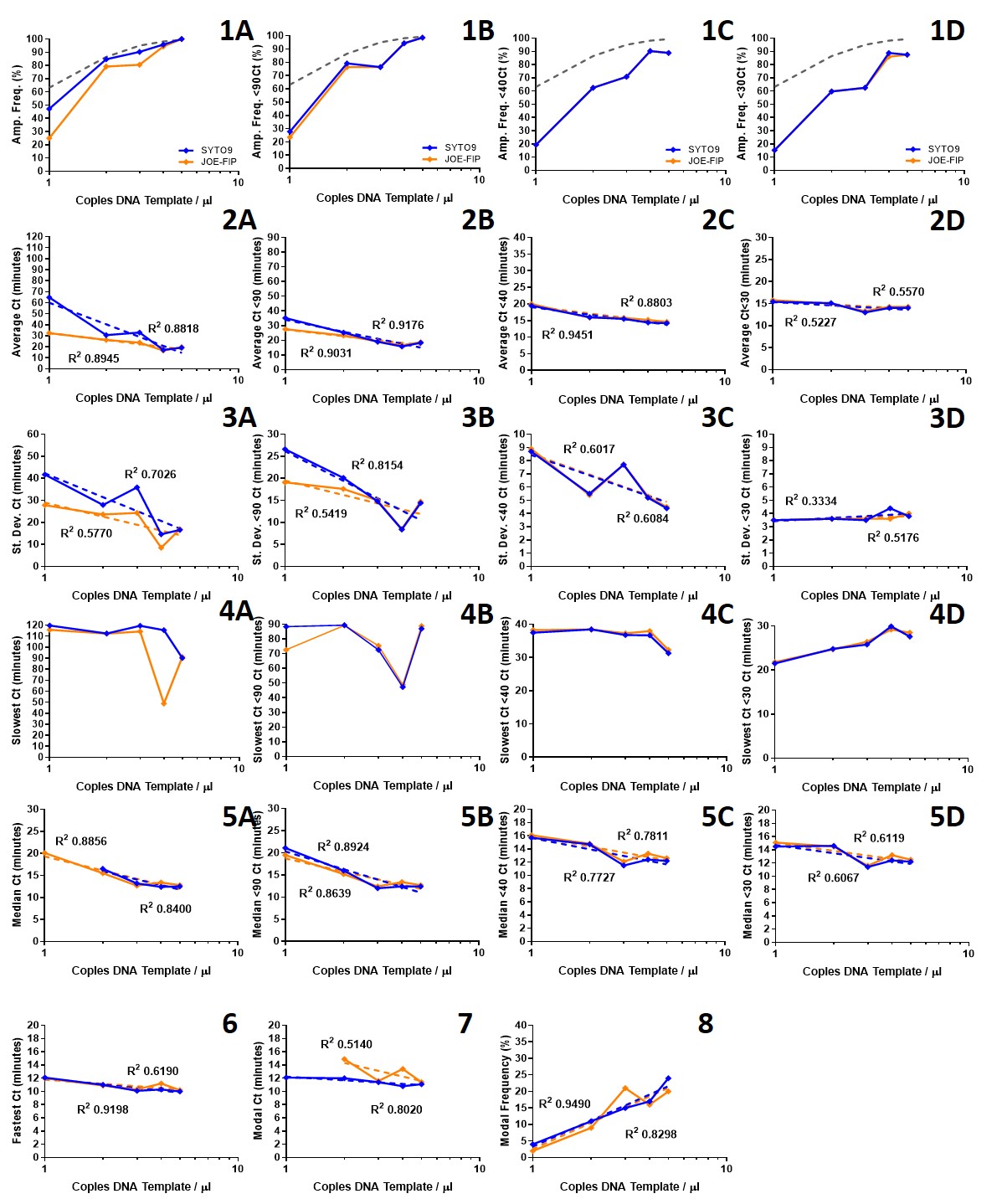


**Figure S13: Summary of normalised data for 35Sp 5%Bt11 denatured F3B3.** 35Sp primers, 5% Bt11 denatured genomic DNA template, 72 replicates per assay and dual detection with JOE labelled FIP and SYTO9. JOE FIP results in orange, SYTO9 results in blue. **(A,B,C,D)** Full assay time and truncated to 90, 40 and 30 minutes. **(1)** Amplification frequency, **(2)** average Ct, **(3)** standard deviation, **(4)** slowest Ct, **(5)** median Ct, **(6)** fastest Ct, **(7)** mode Ct and **(8)** the percentage modal frequency (the percentage of replicates comprising the mode Ct against the total number of replicates).


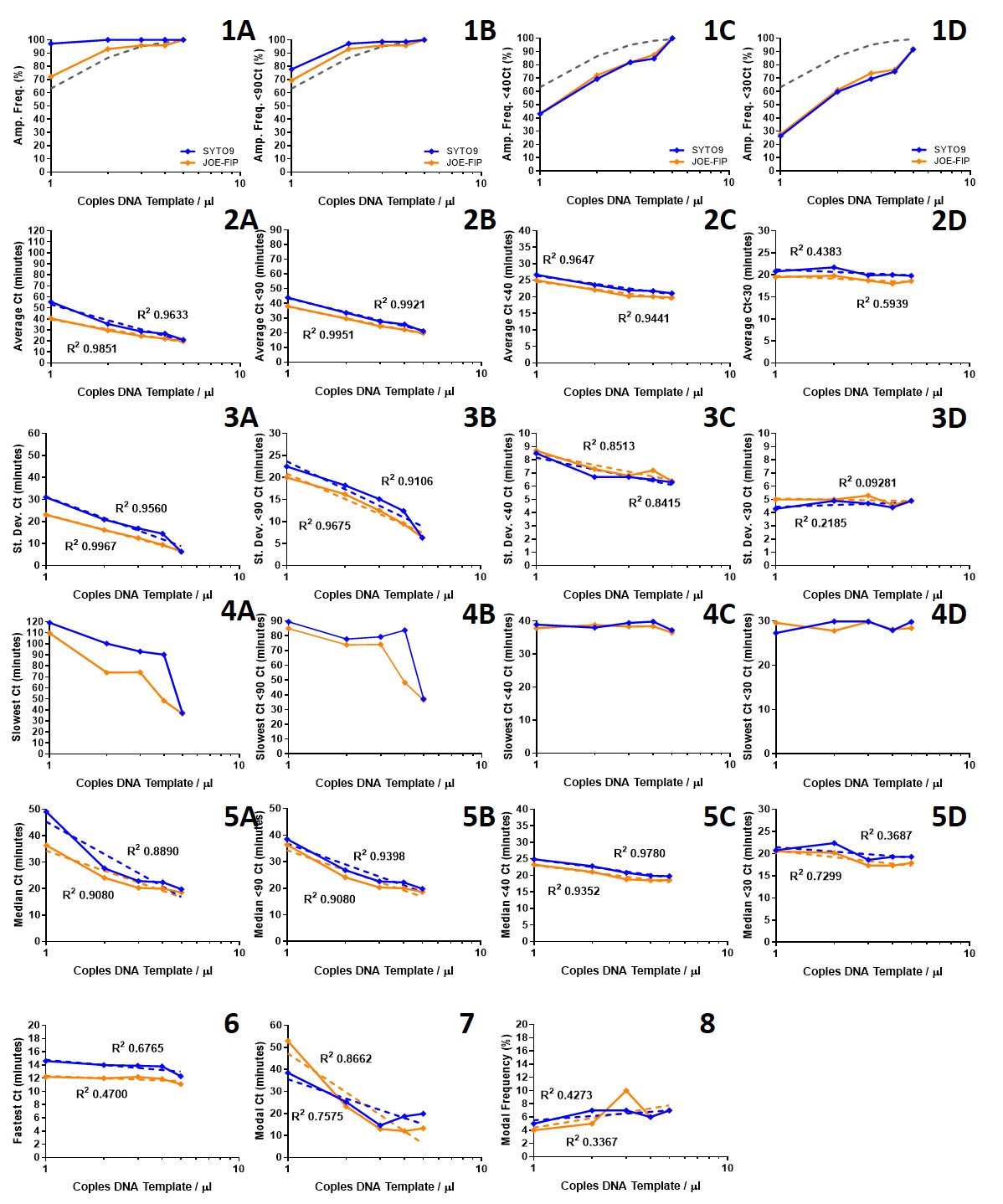


**Figure S14: Summary of normalised data for 35Sp 5%Bt11 native without F3B3.** 35Sp primers, 5% Bt11 native genomic DNA template, 72 replicates per assay and dual detection with JOE labelled FIP and SYTO9. JOE FIP results in orange, SYTO9 results in blue. **(A,B,C,D)** Full assay time and truncated to 90, 40 and 30 minutes. **(1)** Amplification frequency, **(2)** average Ct, **(3)** standard deviation, **(4)** slowest Ct, **(5)** median Ct, **(6)** fastest Ct, **(7)** mode Ct and **(8)** the percentage modal frequency (the percentage of replicates comprising the mode Ct against the total number of replicates).


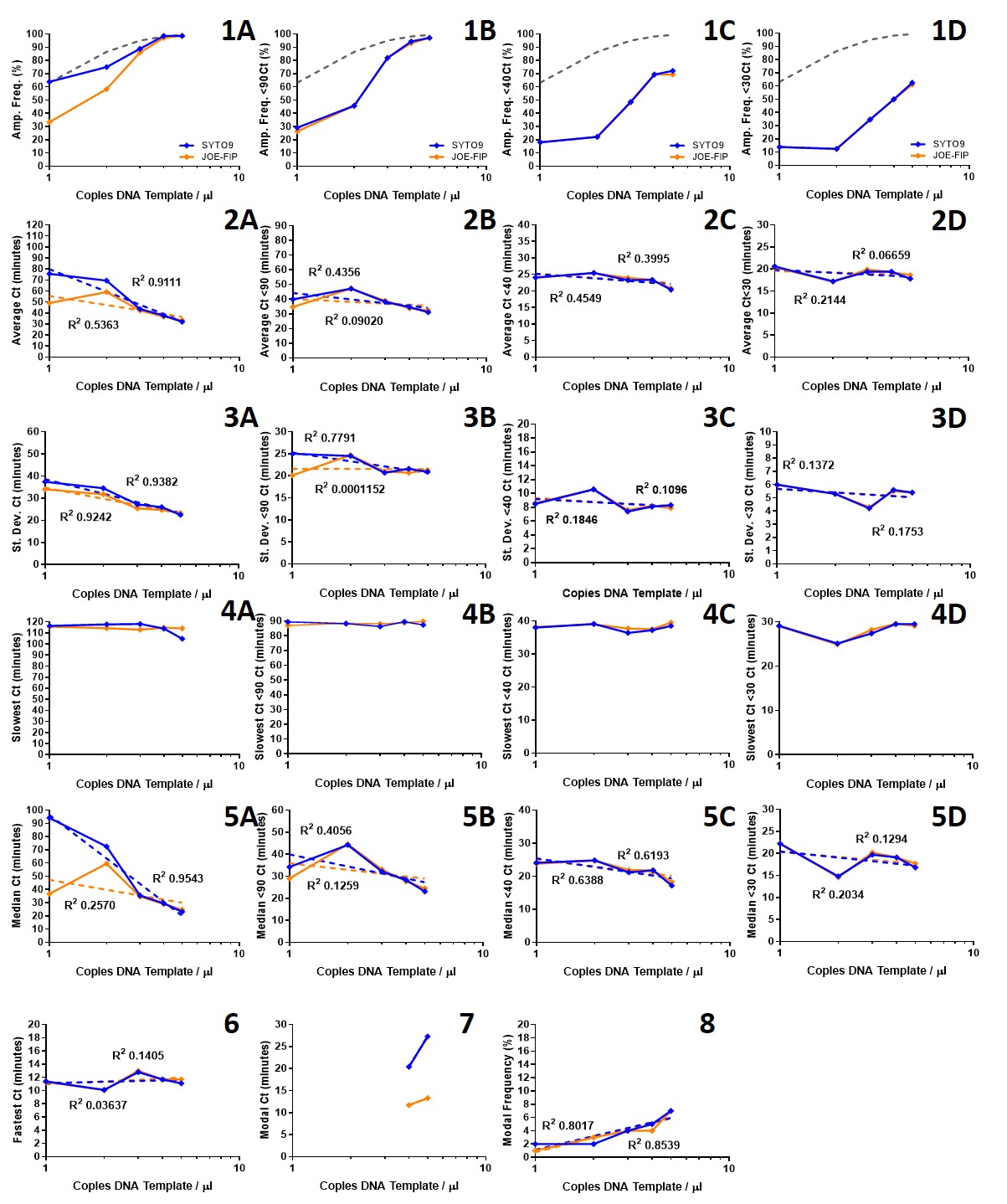


**Figure S15: Summary of normalised data for 35Sp 5%Bt11 denatured without F3B3.** 35Sp primers, 5% Bt11 denatured genomic DNA template, 72 replicates per assay and dual detection with JOE labelled FIP and SYTO9. JOE FIP results in orange, SYTO9 results in blue. **(A,B,C,D)** Full assay time and truncated to 90, 40 and 30 minutes. **(1)** Amplification frequency, **(2)** average Ct, **(3)** standard deviation, **(4)** slowest Ct, **(5)** median Ct, **(6)** fastest Ct, **(7)** mode Ct and **(8)** the percentage modal frequency (the percentage of replicates comprising the mode Ct against the total number of replicates).


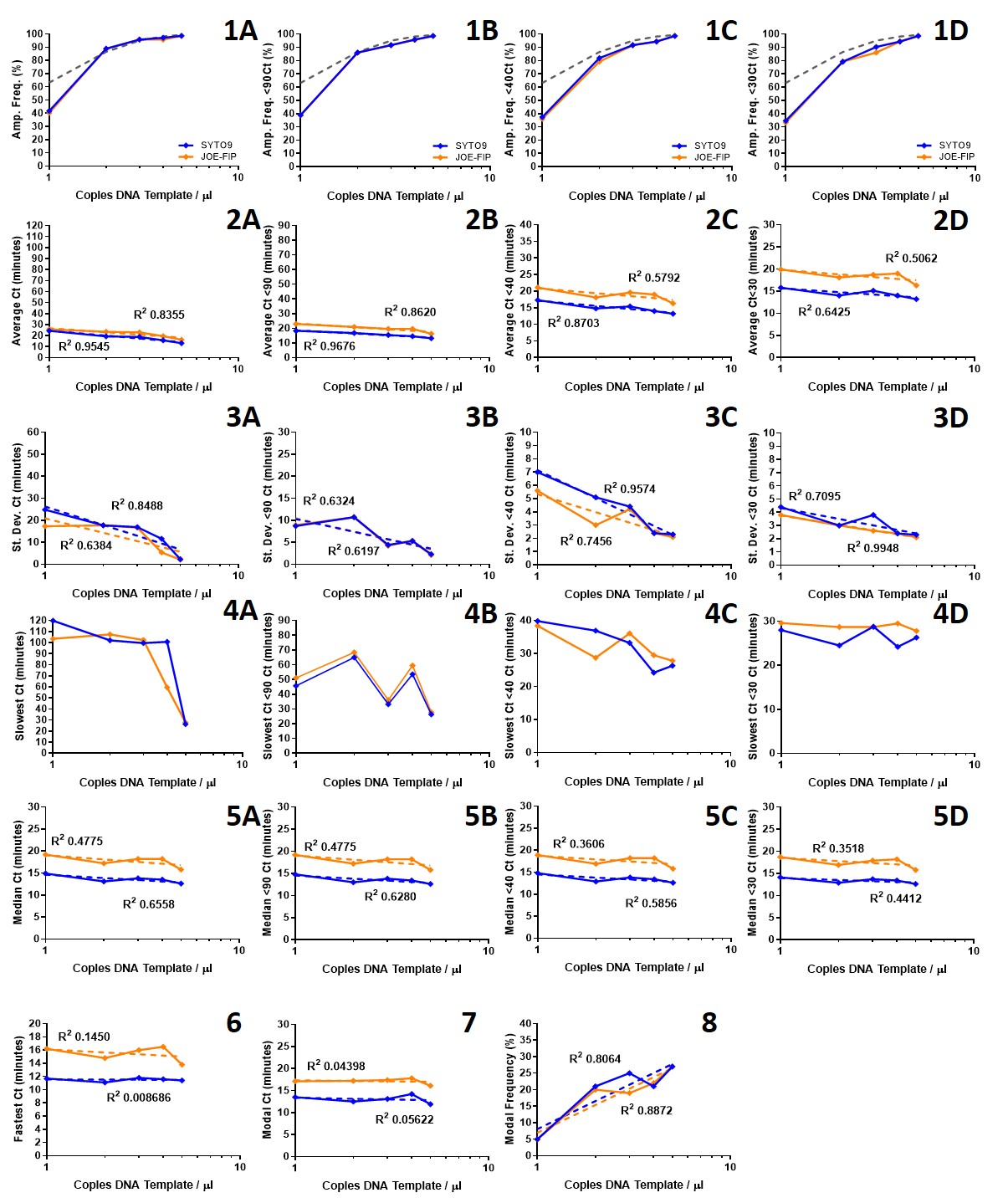


**Figure S16: Summary of normalised data for NOSt 5%Bt11 native F3B3.** NOSt primers, 5% Bt11 native genomic DNA template, 72 replicates per assay and dual detection with JOE labelled FIP and SYTO9. JOE FIP results in orange, SYTO9 results in blue. **(A,B,C,D)** Full assay time and truncated to 90, 40 and 30 minutes. **(1)** Amplification frequency, **(2)** average Ct, **(3)** standard deviation, **(4)** slowest Ct, **(5)** median Ct, **(6)** fastest Ct, **(7)** mode Ct and **(8)** the percentage modal frequency (the percentage of replicates comprising the mode Ct against the total number of replicates).


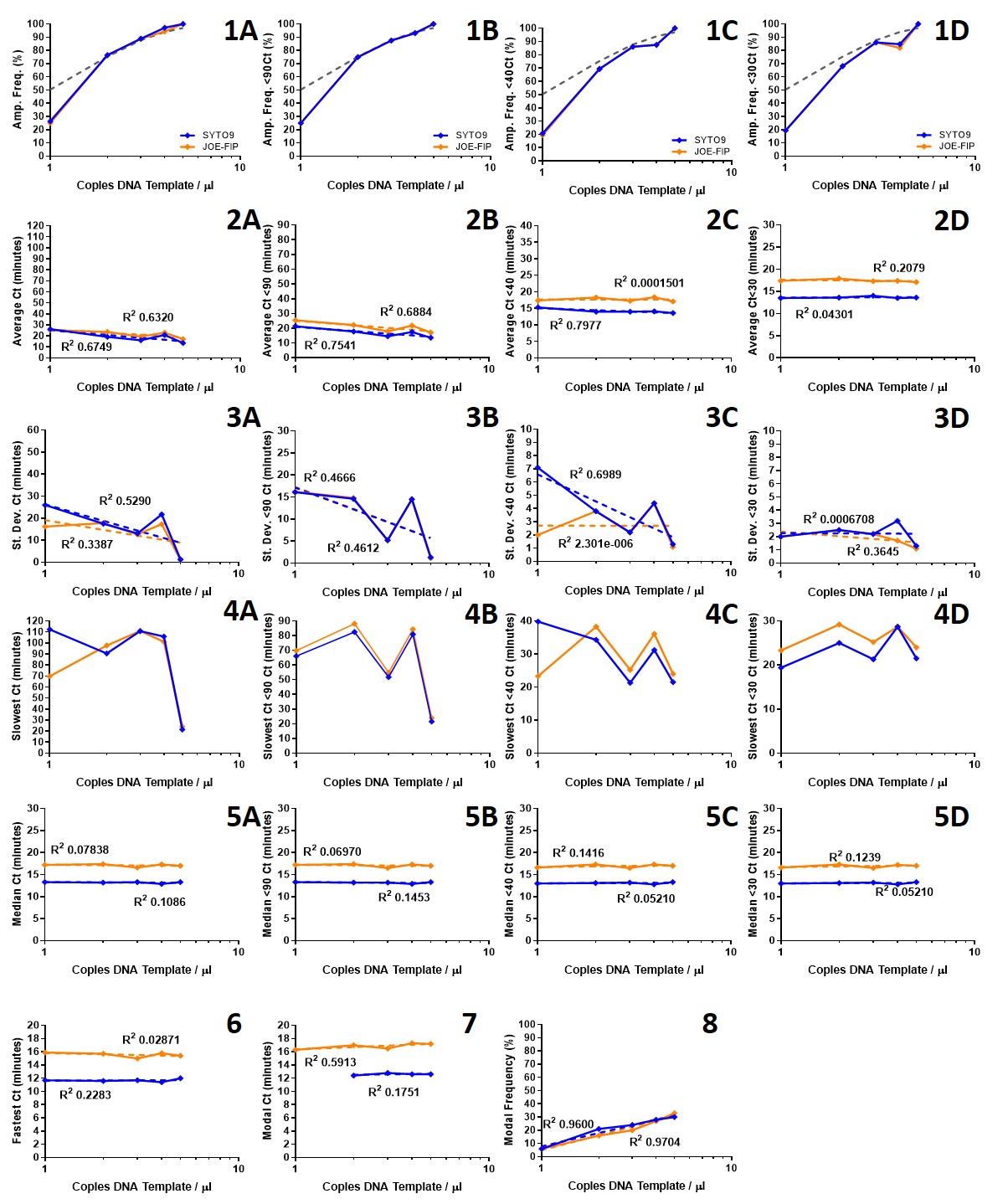


**Figure S17: Summary of normalised data for NOSt 5%Bt11 denatured F3B3.** NOSt primers, 5% Bt11 denatured genomic DNA template, 72 replicates per assay and dual detection with JOE labelled FIP and SYTO9. JOE FIP results in orange, SYTO9 results in blue. **(A,B,C,D)** Full assay time and truncated to 90, 40 and 30 minutes. **(1)** Amplification frequency, **(2)** average Ct, **(3)** standard deviation, **(4)** slowest Ct, **(5)** median Ct, **(6)** fastest Ct, **(7)** mode Ct and **(8)** the percentage modal frequency (the percentage of replicates comprising the mode Ct against the total number of replicates).


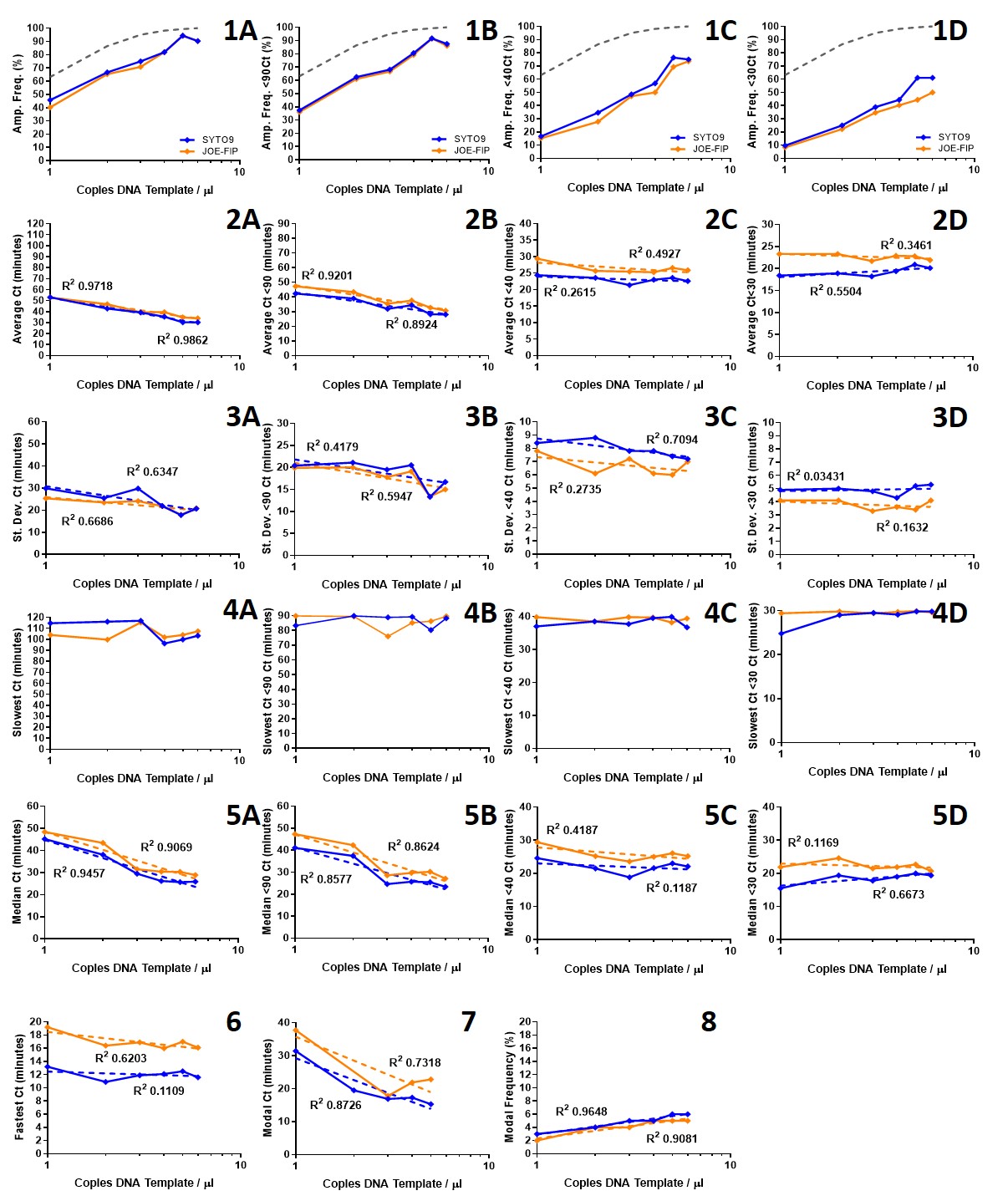


**Figure S18: Summary of normalised data for NOSt 5%Bt11 native without F3B3 (version 1).** NOSt primers, 5% Bt11 native genomic DNA template, 72 replicates per assay and dual detection with JOE labelled FIP and SYTO9. JOE FIP results in orange, SYTO9 results in blue. **(A,B,C,D)** Full assay time and truncated to 90, 40 and 30 minutes. **(1)** Amplification frequency, **(2)** average Ct, **(3)** standard deviation, **(4)** slowest Ct, **(5)** median Ct, **(6)** fastest Ct, **(7)** mode Ct and **(8)** the percentage modal frequency (the percentage of replicates comprising the mode Ct against the total number of replicates).


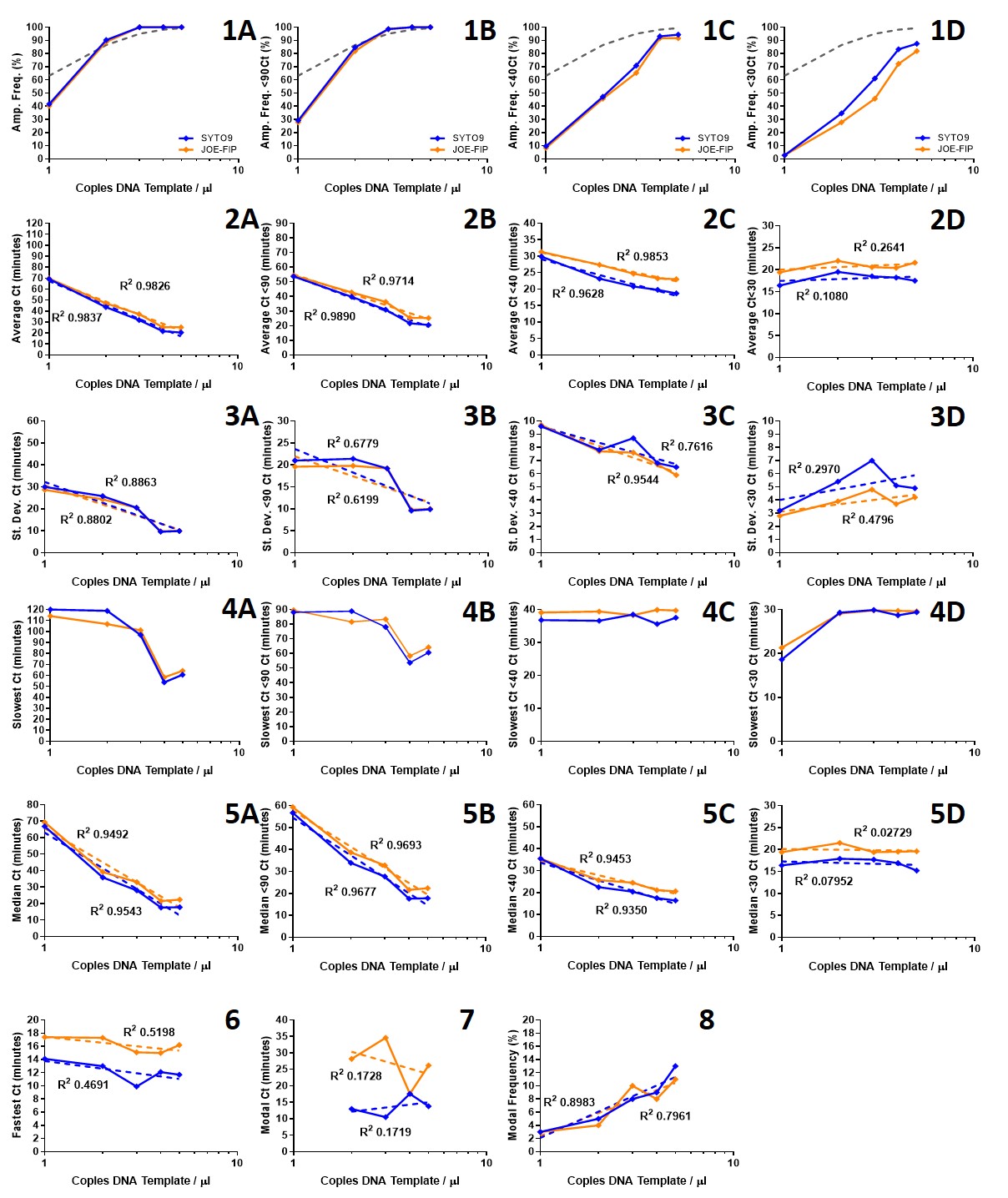


**Figure S19: Summary of normalised data for NOSt 5%Bt11 denatured without F3B3 (version 1).** NOSt primers, 5% Bt11 denatured genomic DNA template, 72 replicates per assay and dual detection with JOE labelled FIP and SYTO9. JOE FIP results in orange, SYTO9 results in blue. **(A,B,C,D)** Full assay time and truncated to 90, 40 and 30 minutes. **(1)** Amplification frequency, **(2)** average Ct, **(3)** standard deviation, **(4)** slowest Ct, **(5)** median Ct, **(6)** fastest Ct, **(7)** mode Ct and **(8)** the percentage modal frequency (the percentage of replicates comprising the mode Ct against the total number of replicates).


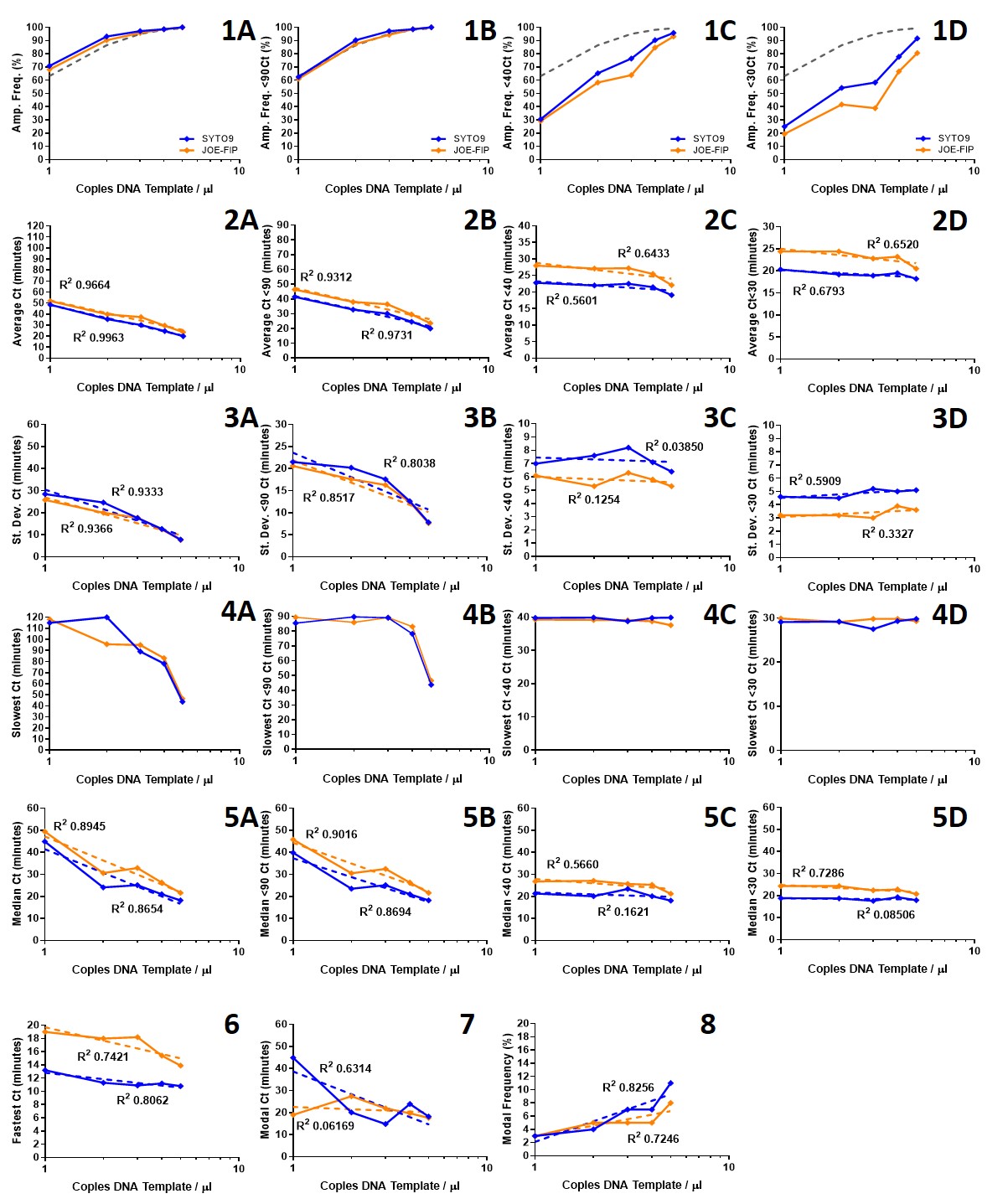


**Figure S20: Summary of normalised data for NOSt 5%Bt11 native without F3B3 (version 2).** NOSt primers, 5% Bt11 native genomic DNA template, 72 replicates per assay and dual detection with JOE labelled FIP and SYTO9. JOE FIP results in orange, SYTO9 results in blue. **(A,B,C,D)** Full assay time and truncated to 90, 40 and 30 minutes. **(1)** Amplification frequency, **(2)** average Ct, **(3)** standard deviation, **(4)** slowest Ct, **(5)** median Ct, **(6)** fastest Ct, **(7)** mode Ct and **(8)** the percentage modal frequency (the percentage of replicates comprising the mode Ct against the total number of replicates).

**
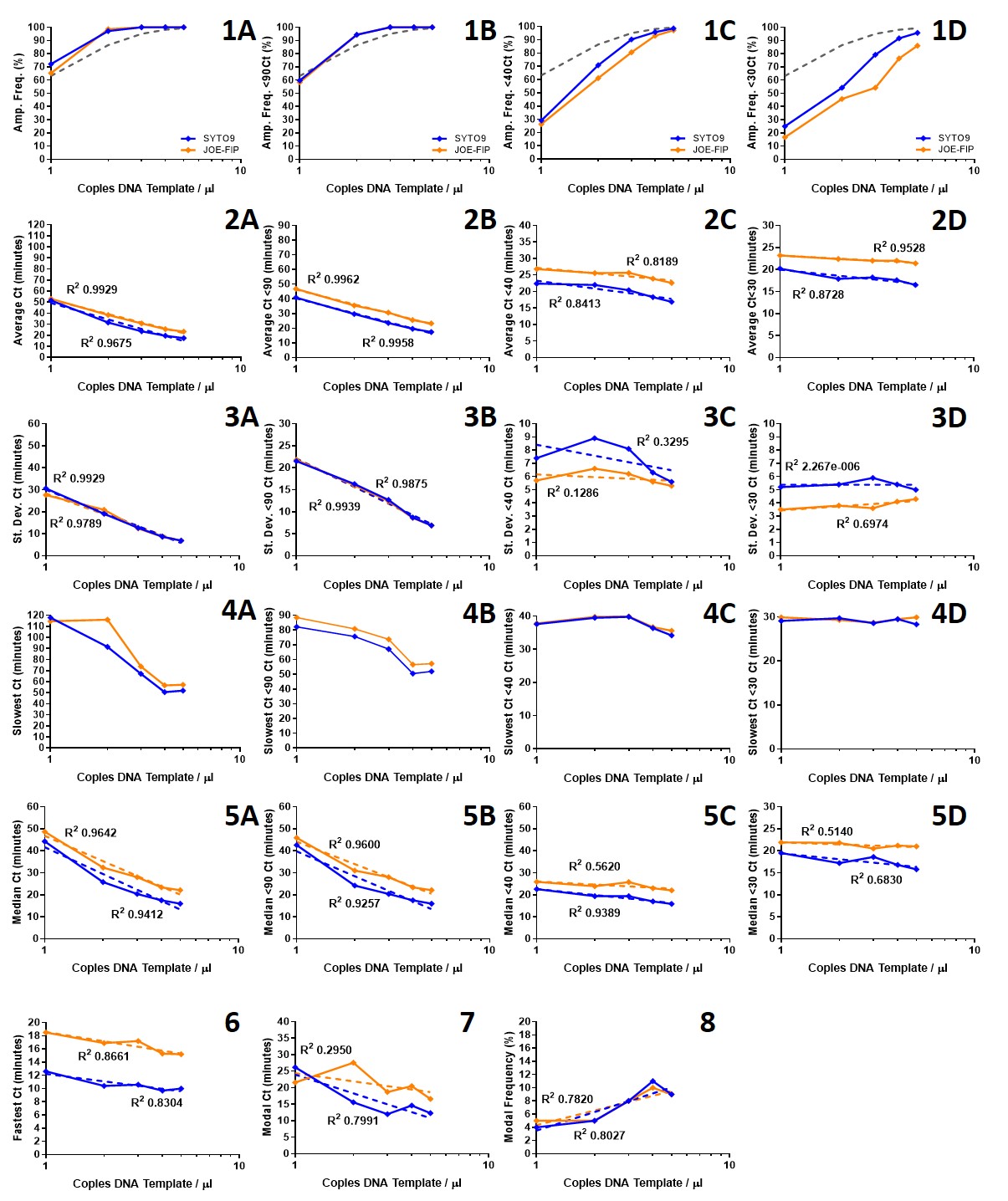
**

**Figure S21: Summary of normalised data for NOSt 5%Bt11 denatured without F3B3 (version 2).** NOSt primers, 5% Bt11 denatured genomic DNA template, 72 replicates per assay and dual detection with JOE labelled FIP and SYTO9. JOE FIP results in orange, SYTO9 results in blue. **(A,B,C,D)** Full assay time and truncated to 90, 40 and 30 minutes. **(1)** Amplification frequency, **(2)** average Ct, **(3)** standard deviation, **(4)** slowest Ct, **(5)** median Ct, **(6)** fastest Ct, **(7)** mode Ct and **(8)** the percentage modal frequency (the percentage of replicates comprising the mode Ct against the total number of replicates).


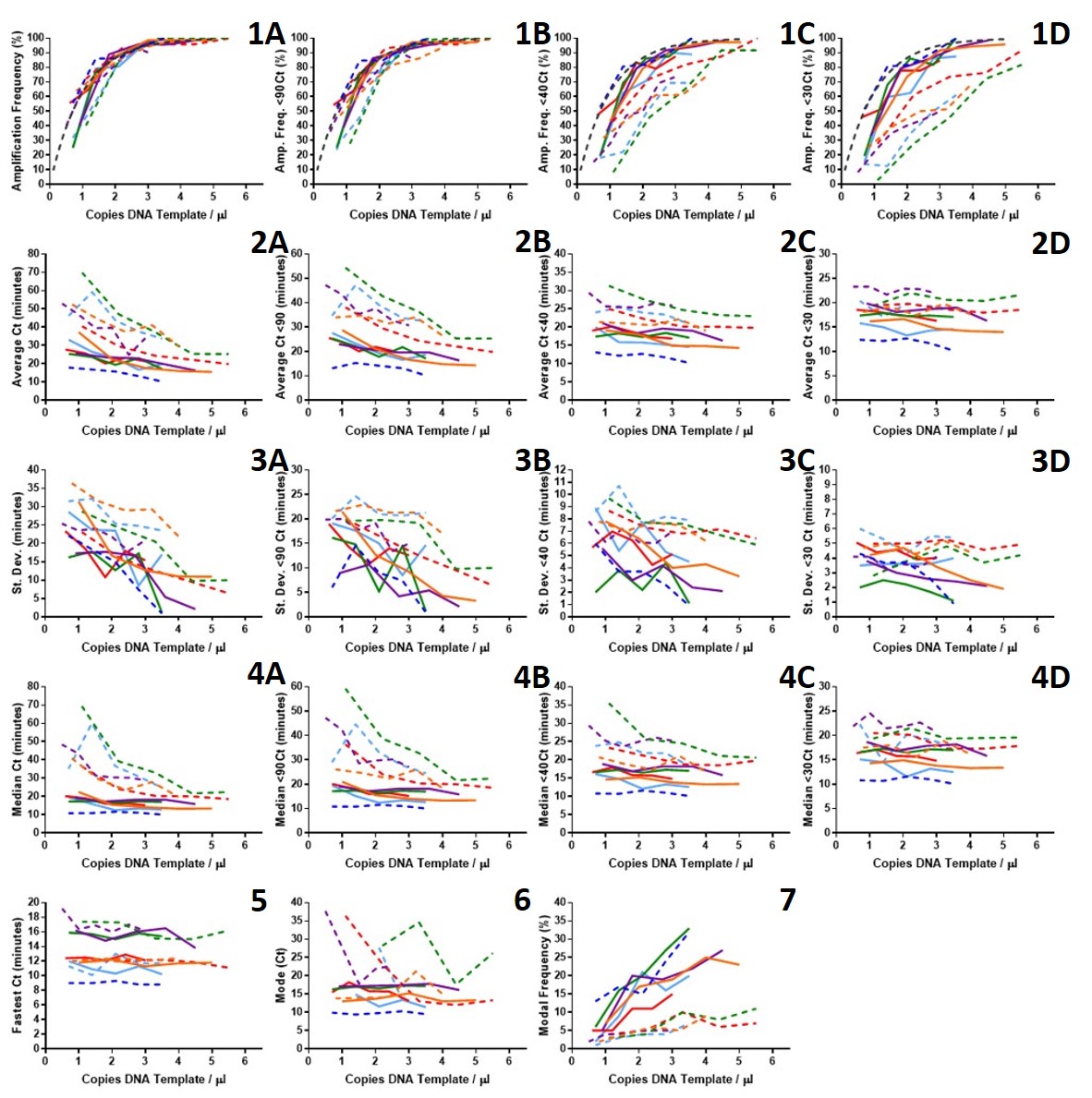


**Figure S22. Combined data for dual detection quenched JOE FIP detection of various templates at low copy numbers normalised to amplification frequency with and without displacement primers.** Predicted results for amplification frequency at low copy number in black, 35Sp artificial template without F3/B3 in dark blue (dashed), 35Sp pART7 linearised plasmid DNA in orange (dashed for without F3/B3), 35Sp native genomic DNA in red (dashed for without F3/B3), 35Sp denatured genomic DNA in light blue (dashed for without F3/B3), NOSt native genomic DNA in purple (dashed for without F3/B3) and NOSt denatured genomic DNA in green (dashed for without F3/B3). Genomic DNA extracted from 5%Bt11 CRM maize. **(A,B,C,D)** Full assay time and truncated to 90, 40 and 30 minutes. **(1)** Amplification frequency, **(2)** average Ct, **(3)** standard deviation, **(4)** median Ct, **(5)** fastest Ct, **(6)** mode Ct and **(7)** the percentage modal frequency (the percentage of replicates comprising the mode Ct against the total number of replicates).


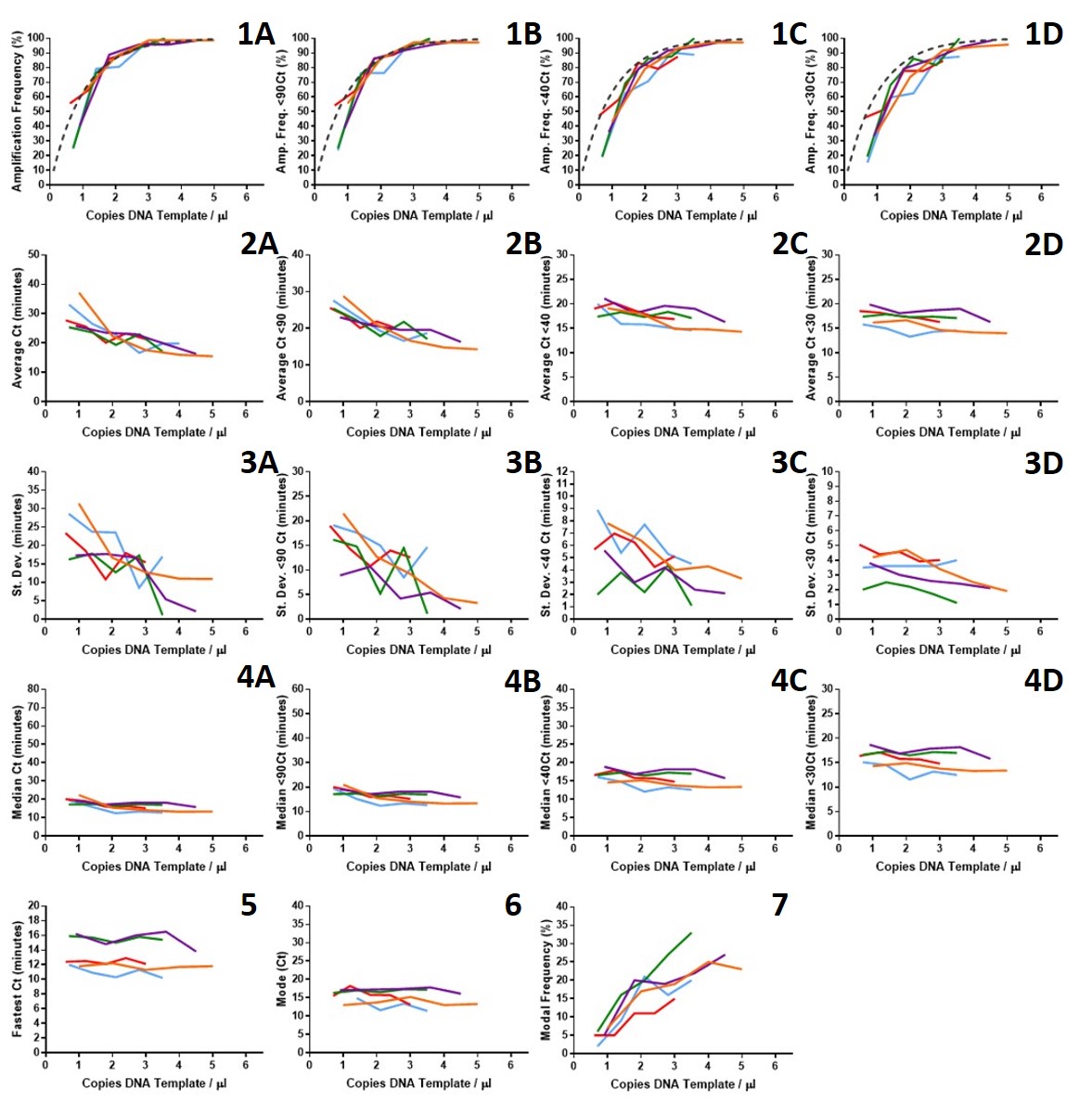


**Figure S23. Combined data for dual detection quenched JOE FIP detection of various templates at low copy numbers normalised to amplification frequency with displacement primers.** Predicted results for amplification frequency at low copy number in black, 35Sp pART7 linearised plasmid DNA in orange, 35Sp native genomic DNA in red, 35Sp denatured genomic DNA in light blue, NOSt native genomic DNA in purple and NOSt denatured genomic DNA in green. Genomic DNA extracted from 5%Bt11 CRM maize. **(A,B,C,D)** Full assay time and truncated to 90, 40 and 30 minutes. **(1)** Amplification frequency, **(2)** average Ct, **(3)** standard deviation, **(4)** median Ct, **(5)** fastest Ct, **(6)** mode Ct and **(7)** the percentage modal frequency (the percentage of replicates comprising the mode Ct against the total number of replicates).


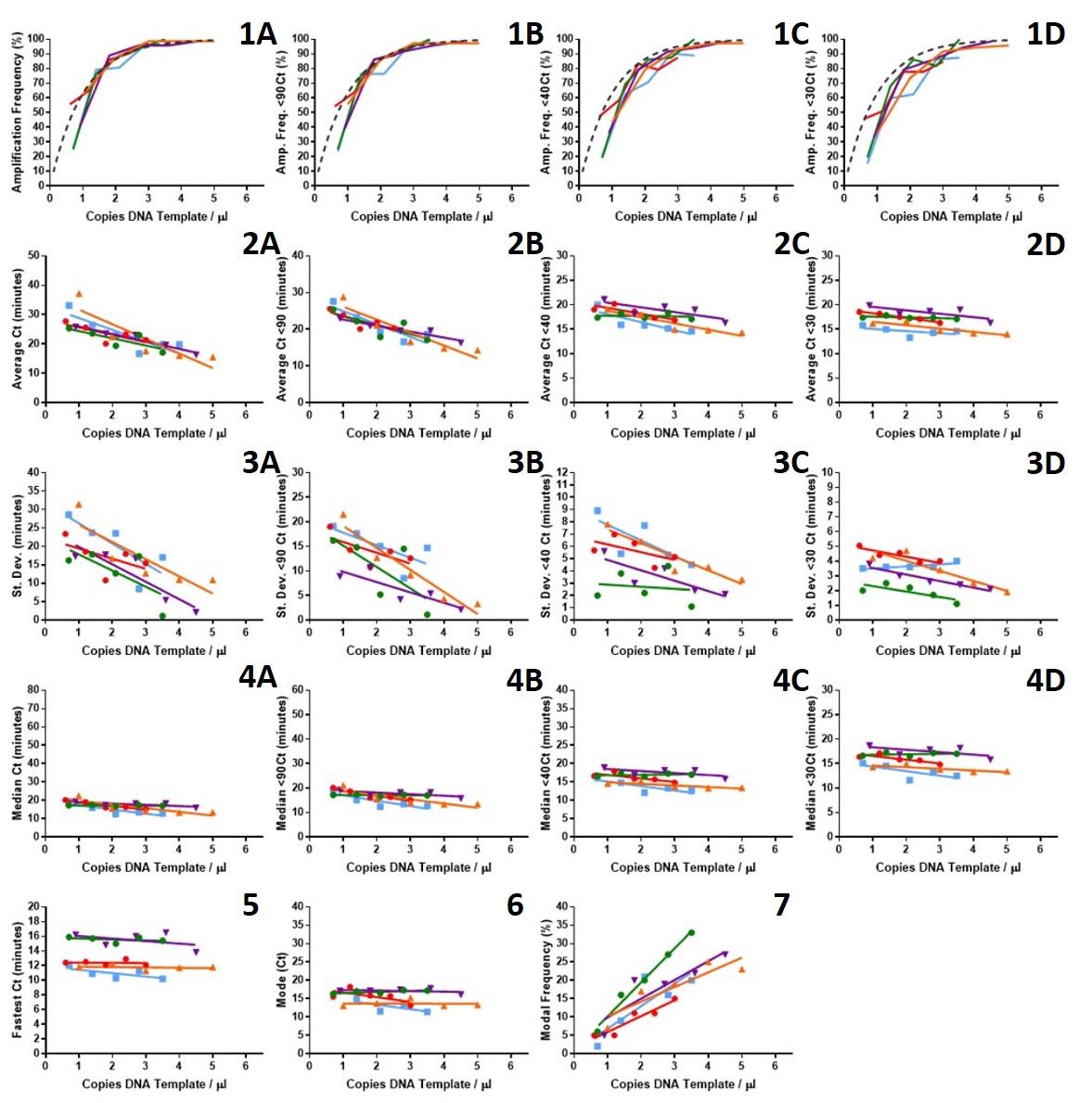


**Figure S24. Combined data for dual detection quenched JOE FIP detection of various templates at low copy numbers normalised to amplification frequency with displacement primers (trendlines).** Predicted results for amplification frequency at low copy number in black, 35Sp pART7 linearised plasmid DNA in orange, 35Sp native genomic DNA in red, 35Sp denatured genomic DNA in light blue, NOSt native genomic DNA in purple and NOSt denatured genomic DNA in green. Genomic DNA extracted from 5%Bt11 CRM maize. **(A,B,C,D)** Full assay time and truncated to 90, 40 and 30 minutes. **(1)** Amplification frequency, **(2)** average Ct, **(3)** standard deviation, **(4)** median Ct, **(5)** fastest Ct, **(6)** mode Ct and **(7)** the percentage modal frequency (the percentage of replicates comprising the mode Ct against the total number of replicates).


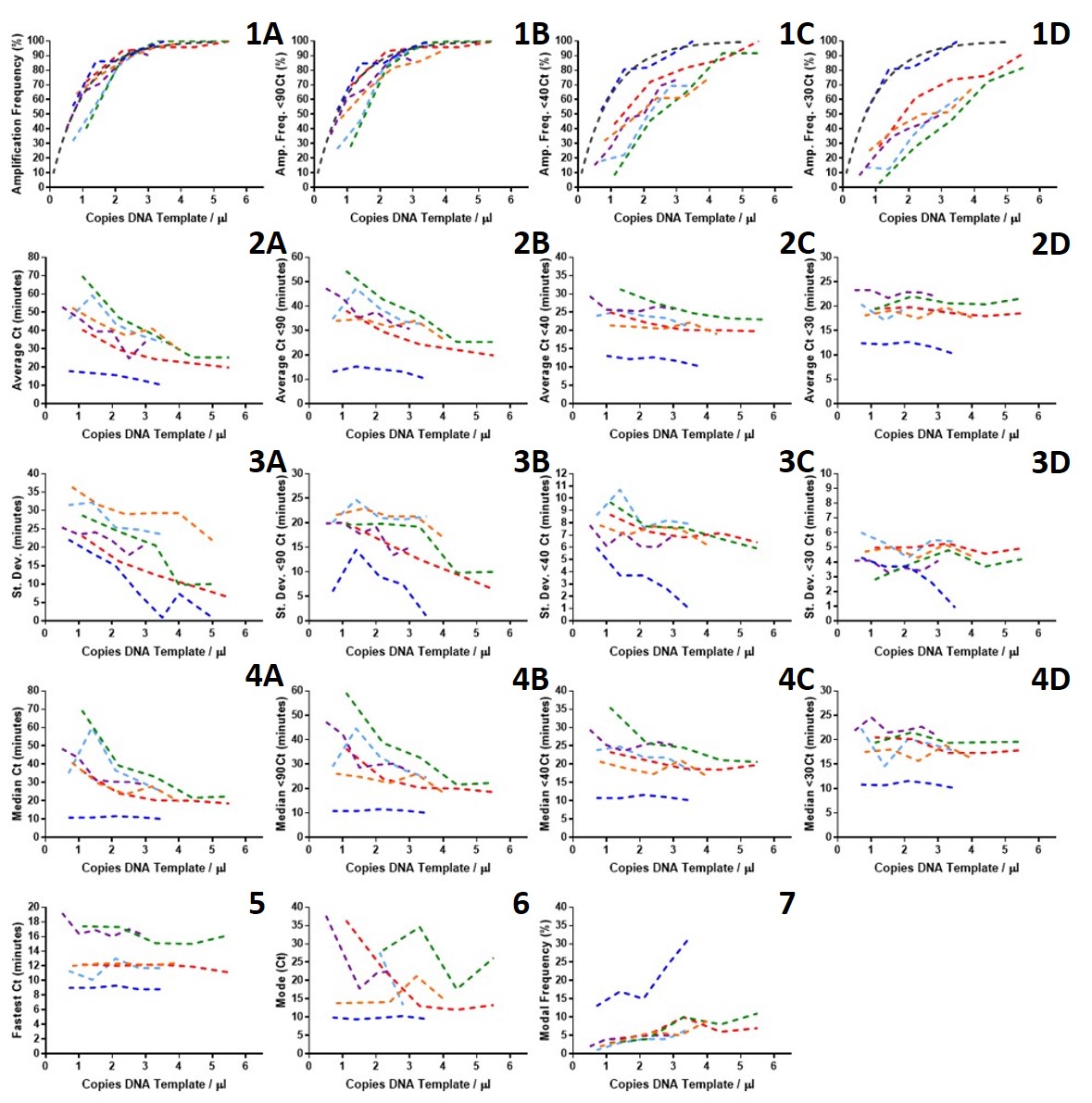


**Figure S25. Combined data for dual detection quenched JOE FIP detection of various templates at low copy numbers normalised to amplification frequency without displacement primers.** Predicted results for amplification frequency at low copy number in black, 35Sp artificial template without F3/B3 in dark blue (dashed), 35Sp pART7 linearised plasmid DNA without F3/B3 in orange (dashed), 35Sp native genomic DNA without F3/B3 in red (dashed), 35Sp denatured genomic DNA without F3/B3 in light blue (dashed), NOSt native genomic DNA without F3/B3 in purple (dashed) and NOSt denatured genomic DNA without F3/B3 in green (dashed). Genomic DNA extracted from 5%Bt11 CRM maize. **(A,B,C,D)** Full assay time and truncated to 90, 40 and 30 minutes. **(1)** Amplification frequency, **(2)** average Ct, **(3)** standard deviation, **(4)** median Ct, **(5)** fastest Ct, **(6)** mode Ct and **(7)** the percentage modal frequency (the percentage of replicates comprising the mode Ct against the total number of replicates).


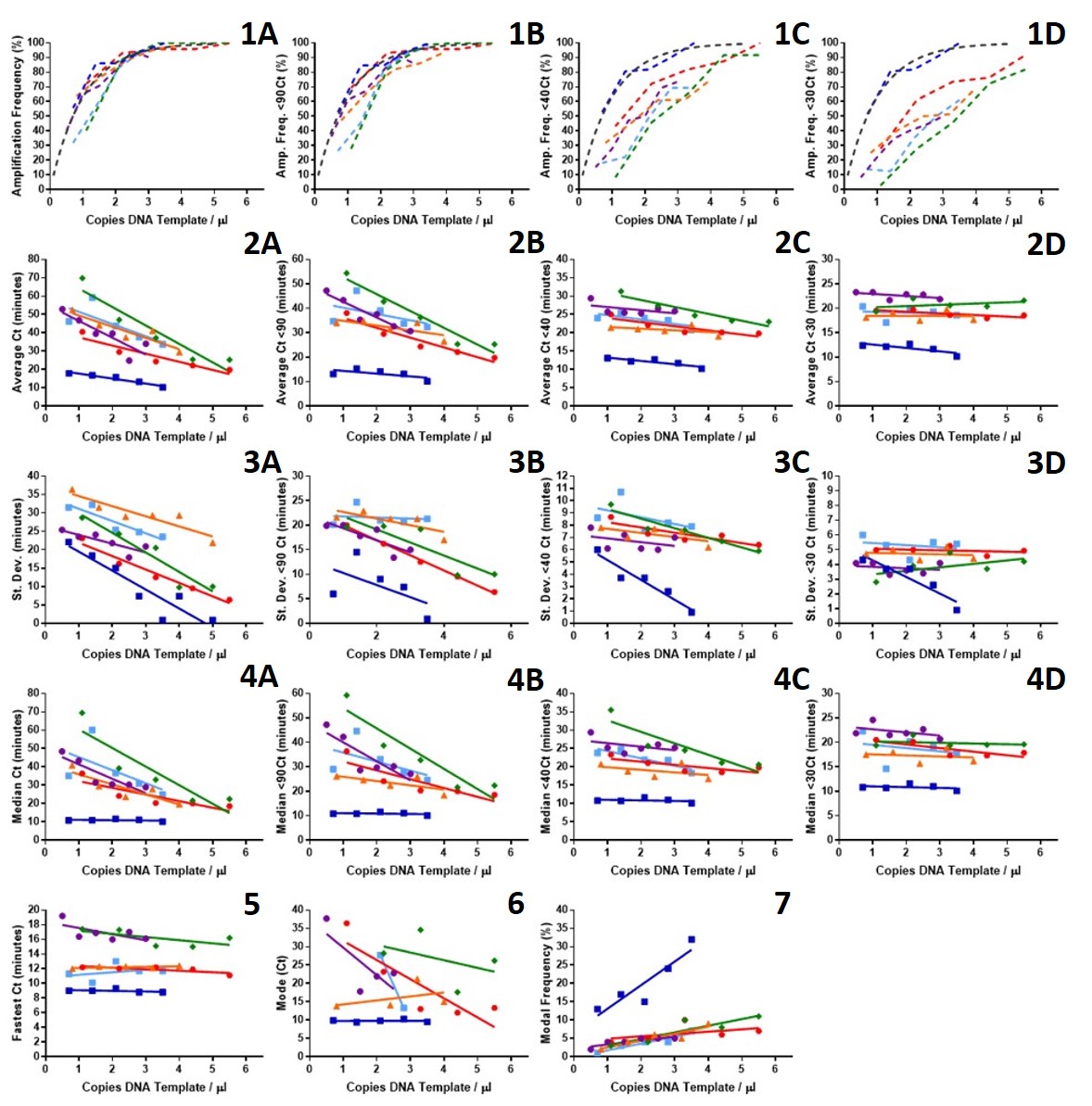


**Figure S26. Combined data for dual detection quenched JOE FIP detection of various templates at low copy numbers normalised to amplification frequency without displacement primers (trendlines).** Predicted results for amplification frequency at low copy number in black, 35Sp artificial template without F3/B3 in dark blue (dashed), 35Sp pART7 linearised plasmid DNA without F3/B3 in orange (dashed), 35Sp native genomic DNA without F3/B3 in red (dashed), 35Sp denatured genomic DNA without F3/B3 in light blue (dashed), NOSt native genomic DNA without F3/B3 in purple (dashed) and NOSt denatured genomic DNA without F3/B3 in green (dashed). Genomic DNA extracted from 5%Bt11 CRM maize. **(A,B,C,D)** Full assay time and truncated to 90, 40 and 30 minutes. **(1)** Amplification frequency, **(2)** average Ct, **(3)** standard deviation, **(4)** median Ct, **(5)** fastest Ct, **(6)** mode Ct and **(7)** the percentage modal frequency (the percentage of replicates comprising the mode Ct against the total number of replicates).


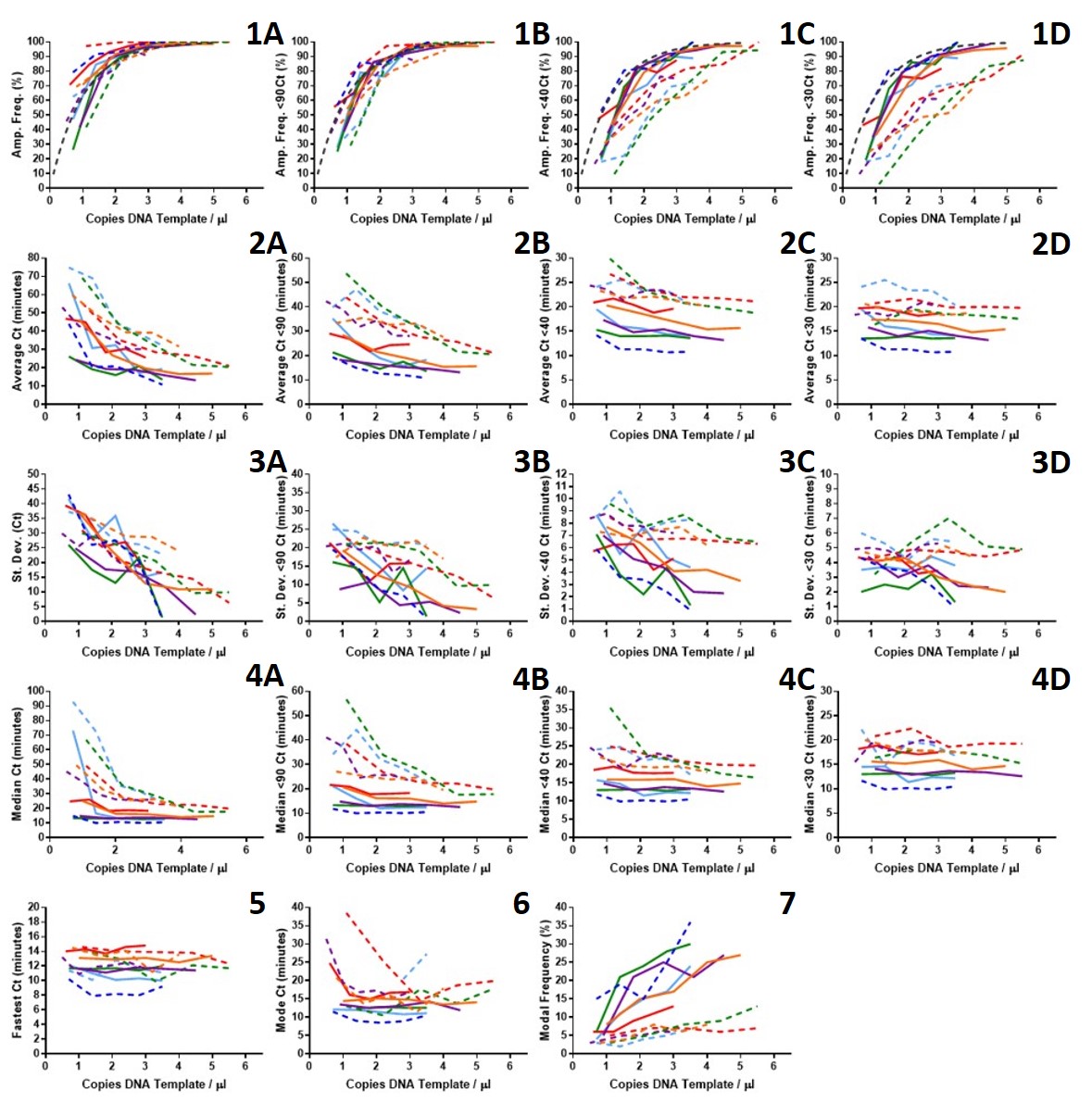


**Figure S27. Combined data for dual detection SYTO9 detection of various templates at low copy numbers normalised to amplification frequency with and without displacement primers.** Predicted results for amplification frequency at low copy number in black, 35Sp artificial template without F3/B3 in dark blue (dashed), 35Sp pART7 linearised plasmid DNA in orange (dashed for without F3/B3), 35Sp native genomic DNA in red (dashed for without F3/B3), 35Sp denatured genomic DNA in light blue (dashed for without F3/B3), NOSt native genomic DNA in purple (dashed for without F3/B3) and NOSt denatured genomic DNA in green (dashed for without F3/B3). Genomic DNA extracted from 5%Bt11 CRM maize. **(A,B,C,D)** Full assay time and truncated to 90, 40 and 30 minutes. **(1)** Amplification frequency, **(2)** average Ct, **(3)** standard deviation, **(4)** median Ct, **(5)** fastest Ct, **(6)** mode Ct and **(7)** the percentage modal frequency (the percentage of replicates comprising the mode Ct against the total number of replicates).


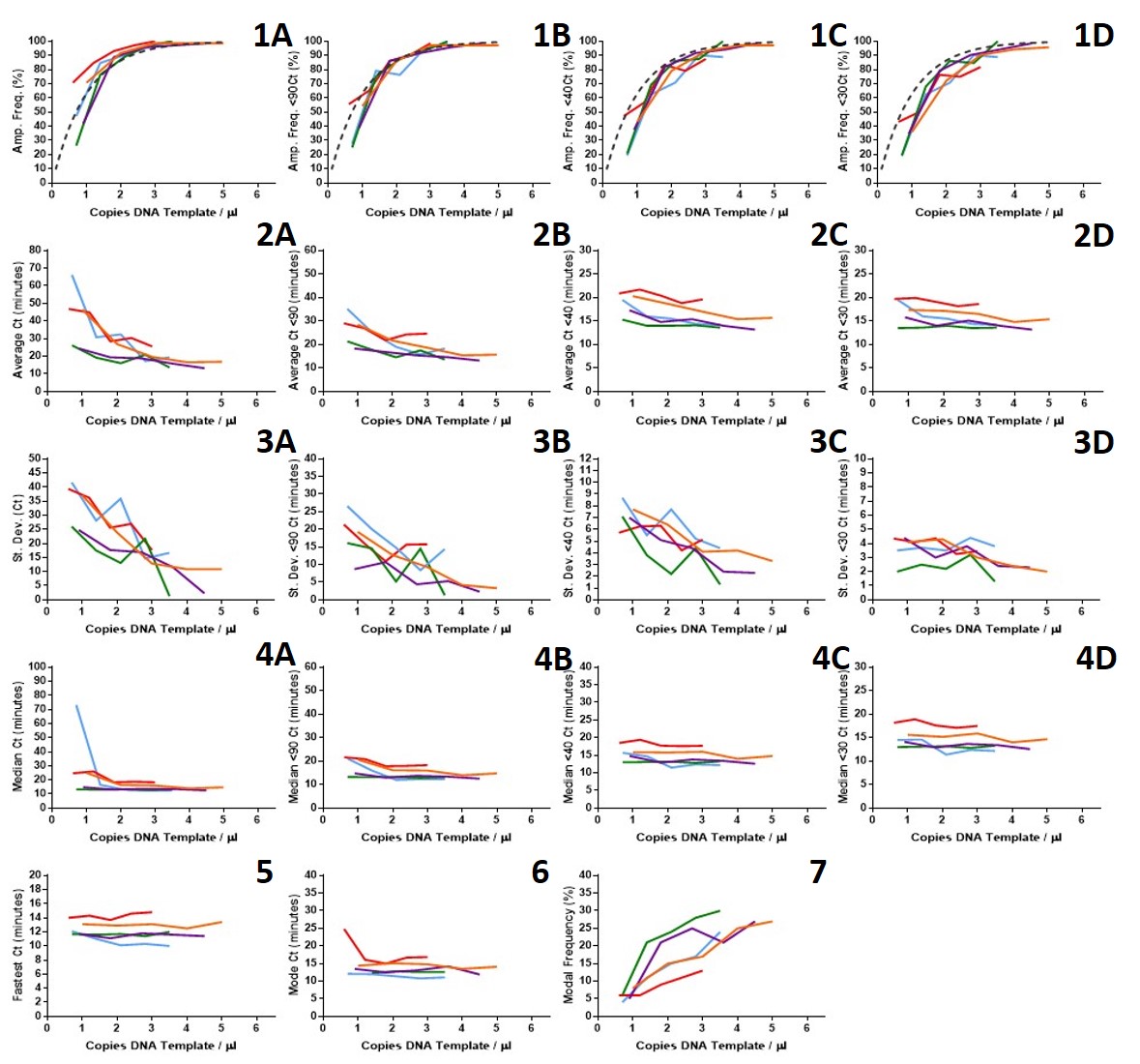


**Figure S28. Combined data for dual detection SYTO9 detection of various templates at low copy numbers normalised to amplification frequency with displacement primers.** Predicted results for amplification frequency at low copy number in black, 35Sp pART7 linearised plasmid DNA in orange, 35Sp native genomic DNA in red, 35Sp denatured genomic DNA in light blue, NOSt native genomic DNA in purple and NOSt denatured genomic DNA in green. Genomic DNA extracted from 5%Bt11 CRM maize. **(A,B,C,D)** Full assay time and truncated to 90, 40 and 30 minutes. **(1)** Amplification frequency, **(2)** average Ct, **(3)** standard deviation, **(4)** median Ct, **(5)** fastest Ct, **(6)** mode Ct and **(7)** the percentage modal frequency (the percentage of replicates comprising the mode Ct against the total number of replicates).


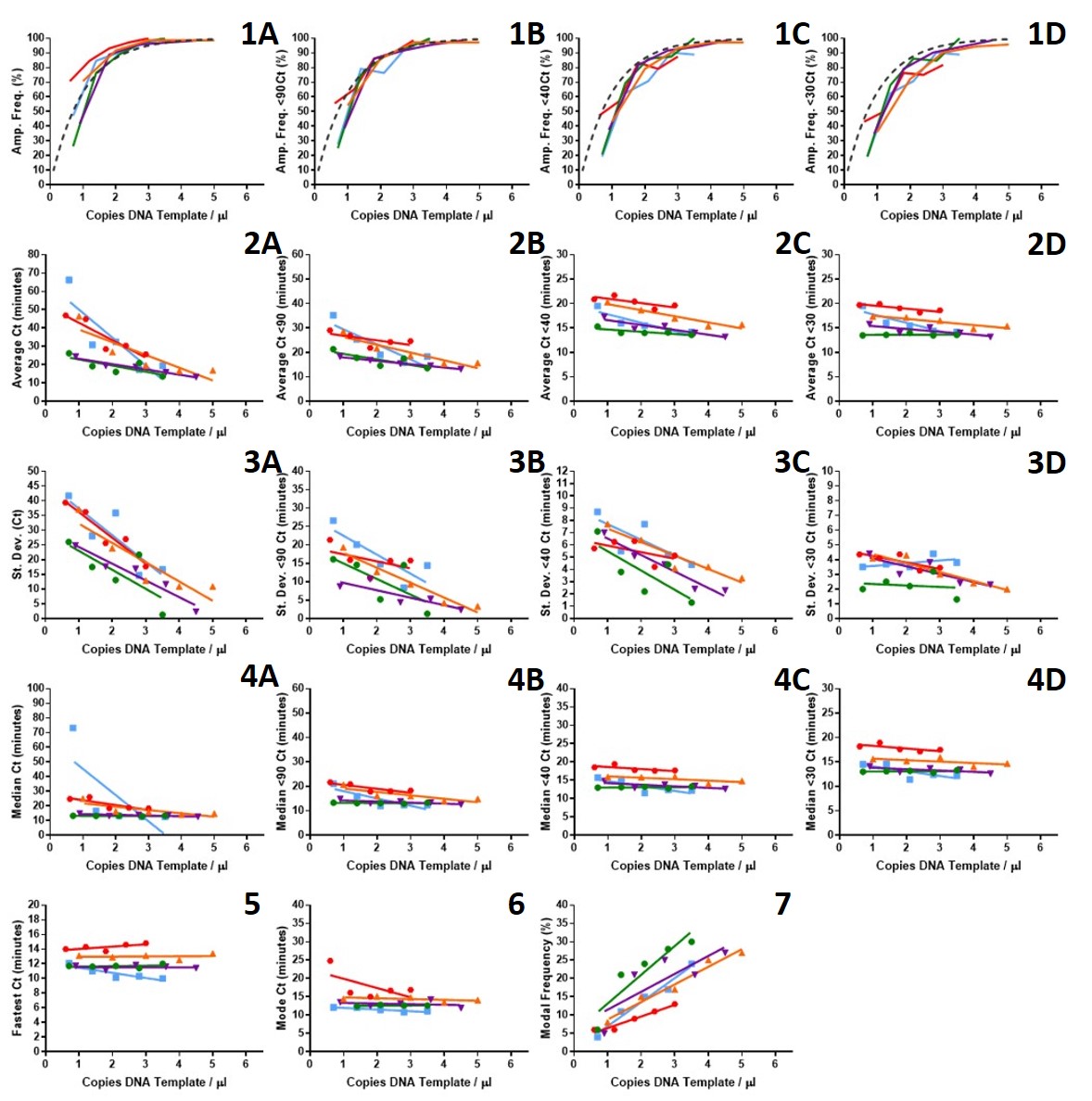


**Figure S29. Combined data for dual detection SYTO9 detection of various templates at low copy numbers normalised to amplification frequency with displacement primers (trendlines).** Predicted results for amplification frequency at low copy number in black, 35Sp pART7 linearised plasmid DNA in orange, 35Sp native genomic DNA in red, 35Sp denatured genomic DNA in light blue, NOSt native genomic DNA in purple and NOSt denatured genomic DNA in green. Genomic DNA extracted from 5%Bt11 CRM maize. **(A,B,C,D)** Full assay time and truncated to 90, 40 and 30 minutes. **(1)** Amplification frequency, **(2)** average Ct, **(3)** standard deviation, **(4)** median Ct, **(5)** fastest Ct, **(6)** mode Ct and **(7)** the percentage modal frequency (the percentage of replicates comprising the mode Ct against the total number of replicates).


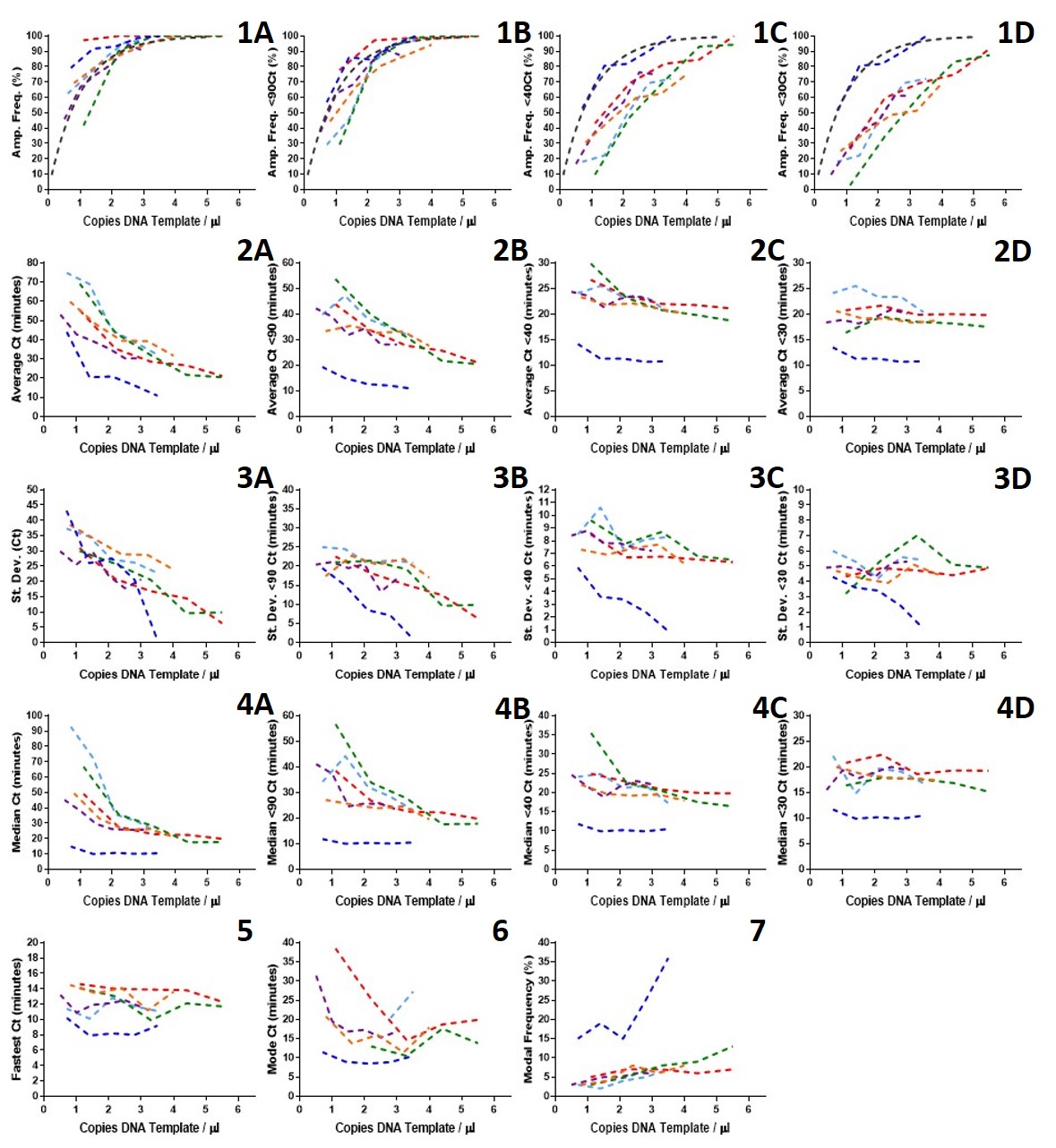


**Figure S30. Combined data for dual detection SYTO9 detection of various templates at low copy numbers normalised to amplification frequency without displacement primers.** Predicted results for amplification frequency at low copy number in black, 35Sp artificial template without F3/B3 in dark blue (dashed), 35Sp pART7 linearised plasmid DNA without F3/B3 in orange (dashed), 35Sp native genomic DNA without F3/B3 in red (dashed), 35Sp denatured genomic DNA without F3/B3 in light blue (dashed), NOSt native genomic DNA without F3/B3 in purple (dashed) and NOSt denatured genomic DNA without F3/B3 in green (dashed). Genomic DNA extracted from 5%Bt11 CRM maize. **(A,B,C,D)** Full assay time and truncated to 90, 40 and 30 minutes. **(1)** Amplification frequency, **(2)** average Ct, **(3)** standard deviation, **(4)** median Ct, **(5)** fastest Ct, **(6)** mode Ct and **(7)** the percentage modal frequency (the percentage of replicates comprising the mode Ct against the total number of replicates).


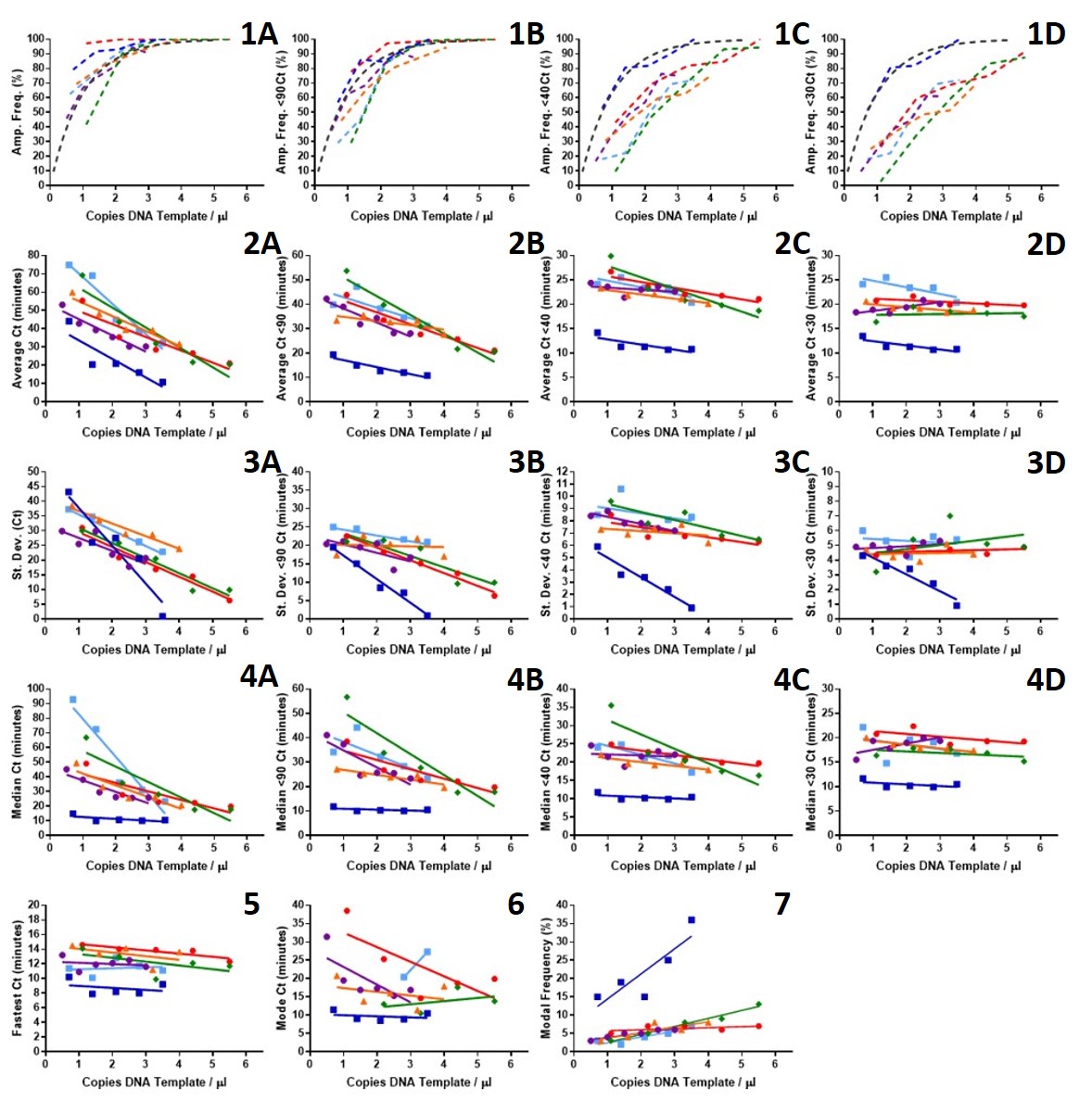


**Figure S31. Combined data for dual detection SYTO9 detection of various templates at low copy numbers normalised to amplification frequency without displacement primers (trendlines).** Predicted results for amplification frequency at low copy number in black, 35Sp artificial template without F3/B3 in dark blue (dashed), 35Sp pART7 linearised plasmid DNA without F3/B3 in orange (dashed), 35Sp native genomic DNA without F3/B3 in red (dashed), 35Sp denatured genomic DNA without F3/B3 in light blue (dashed), NOSt native genomic DNA without F3/B3 in purple (dashed) and NOSt denatured genomic DNA without F3/B3 in green (dashed). Genomic DNA extracted from 5%Bt11 CRM maize. **(A,B,C,D)** Full assay time and truncated to 90, 40 and 30 minutes. **(1)** Amplification frequency, **(2)** average Ct, **(3)** standard deviation, **(4)** median Ct, **(5)** fastest Ct, **(6)** mode Ct and **(7)** the percentage modal frequency (the percentage of replicates comprising the mode Ct against the total number of replicates).

**
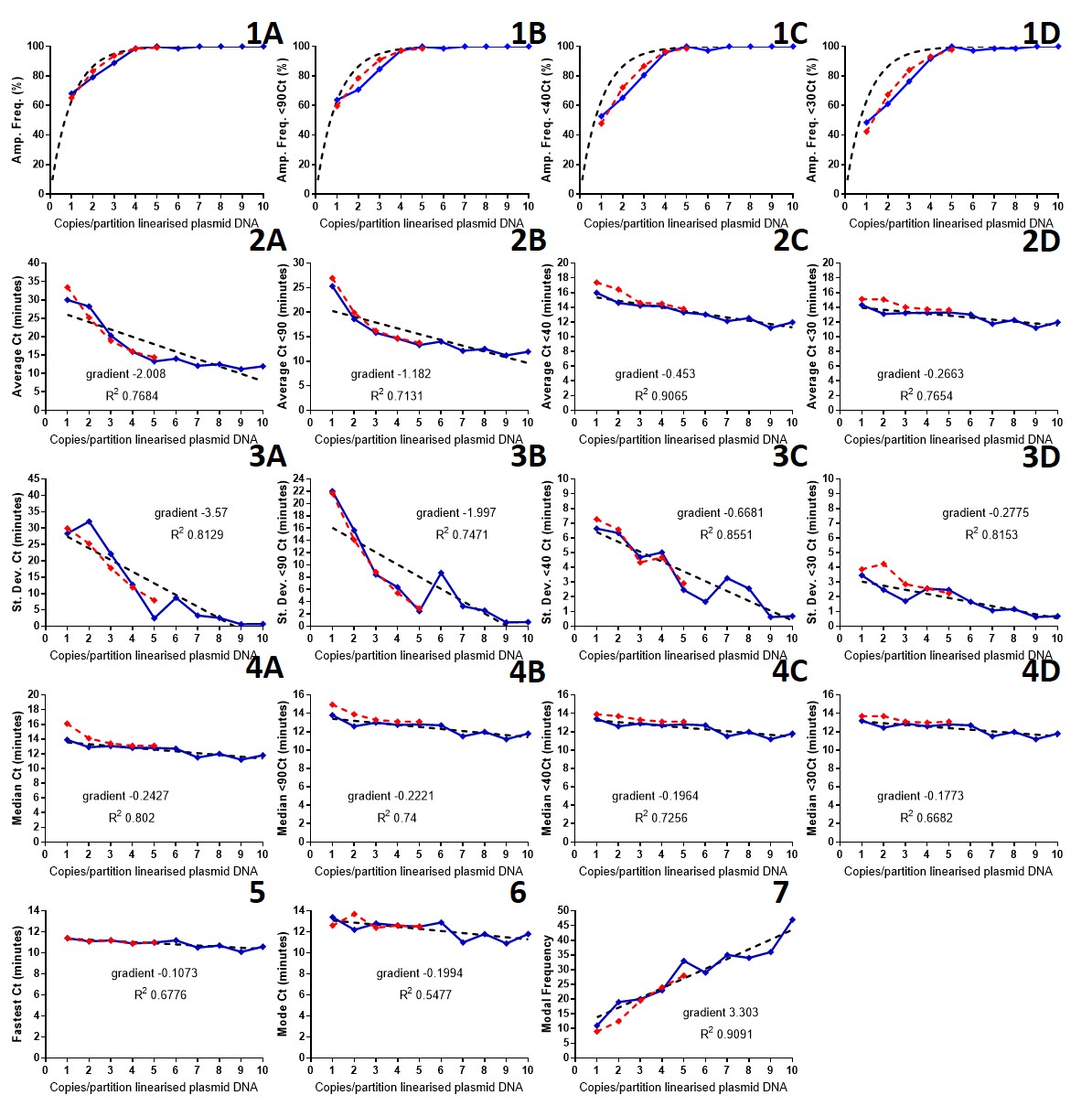
**

**Figure S32. Dual detection quenched JOE-FIP detection of linearised plasmid template at 1 to 10 copy numbers normalised to amplification frequency.**  Results from the amplification of 35S promoter sequence of linearised plasmid DNA template from 1 to 10 copies in blue, predicted results in black and previous results from linearised plasmid DNA template in red. **(A,B,C,D)** Full assay time and truncated to 90, 40 and 30 minutes. **(1)** Amplification frequency, **(2)** average Ct, **(3)** standard deviation, **(4)** median Ct, **(5)** fastest Ct, **(6)** mode Ct and **(7)** the percentage modal frequency (the percentage of replicates comprising the mode Ct against the total number of replicates).


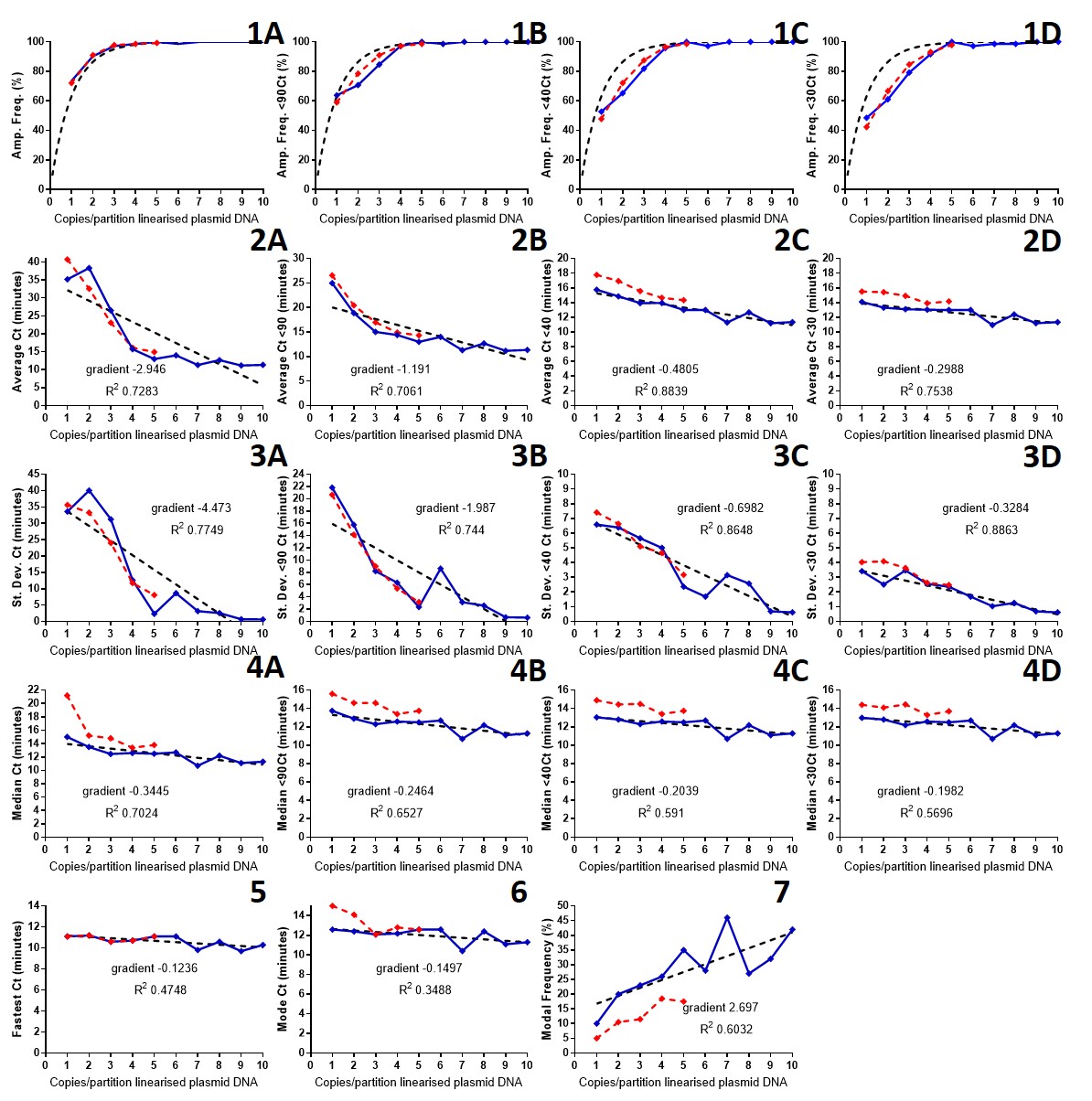


**Figure S33. Dual detection SYTO9 detection of li nearised plasmid template at 1 to 10 copy numbers normalised to amplification frequency.**  Results from the amplification of 35S promoter sequence of linearised plasmid DNA template from 1 to 10 copies in blue, predicted results in black and previous results from linearised plasmid DNA template in red. **(A,B,C,D)** Full assay time and truncated to 90, 40 and 30 minutes. **(1)** Amplification frequency, **(2)** average Ct, **(3)** standard deviation, **(4)** median Ct, **(5)** fastest Ct, **(6)** mode Ct and **(7)** the percentage modal frequency (the percentage of replicates comprising the mode Ct against the total number of replicates).


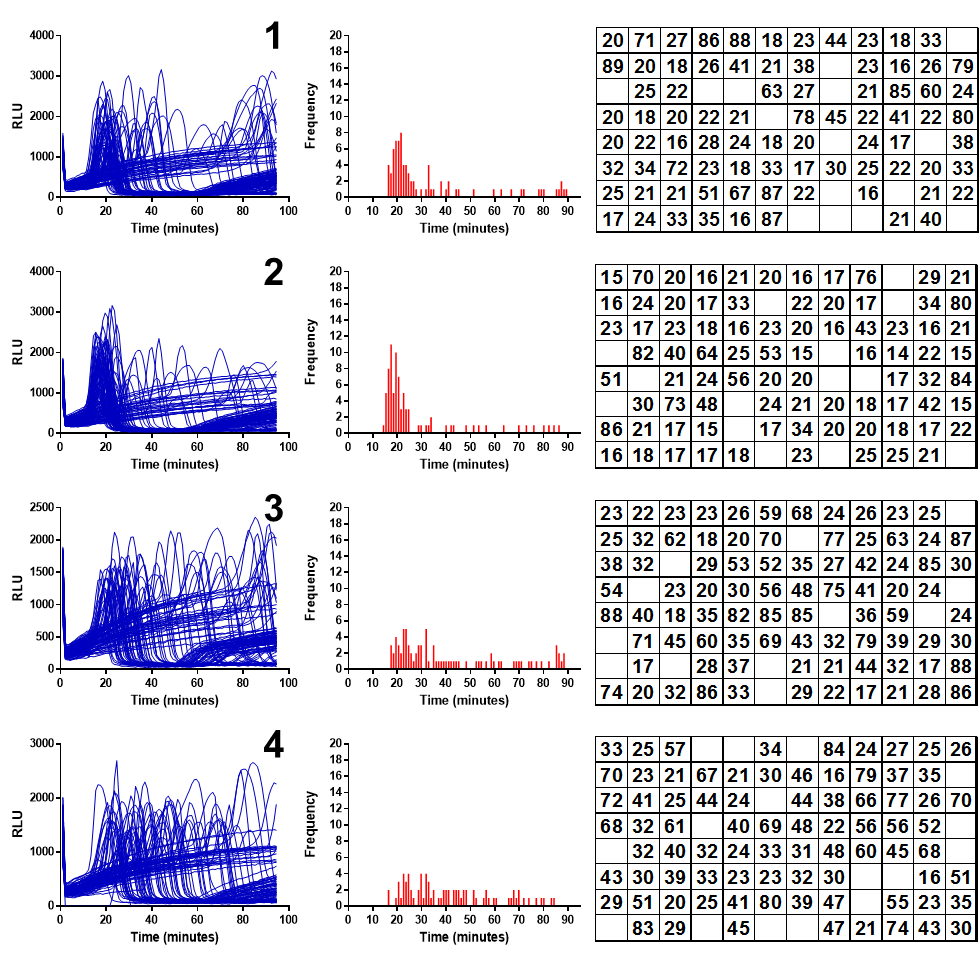


**Figure S34. 35Sp LAMP BART 1copy 5%Bt11.** **(1)** Native template with full 35Sp primer set, **(2)** denatured template with full primer set, **(3)** native template with F3 and B3 omitted and **(4)** denatured template with F3 and B3 omitted.


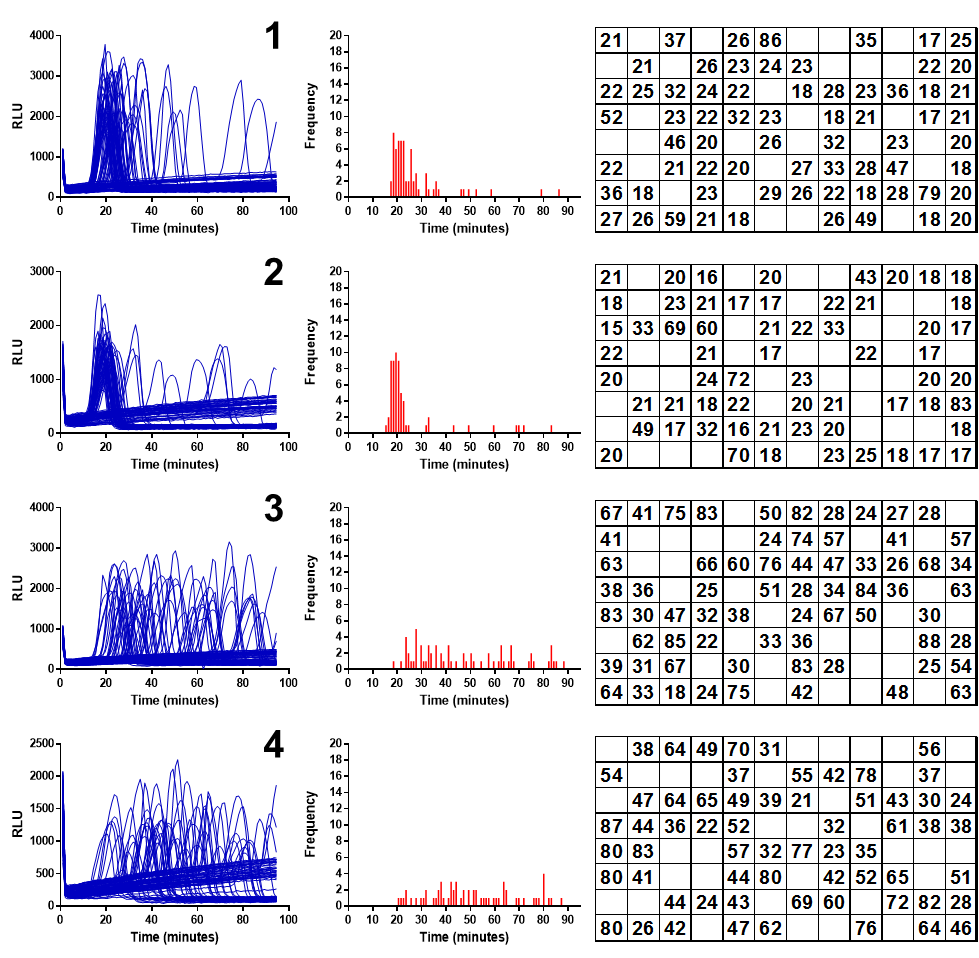


**Figure S35. NOSt LAMP BART 1copy 5%Bt11.** **(1)** Native template with full NOSt primer set, **(2)** denatured template with full primer set, **(3)** native template with F3 and B3 omitted and **(4)** denatured template with F3 and B3 omitted.
